# Supplementary material for: Neuraminidase inhibition promotes the collective migration of neurons and recovery of brain function
Source: EMBO Mol Med. 2024 May 24;16(6):1228–53. doi: 10.1038/s44321-024-00073-7 (PMC11178813; doi:10.1038/s44321-024-00073-7)
Supplement: Supplementary file 1 — Movie EV1 [file 44321_2024_73_MOESM1_ESM.zip › Movie EV1/Movie EV1_Legend.docx]

**Movie EV1:** Three-dimensional reconstruction of neuronal chains and non-adherent area in the adult RMS.
