# Supplementary material for: Neuraminidase inhibition promotes the collective migration of neurons and recovery of brain function
Source: EMBO Mol Med. 2024 May 24;16(6):1228–53. doi: 10.1038/s44321-024-00073-7 (PMC11178813; doi:10.1038/s44321-024-00073-7)
Supplement: Supplementary file 5 — Movie EV5 [file 44321_2024_73_MOESM5_ESM.zip › Movie EV5/Movie EV5_Legend.docx]

**Movie EV5:** Time-lapse imaging of control EGFP+ cells (without LPS), and EGFP+ cells treated with PBS+LPS, DANA+LPS or zanamivir+ LPS and co-cultured with BV2 cells.
