# Supplementary figures and images for: Neuraminidase inhibition promotes the collective migration of neurons and recovery of brain function (part 1 of 2)
Source: EMBO Mol Med. 2024 May 24;16(6):1228–53. doi: 10.1038/s44321-024-00073-7 (PMC11178813; doi:10.1038/s44321-024-00073-7)

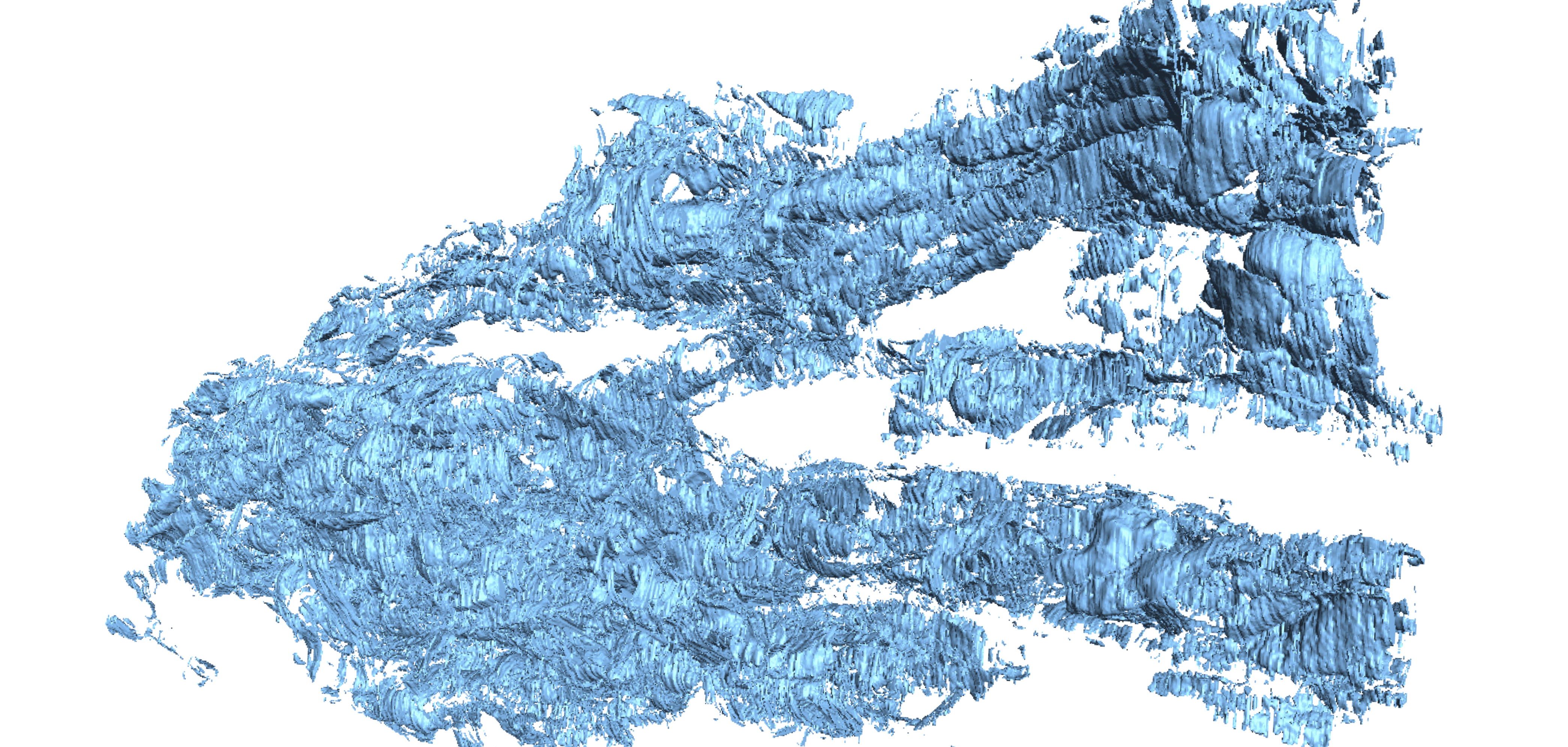

Supplement: Supplementary file 6 — Source data Fig. 1 [file 44321_2024_73_MOESM6_ESM.zip › Figure 1/1A/1A' Non-adherent area in the adult RMS.tif]

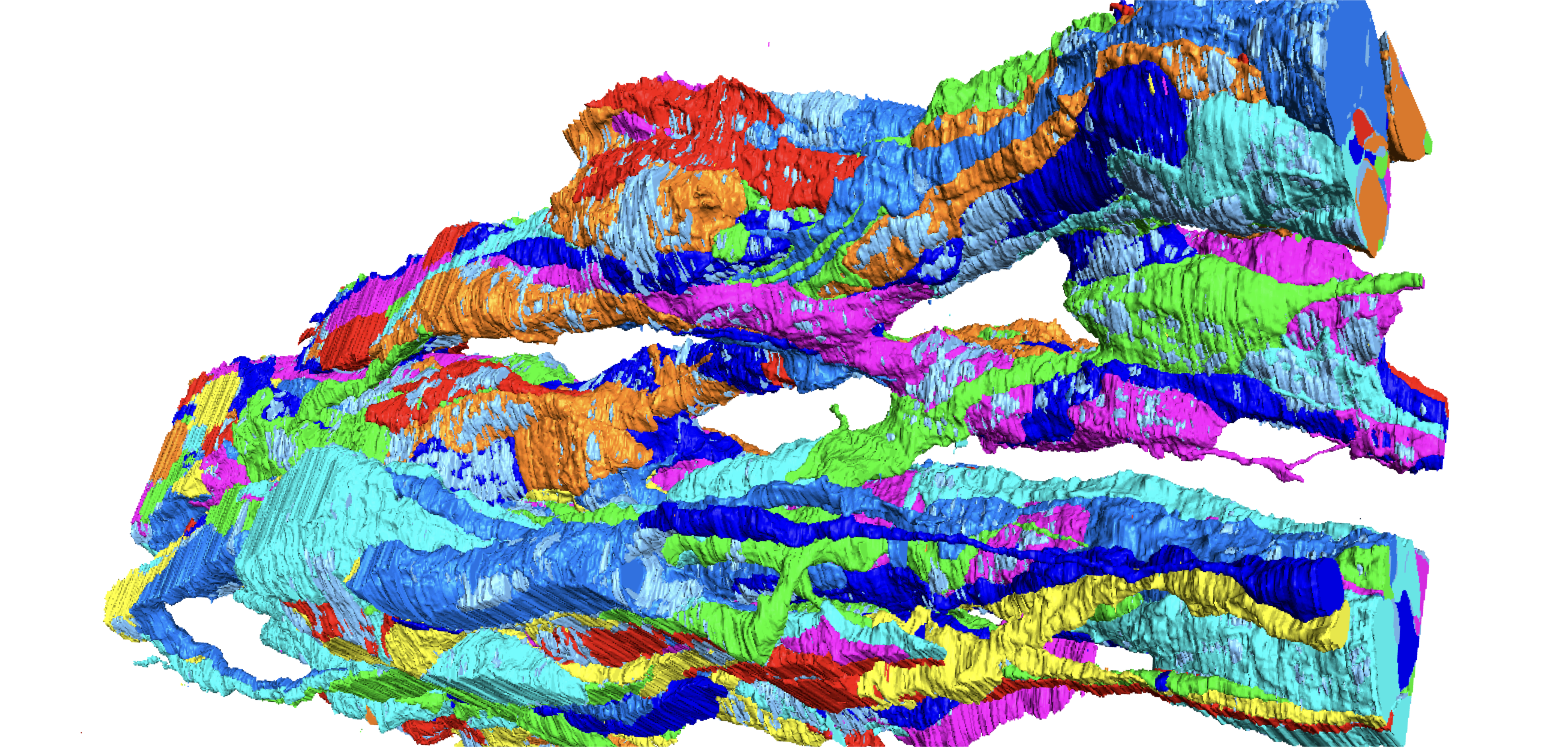

Supplement: Supplementary file 6 — Source data Fig. 1 [file 44321_2024_73_MOESM6_ESM.zip › Figure 1/1A/1A Neuronal chain in the adult RMS.tif]

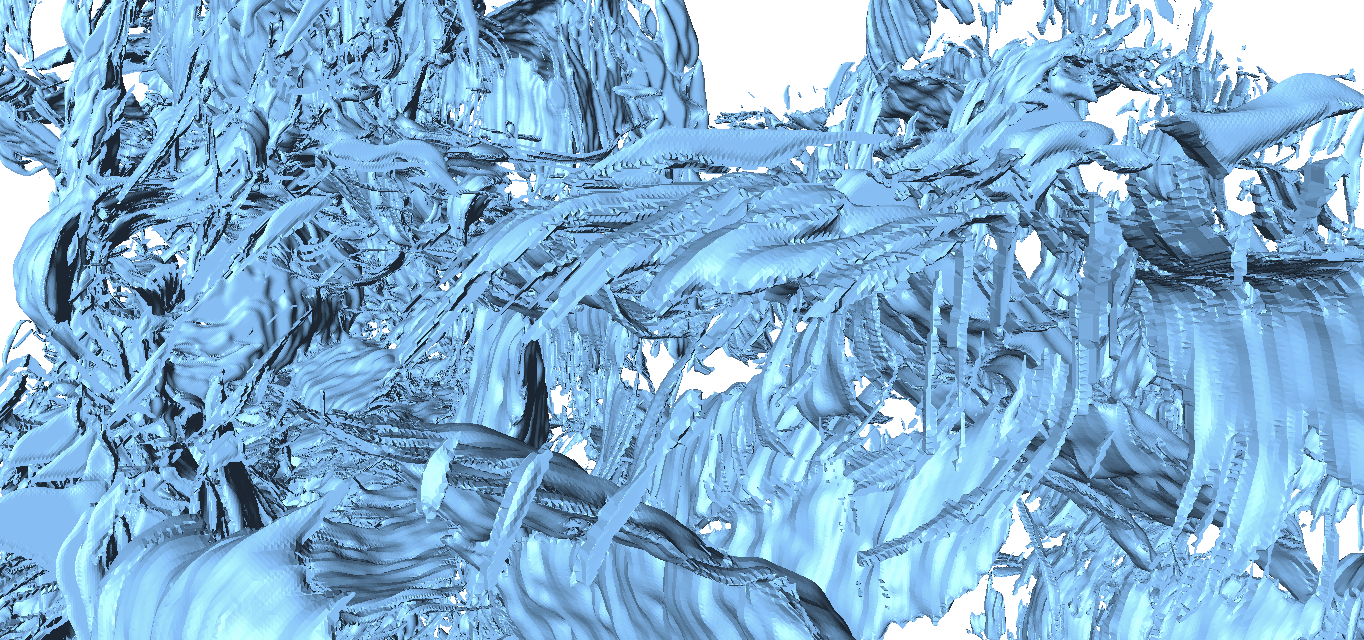

Supplement: Supplementary file 6 — Source data Fig. 1 [file 44321_2024_73_MOESM6_ESM.zip › Figure 1/1B/1B'' Non-adherent area in chain.tif]

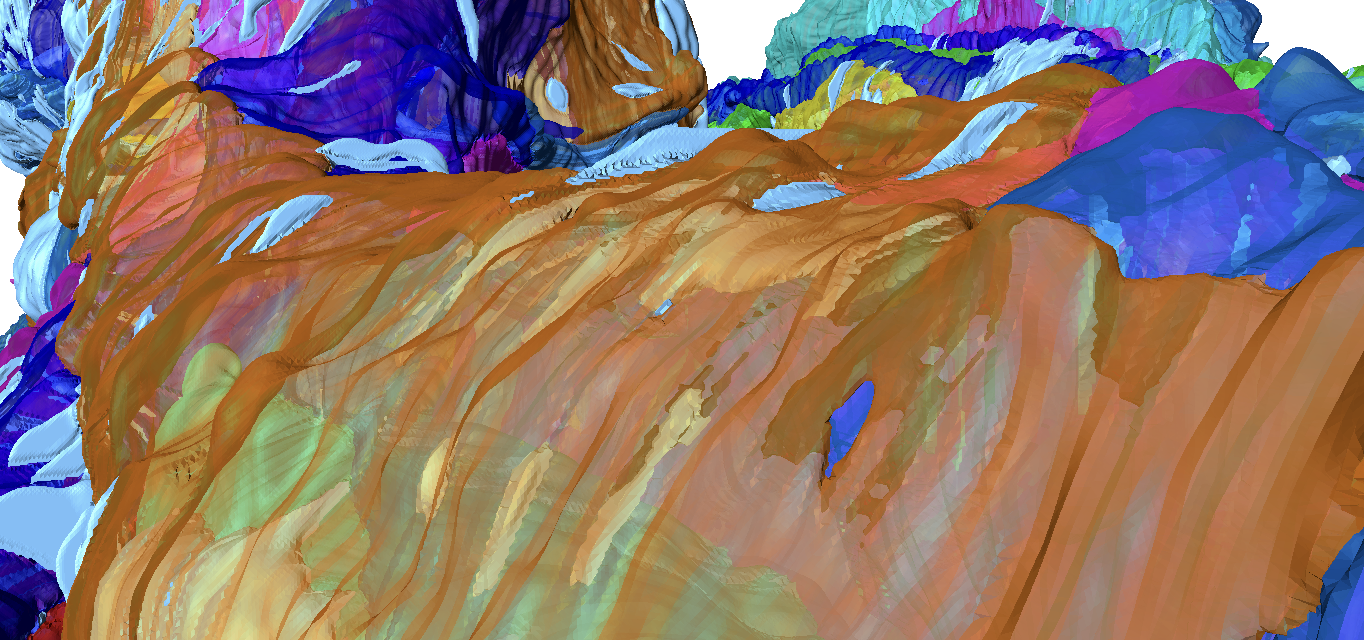

Supplement: Supplementary file 6 — Source data Fig. 1 [file 44321_2024_73_MOESM6_ESM.zip › Figure 1/1B/1B' New neuron and non-adherent area in chain.tif]

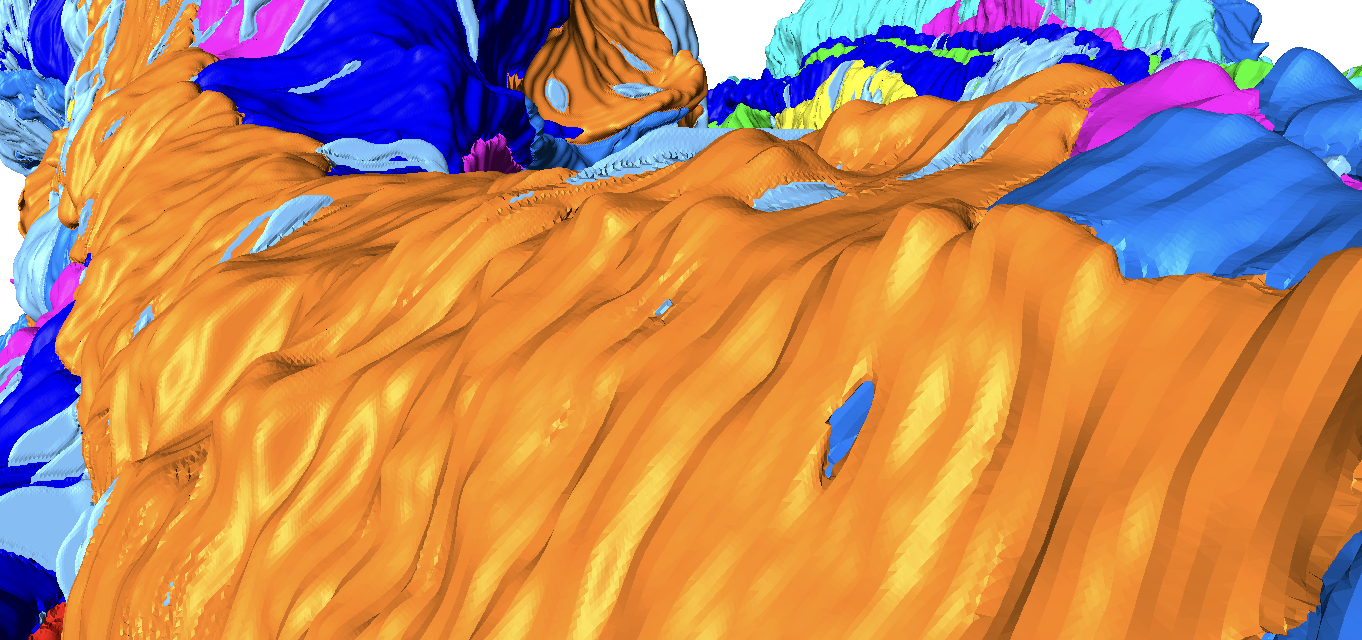

Supplement: Supplementary file 6 — Source data Fig. 1 [file 44321_2024_73_MOESM6_ESM.zip › Figure 1/1B/1B New neuron and non-adherent area in chain.tif]

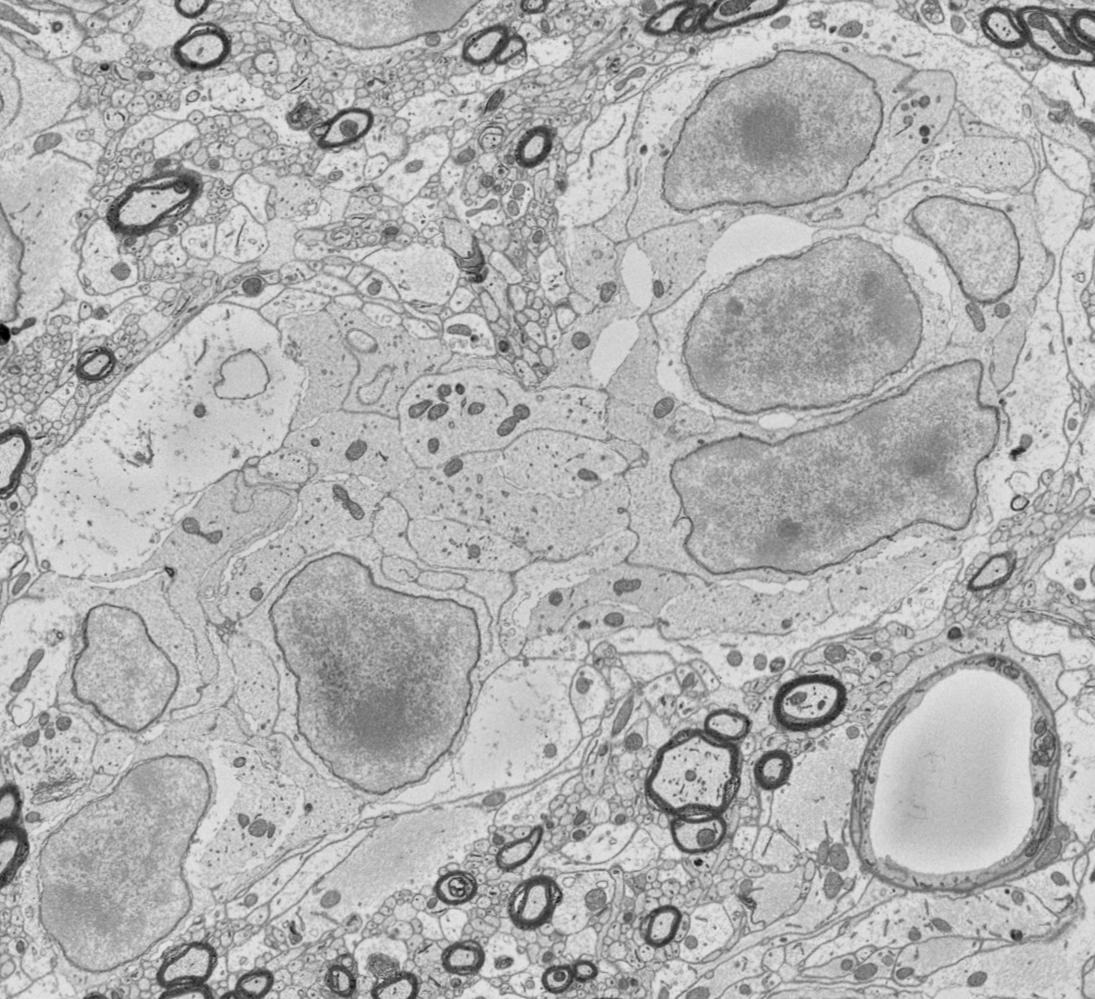

Supplement: Supplementary file 6 — Source data Fig. 1 [file 44321_2024_73_MOESM6_ESM.zip › Figure 1/1E/1E Neuronal chain.tif]

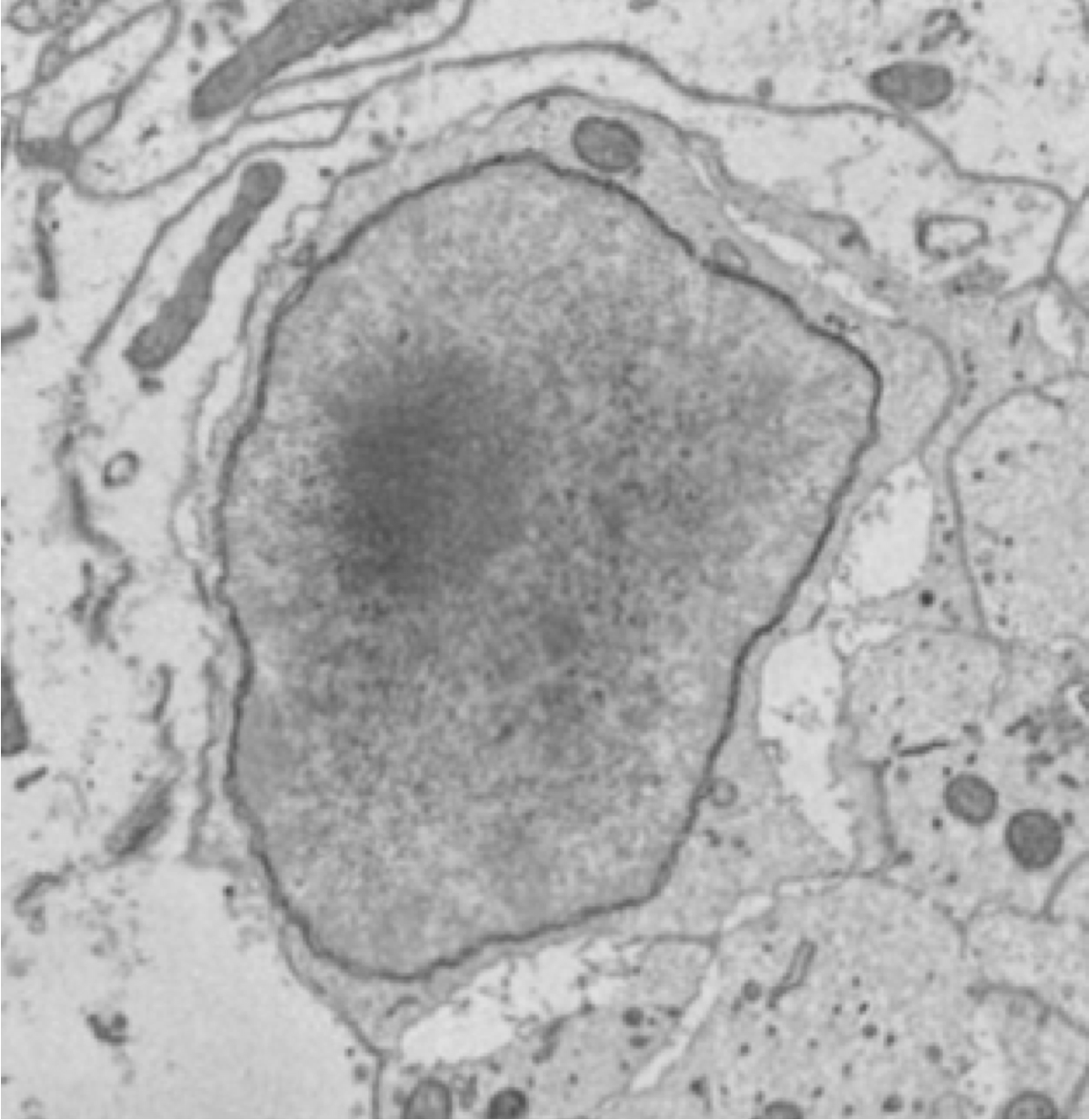

Supplement: Supplementary file 6 — Source data Fig. 1 [file 44321_2024_73_MOESM6_ESM.zip › Figure 1/1C/1C New neurons and astrocytes in normal RMS.tif]

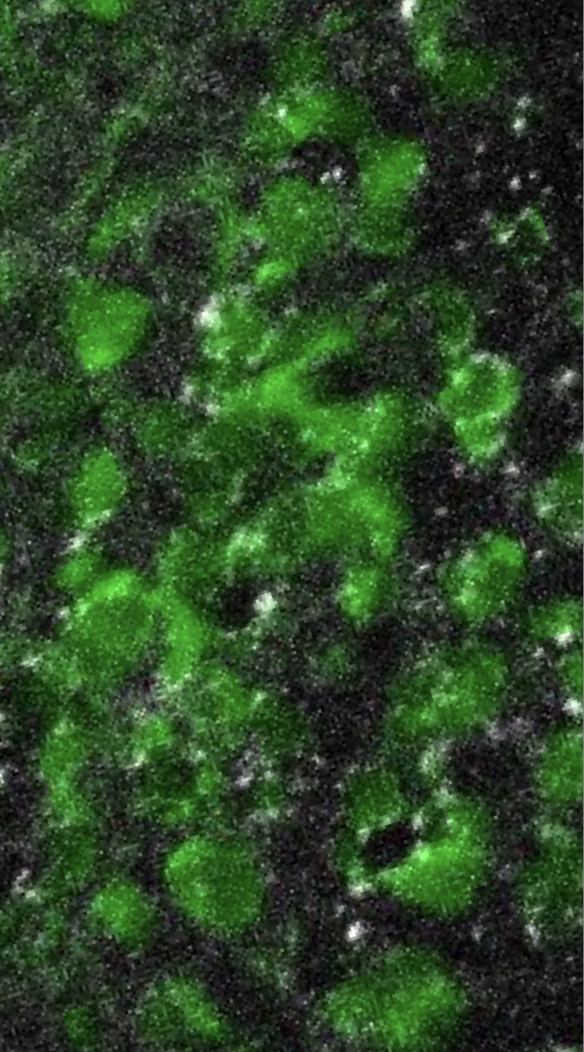

Supplement: Supplementary file 7 — Source data Fig. 2 [file 44321_2024_73_MOESM7_ESM.zip › Figure 2/2R/2R EndoN Dcx-GFP, PSA-NCAM.tif]

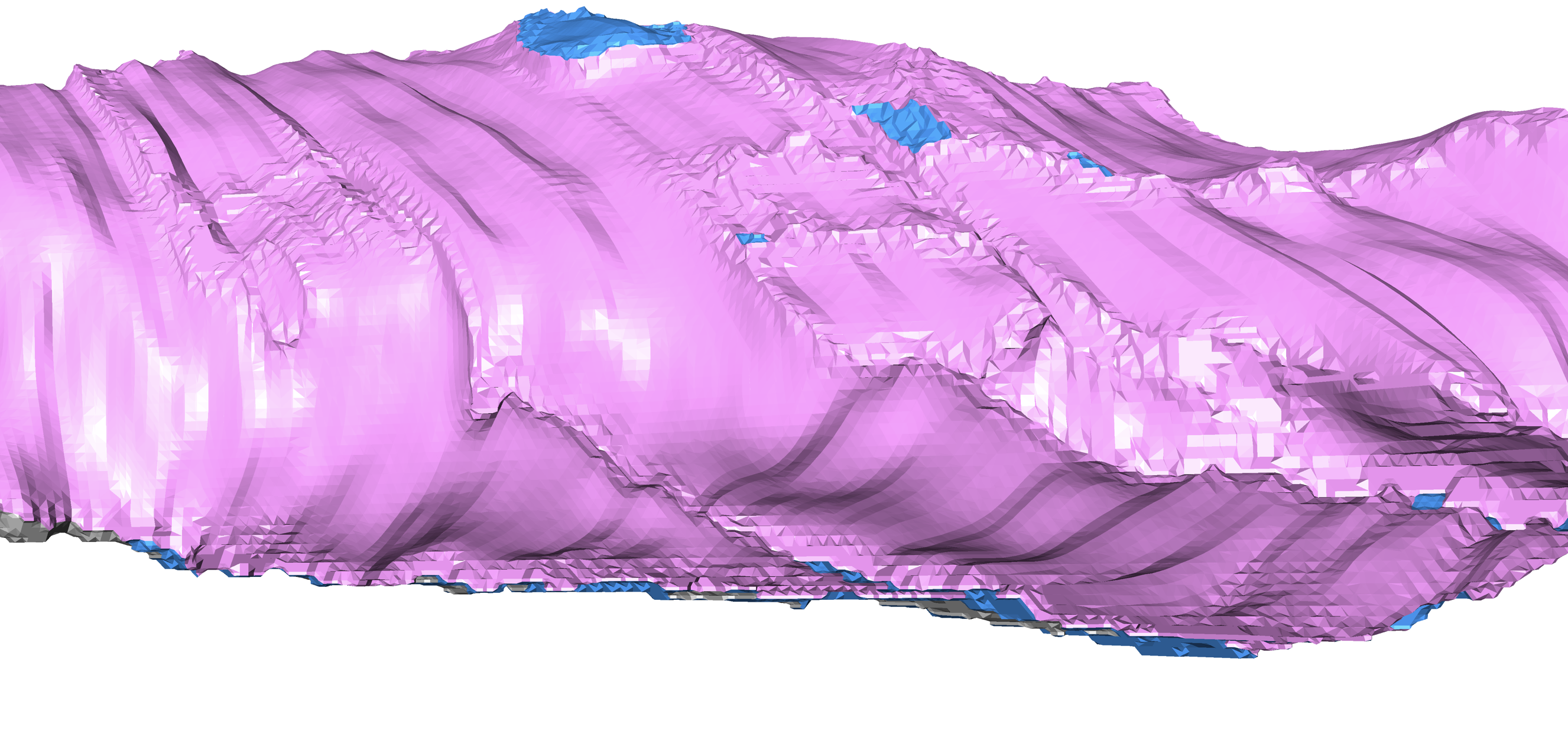

Supplement: Supplementary file 7 — Source data Fig. 2 [file 44321_2024_73_MOESM7_ESM.zip › Figure 2/2G/2G Injured brain.tif]

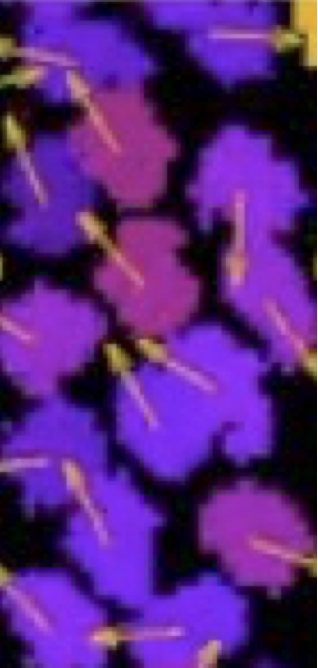

Supplement: Supplementary file 7 — Source data Fig. 2 [file 44321_2024_73_MOESM7_ESM.zip › Figure 2/2I/2I Weak adhesion-2.tif]

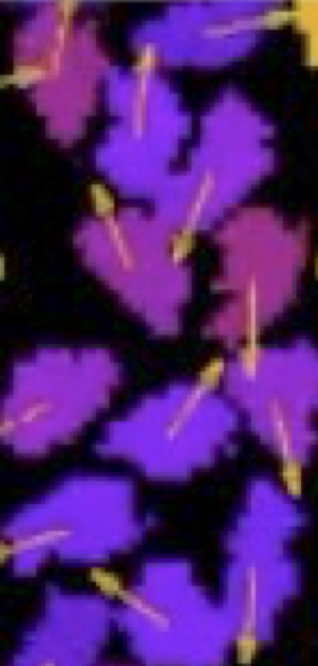

Supplement: Supplementary file 7 — Source data Fig. 2 [file 44321_2024_73_MOESM7_ESM.zip › Figure 2/2I/2I Weak adhesion-3.tif]

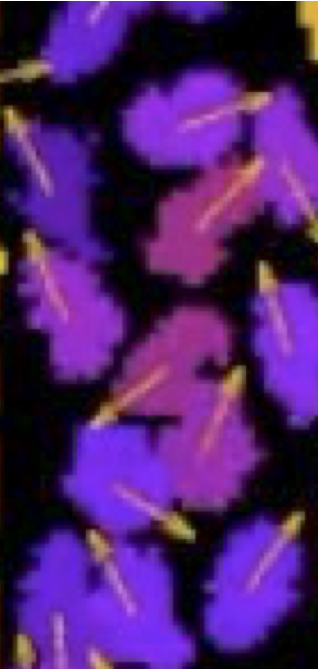

Supplement: Supplementary file 7 — Source data Fig. 2 [file 44321_2024_73_MOESM7_ESM.zip › Figure 2/2I/2I Weak adhesion-1.tif]

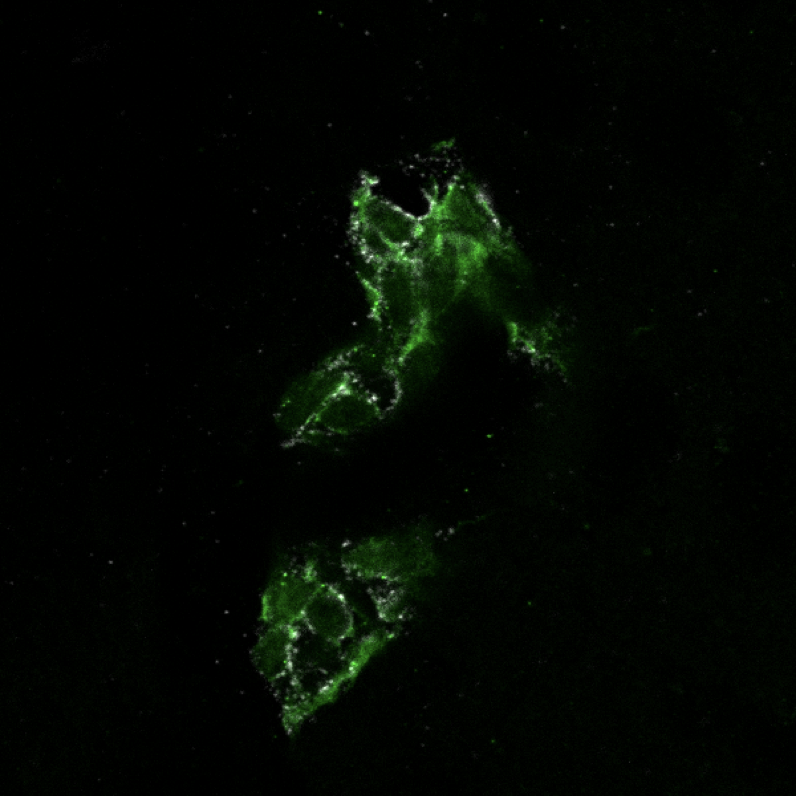

Supplement: Supplementary file 7 — Source data Fig. 2 [file 44321_2024_73_MOESM7_ESM.zip › Figure 2/2N/2N Injured brain Dcx, PSA-NCAM.tif]

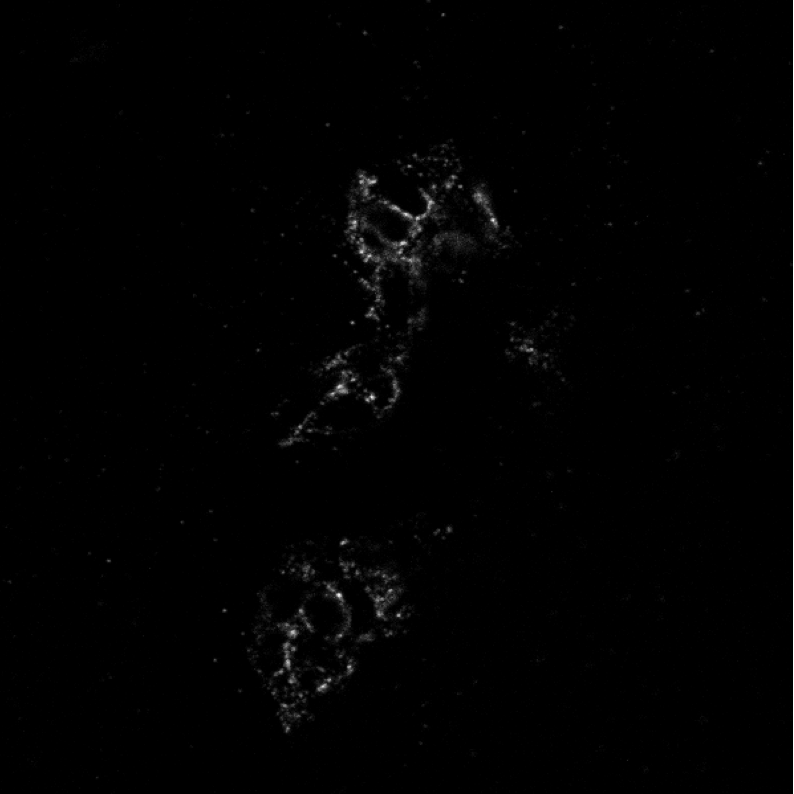

Supplement: Supplementary file 7 — Source data Fig. 2 [file 44321_2024_73_MOESM7_ESM.zip › Figure 2/2N/2N' Injured brain PSA-NCAM.tif]

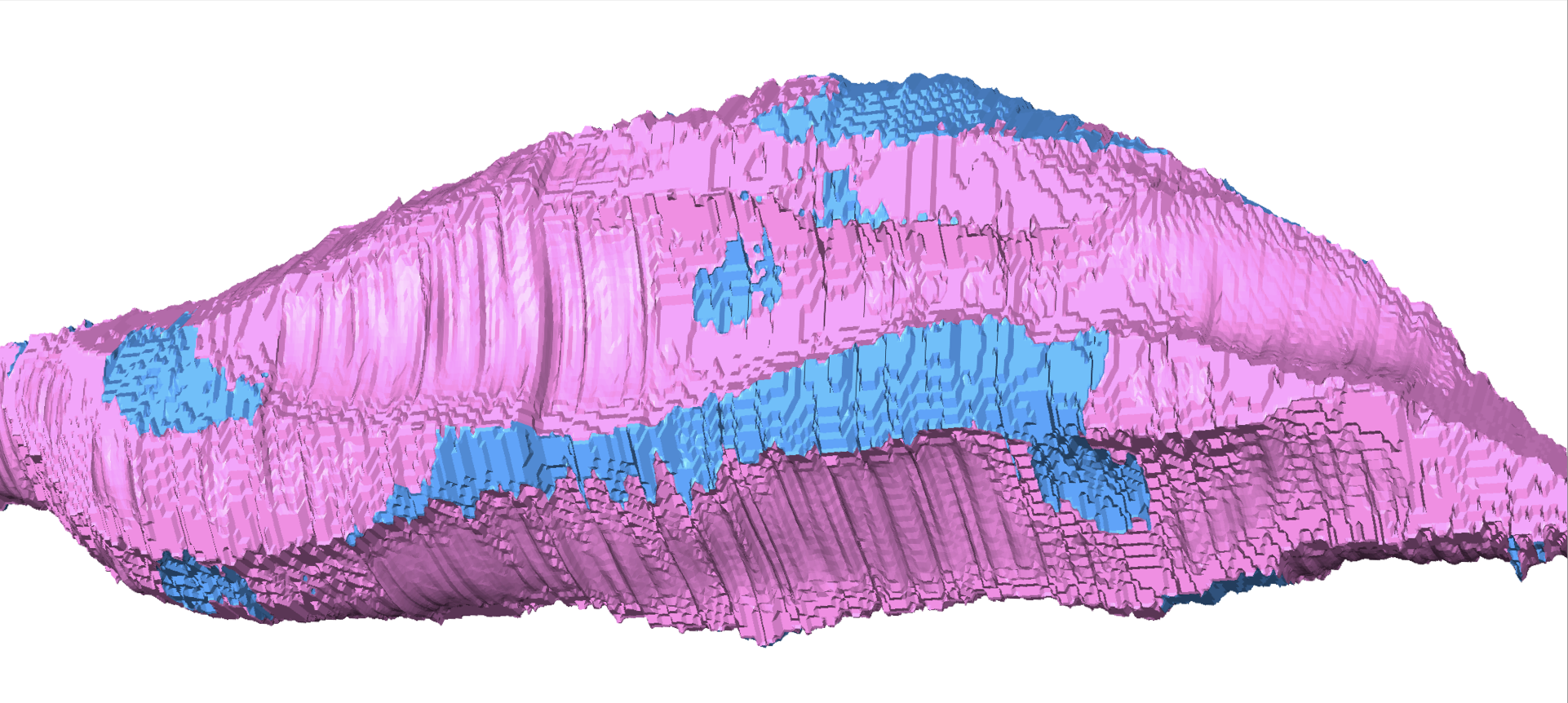

Supplement: Supplementary file 7 — Source data Fig. 2 [file 44321_2024_73_MOESM7_ESM.zip › Figure 2/2S/2S EndoN.tif]

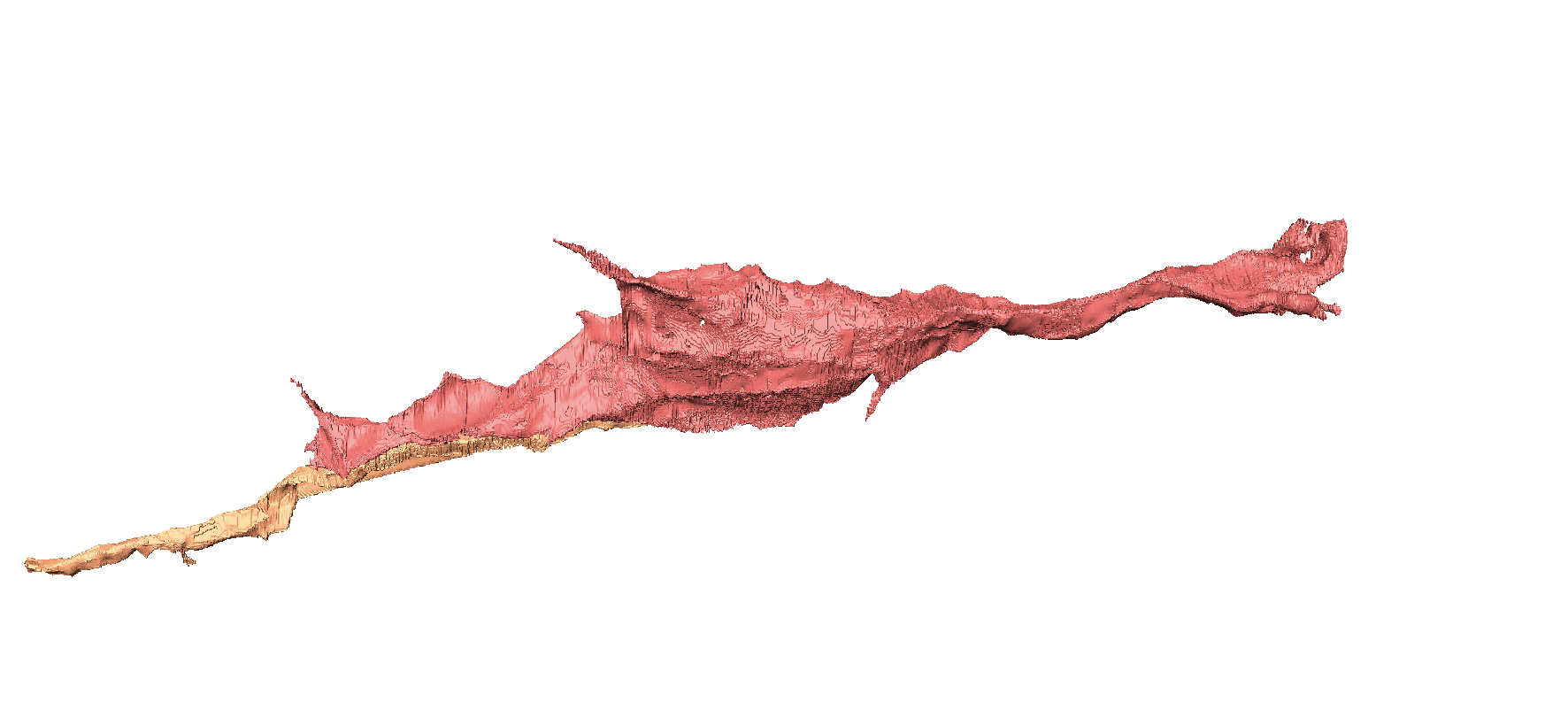

Supplement: Supplementary file 7 — Source data Fig. 2 [file 44321_2024_73_MOESM7_ESM.zip › Figure 2/2A/2A'' New neuron oriented in the reverse direction-2.tif]

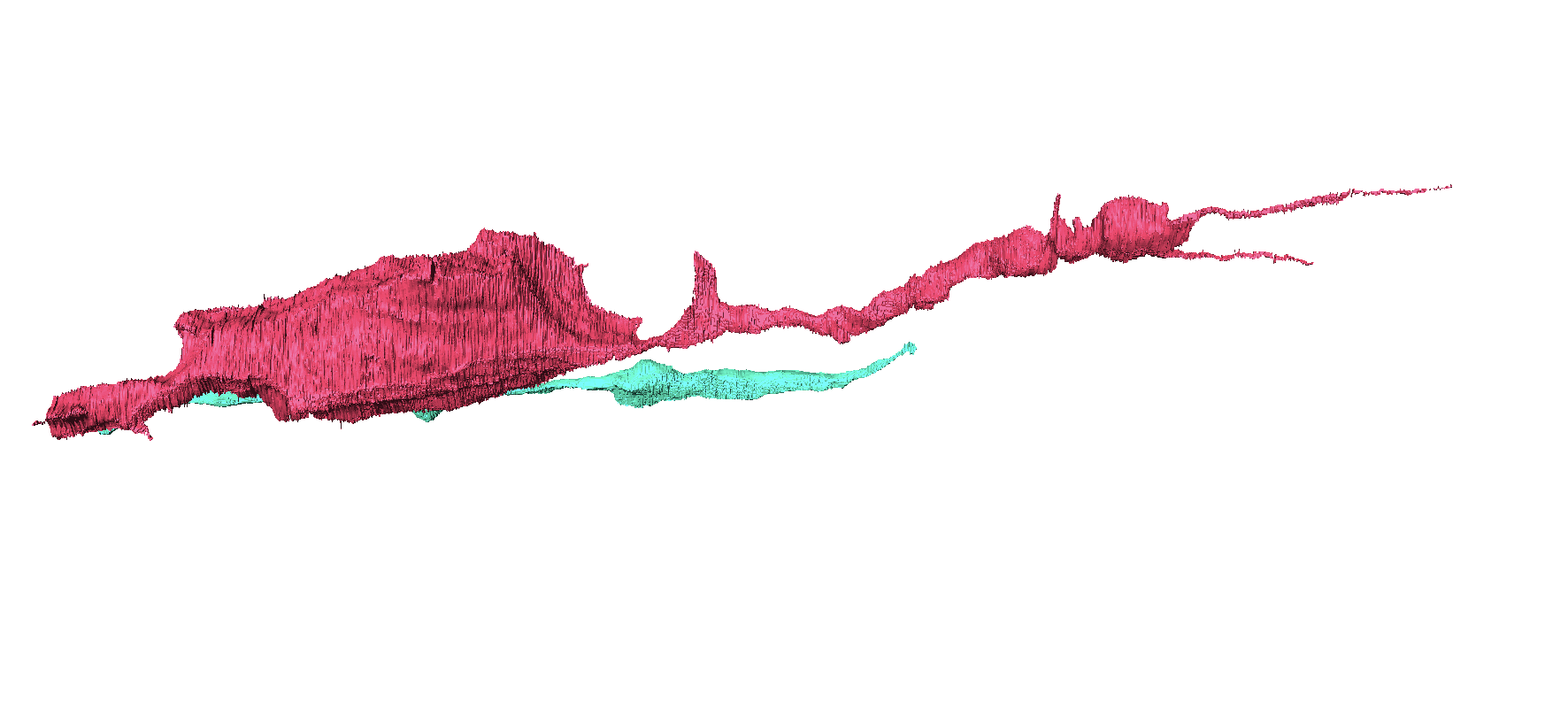

Supplement: Supplementary file 7 — Source data Fig. 2 [file 44321_2024_73_MOESM7_ESM.zip › Figure 2/2A/2A'' New neuron oriented in the reverse direction-1.tif]

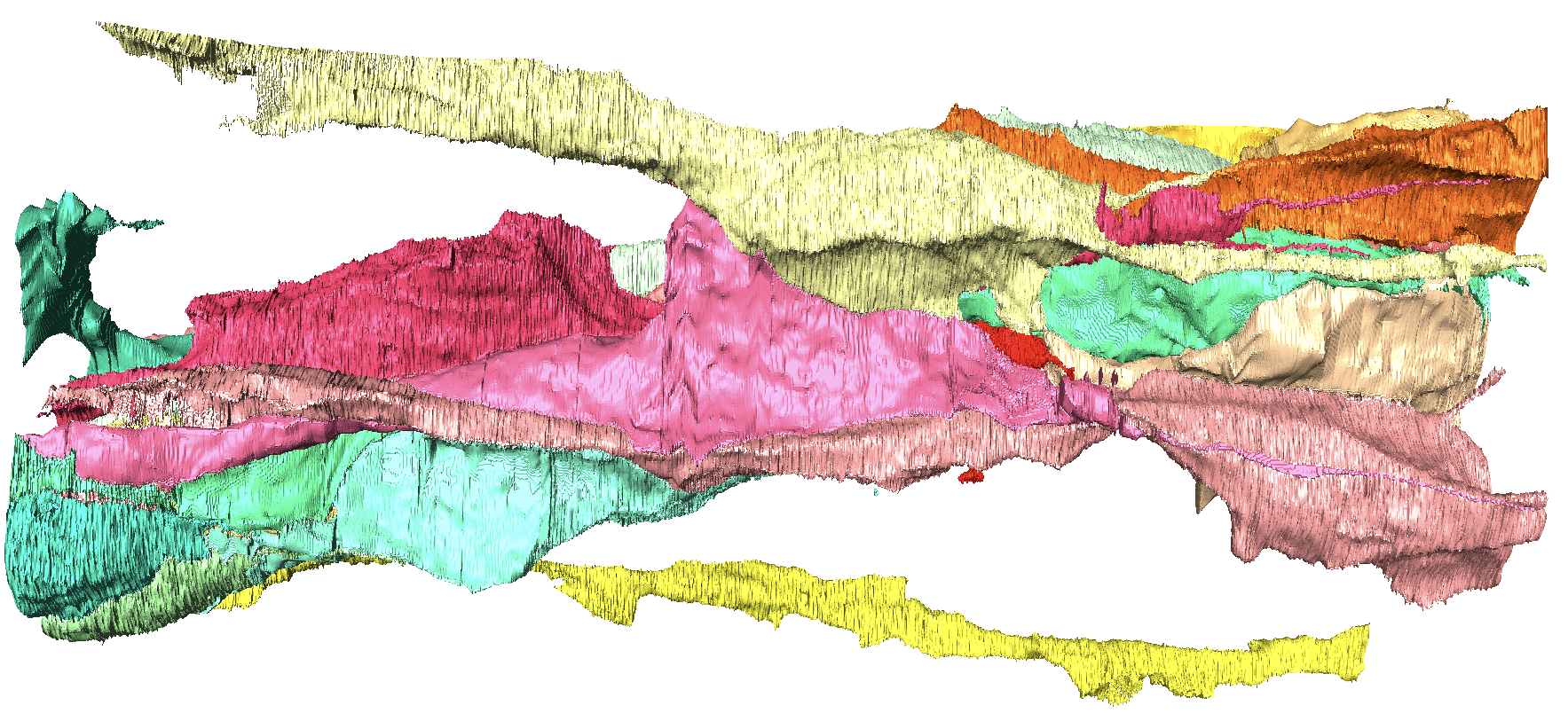

Supplement: Supplementary file 7 — Source data Fig. 2 [file 44321_2024_73_MOESM7_ESM.zip › Figure 2/2A/2A Neuronal chain in the adult RMS.tif]

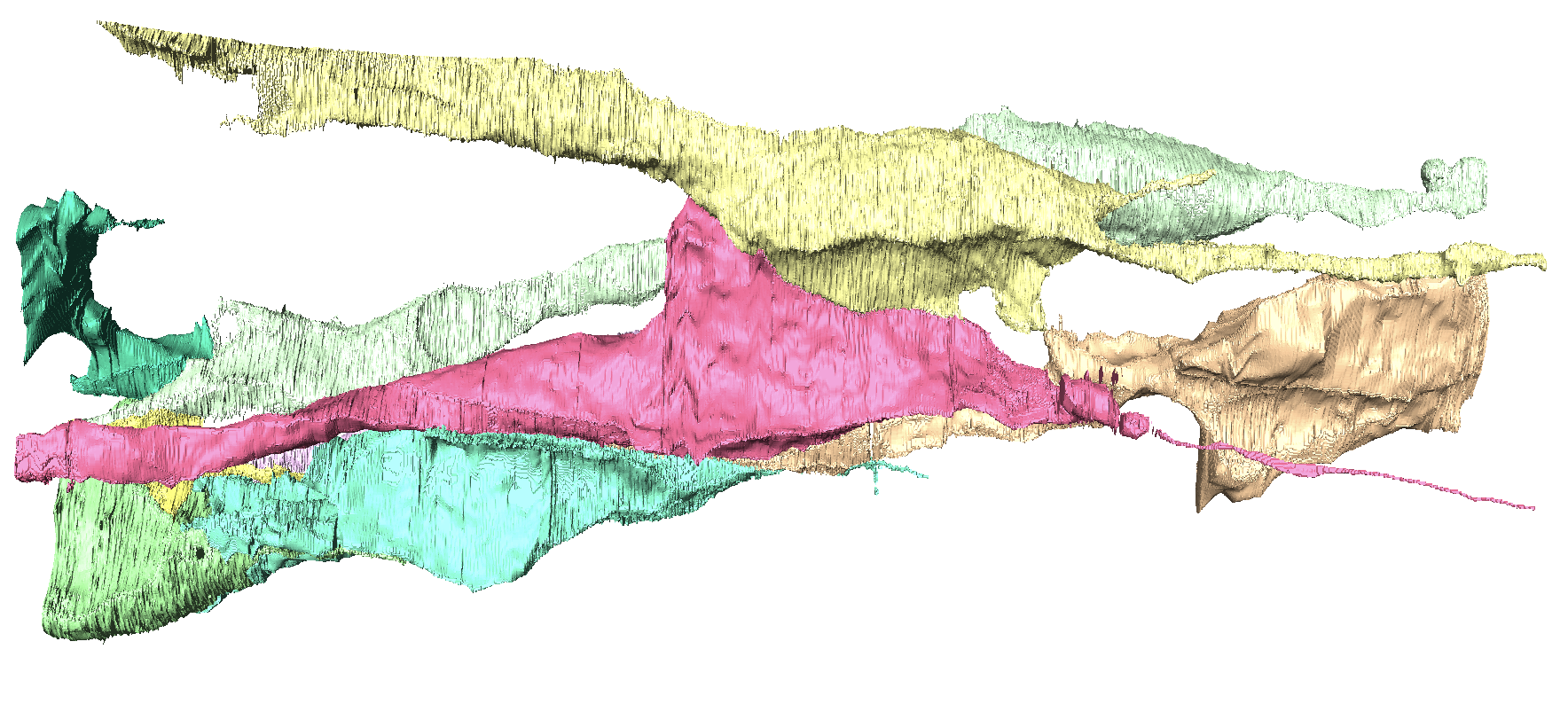

Supplement: Supplementary file 7 — Source data Fig. 2 [file 44321_2024_73_MOESM7_ESM.zip › Figure 2/2A/2A' New neurons oriented to the OB-2.tif]

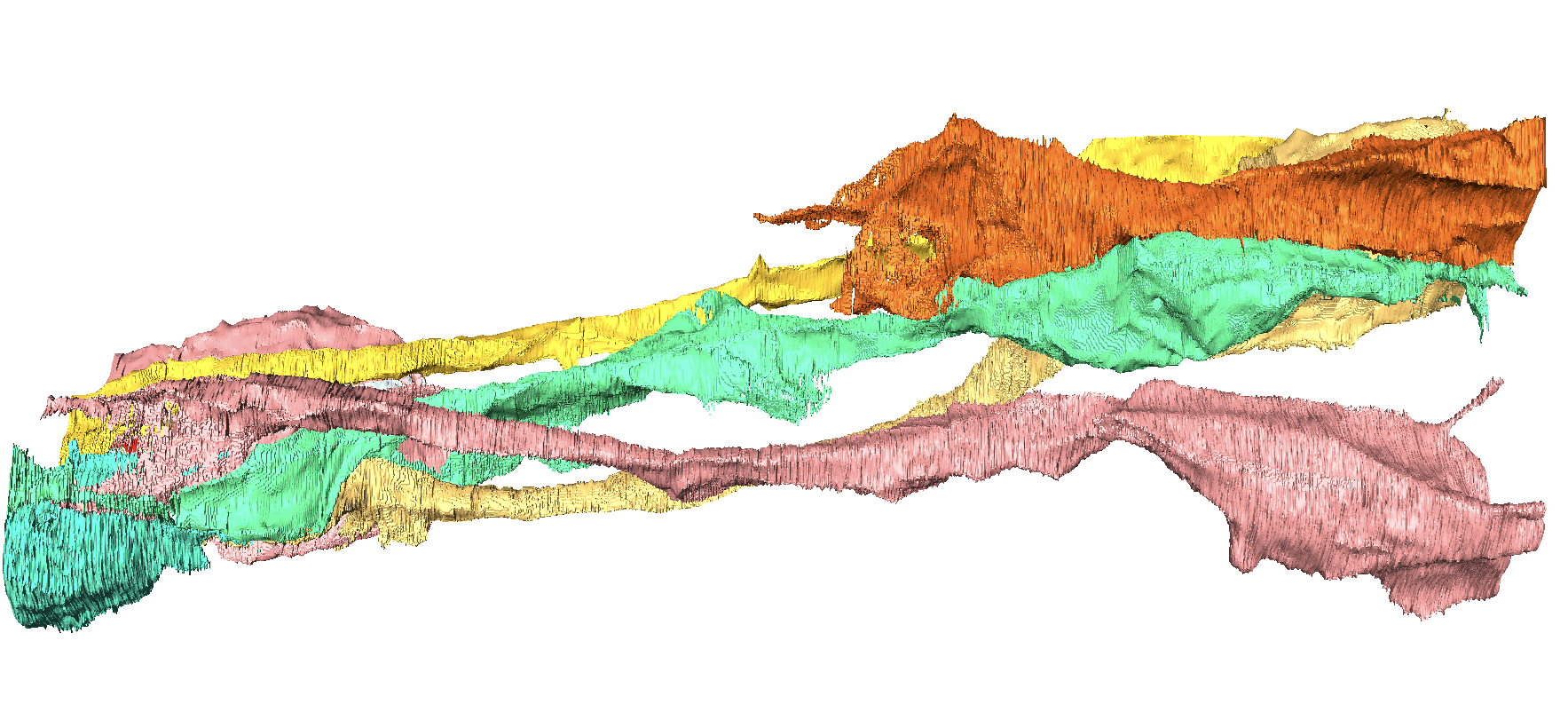

Supplement: Supplementary file 7 — Source data Fig. 2 [file 44321_2024_73_MOESM7_ESM.zip › Figure 2/2A/2A' New neurons oriented to the OB-1.tif]

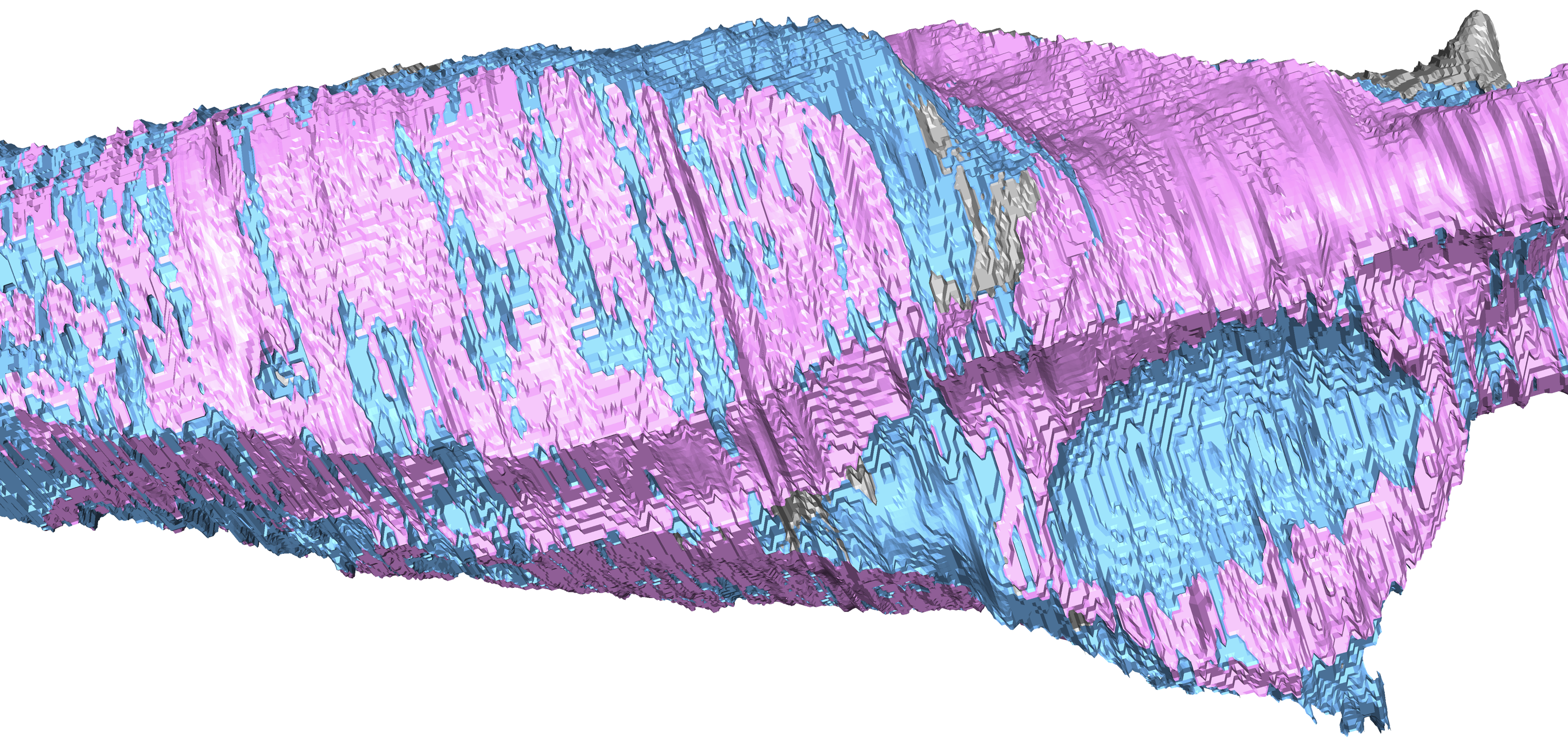

Supplement: Supplementary file 7 — Source data Fig. 2 [file 44321_2024_73_MOESM7_ESM.zip › Figure 2/2F/2F Normal RMS.tif]

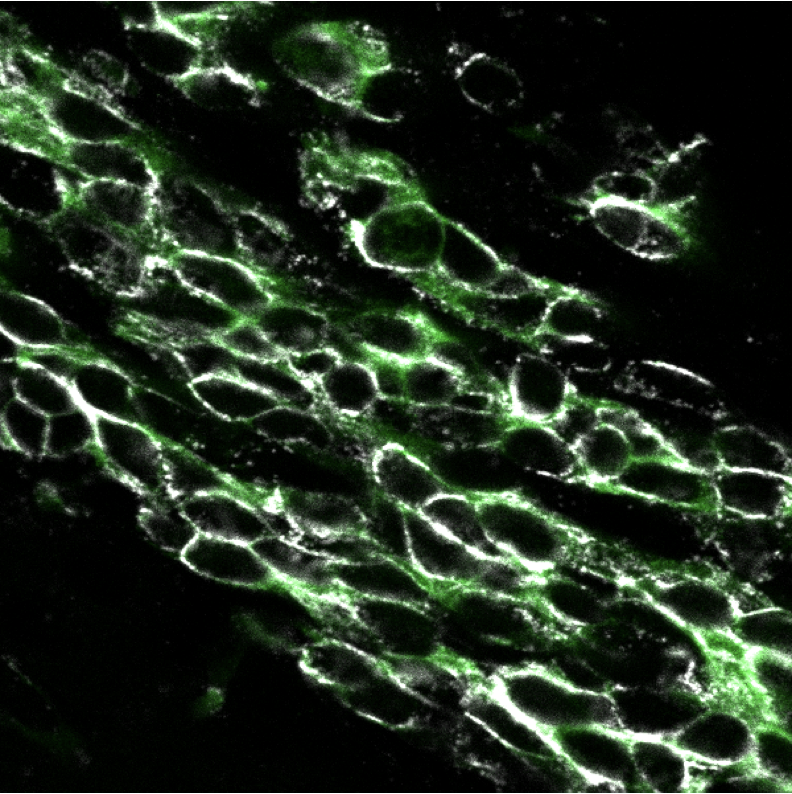

Supplement: Supplementary file 7 — Source data Fig. 2 [file 44321_2024_73_MOESM7_ESM.zip › Figure 2/2M/2M Normal RMS Dcx, PSA-NCAM.tif]

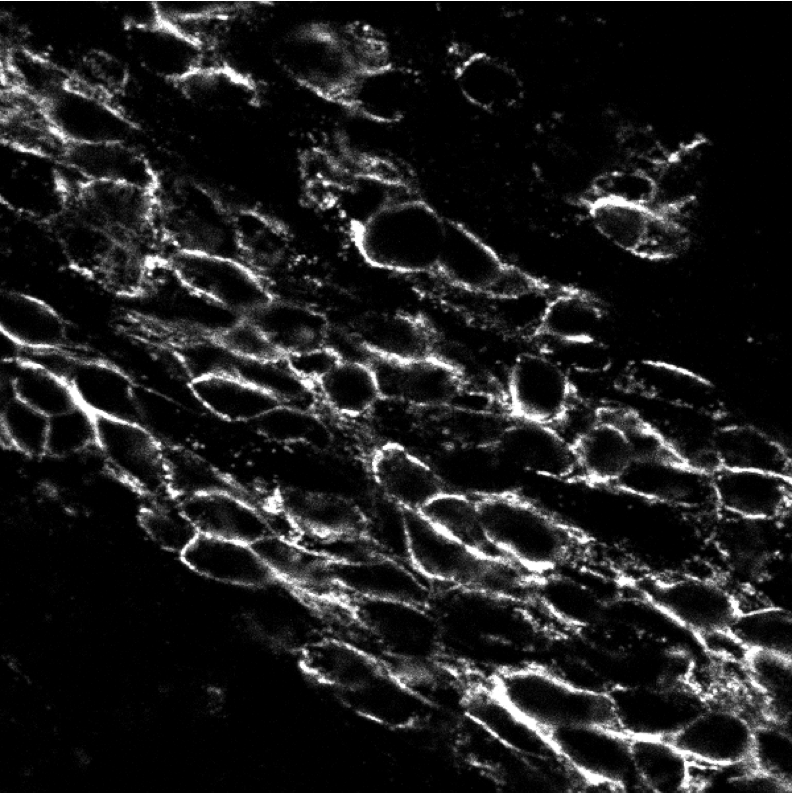

Supplement: Supplementary file 7 — Source data Fig. 2 [file 44321_2024_73_MOESM7_ESM.zip › Figure 2/2M/2M' Normal RMS PSA-NCAM.tif]

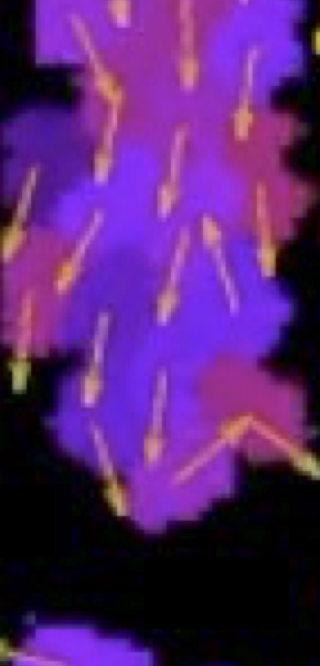

Supplement: Supplementary file 7 — Source data Fig. 2 [file 44321_2024_73_MOESM7_ESM.zip › Figure 2/2J/2J Appropriate adhesion-1.tif]

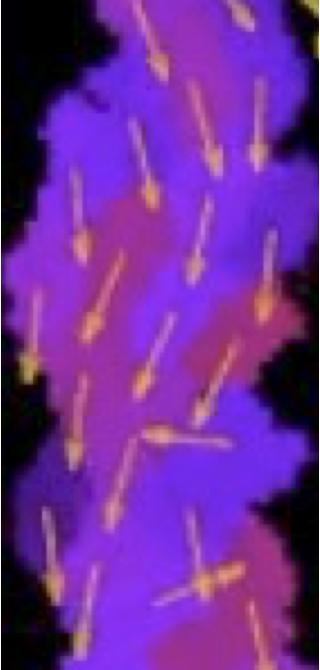

Supplement: Supplementary file 7 — Source data Fig. 2 [file 44321_2024_73_MOESM7_ESM.zip › Figure 2/2J/2J Appropriate adhesion-3.tif]

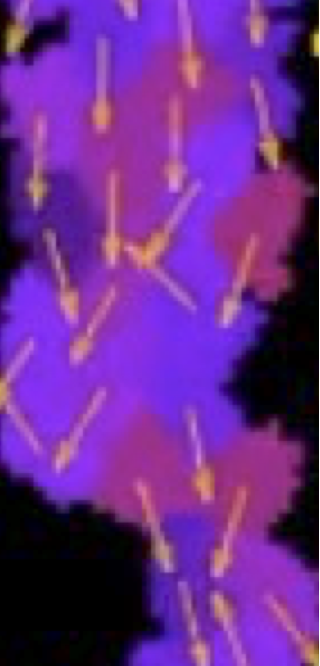

Supplement: Supplementary file 7 — Source data Fig. 2 [file 44321_2024_73_MOESM7_ESM.zip › Figure 2/2J/2J Appropriate adhesion-2.tif]

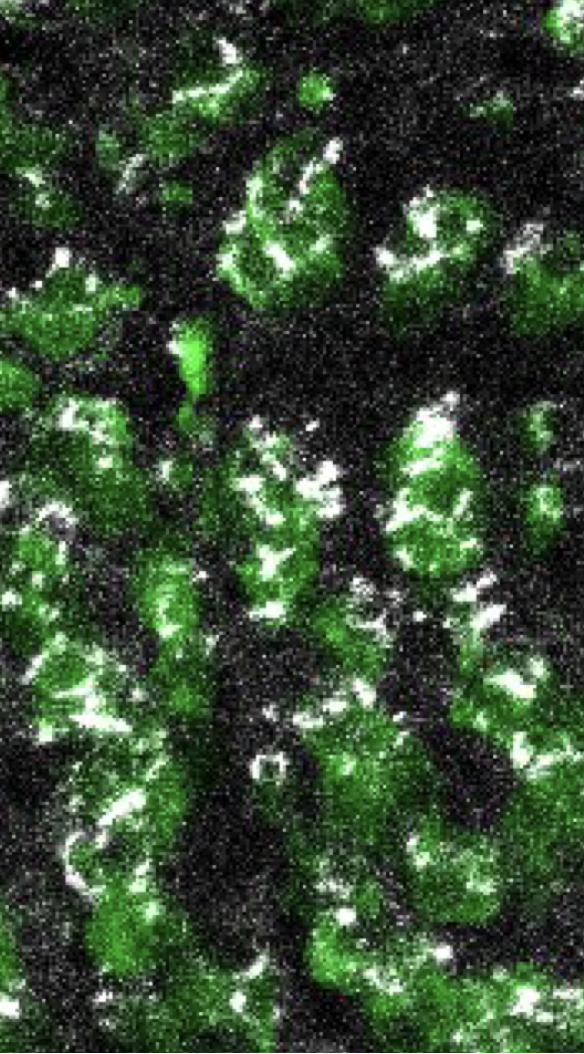

Supplement: Supplementary file 7 — Source data Fig. 2 [file 44321_2024_73_MOESM7_ESM.zip › Figure 2/2Q/2Q ctrl Dcx-GFP, PSA-NCAM.tif]

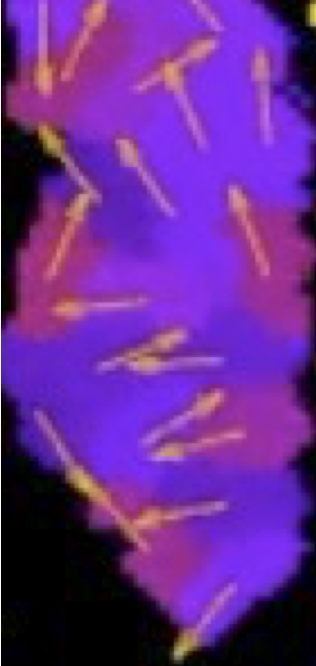

Supplement: Supplementary file 7 — Source data Fig. 2 [file 44321_2024_73_MOESM7_ESM.zip › Figure 2/2K/2K Excessive adhesion-3.tif]

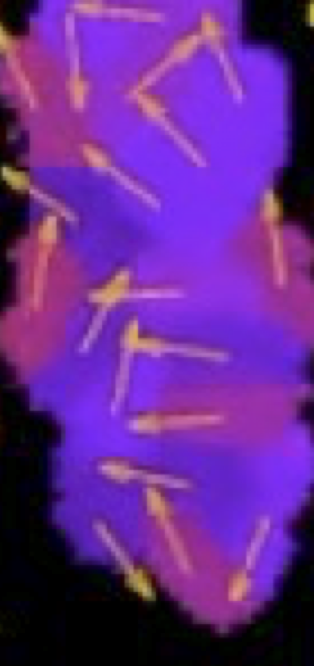

Supplement: Supplementary file 7 — Source data Fig. 2 [file 44321_2024_73_MOESM7_ESM.zip › Figure 2/2K/2K Excessive adhesion-2.tif]

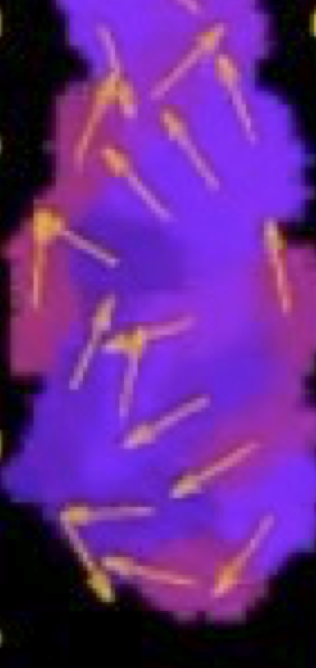

Supplement: Supplementary file 7 — Source data Fig. 2 [file 44321_2024_73_MOESM7_ESM.zip › Figure 2/2K/2K Excessive adhesion-1.tif]

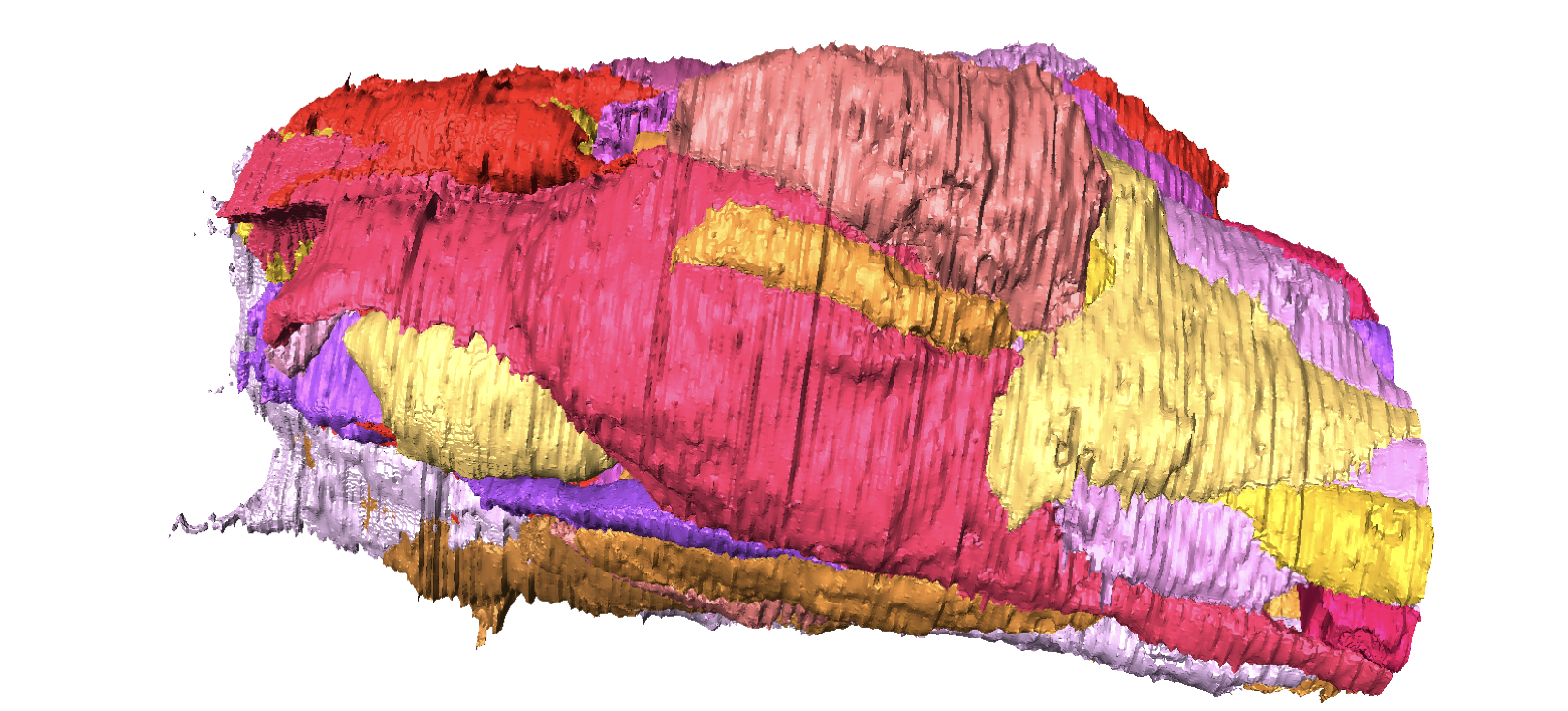

Supplement: Supplementary file 7 — Source data Fig. 2 [file 44321_2024_73_MOESM7_ESM.zip › Figure 2/2B/2B Neuronal chain in the injured site.tif]

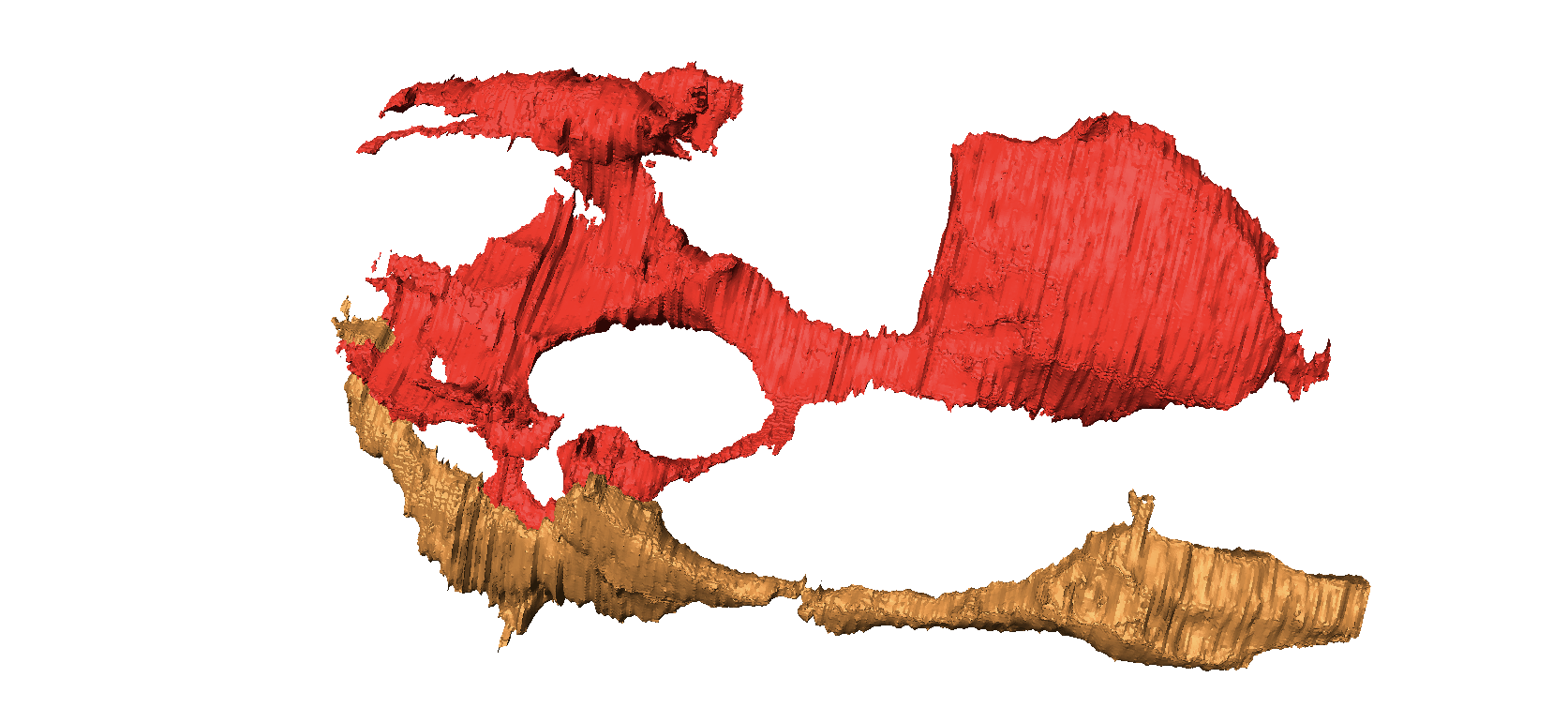

Supplement: Supplementary file 7 — Source data Fig. 2 [file 44321_2024_73_MOESM7_ESM.zip › Figure 2/2B/2B' New neurons oriented to the injured site-1.tif]

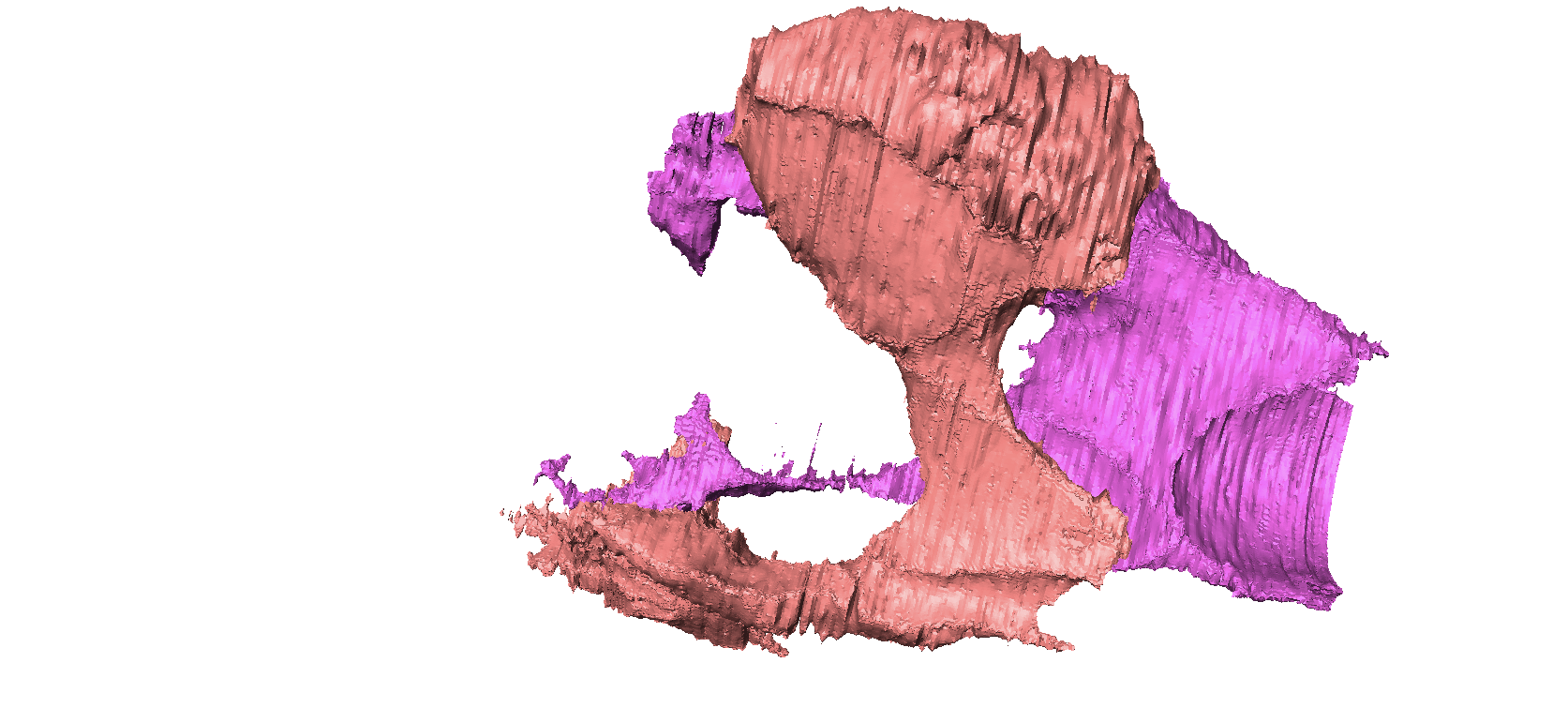

Supplement: Supplementary file 7 — Source data Fig. 2 [file 44321_2024_73_MOESM7_ESM.zip › Figure 2/2B/2B' New neurons oriented to the injured site-2.tif]

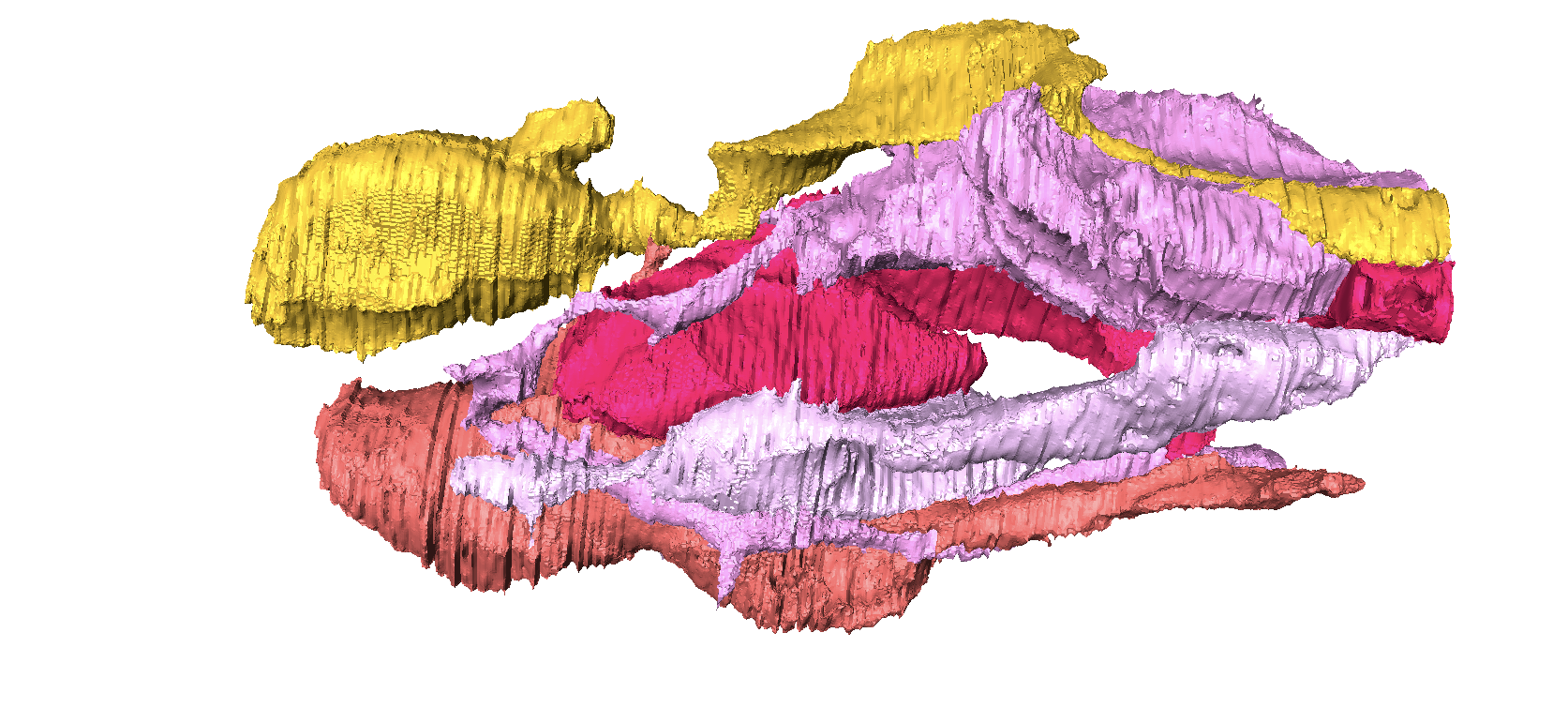

Supplement: Supplementary file 7 — Source data Fig. 2 [file 44321_2024_73_MOESM7_ESM.zip › Figure 2/2B/2B'' New neuron oriented in the reverse direction-2.tif]

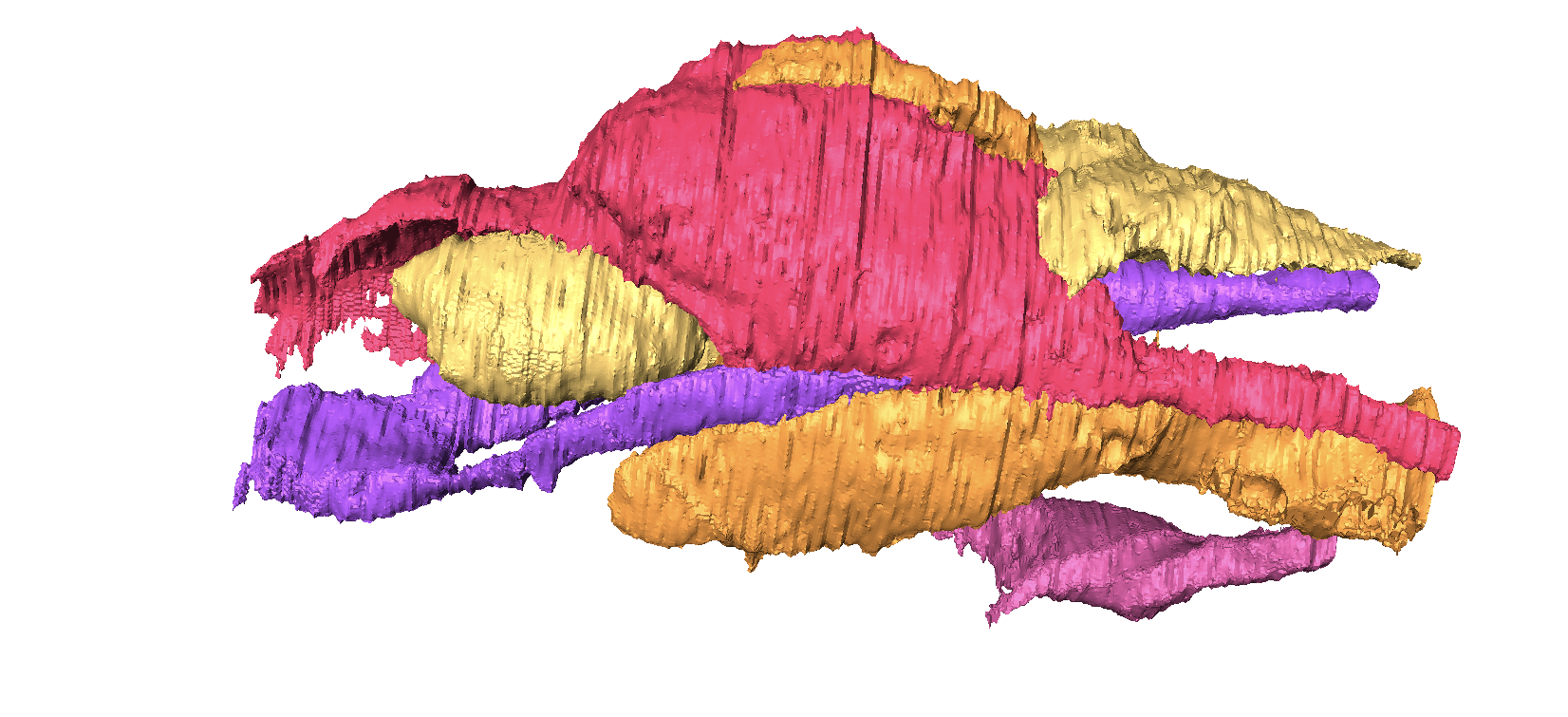

Supplement: Supplementary file 7 — Source data Fig. 2 [file 44321_2024_73_MOESM7_ESM.zip › Figure 2/2B/2B'' New neuron oriented in the reverse direction-1.tif]

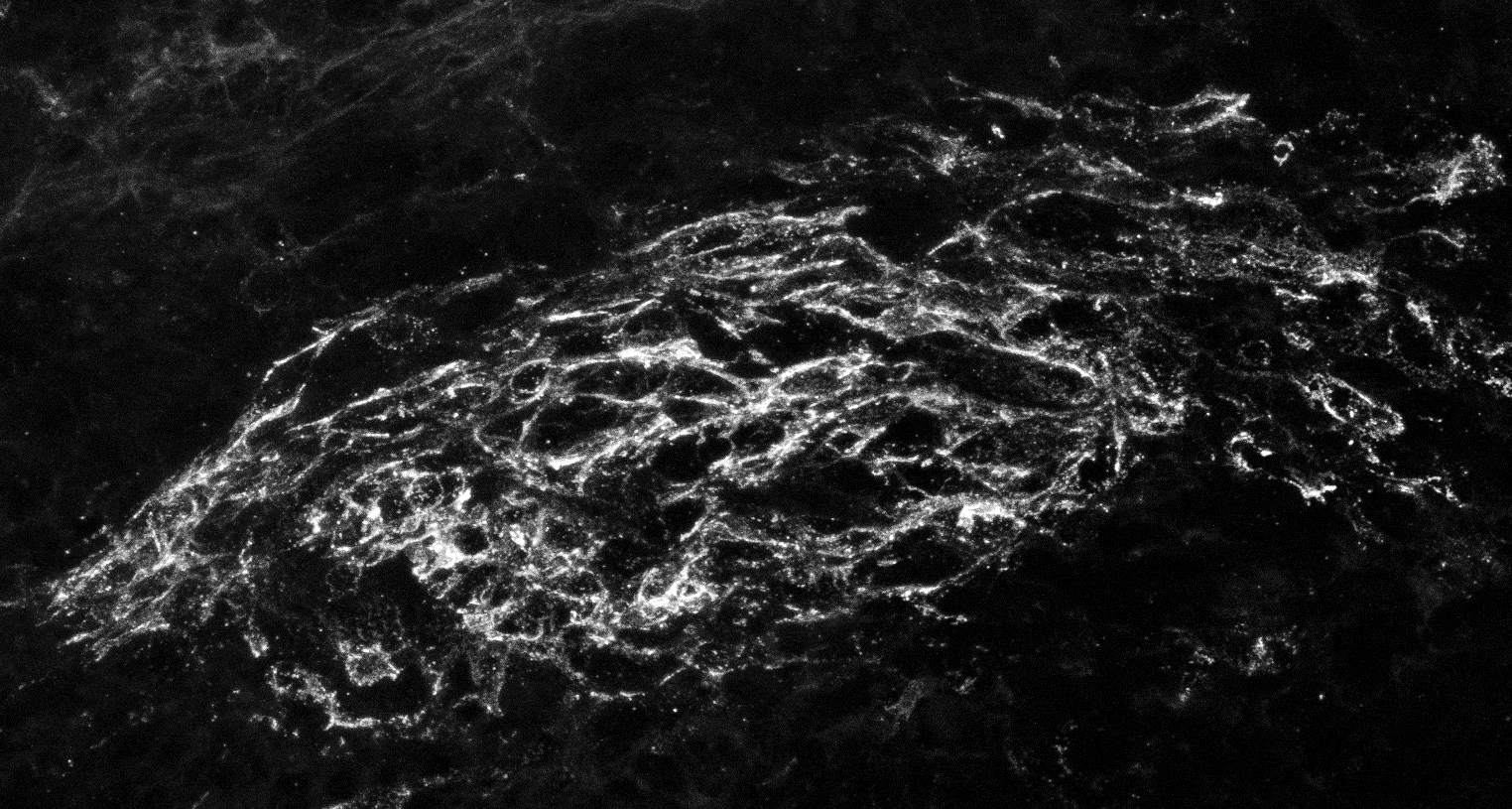

Supplement: Supplementary file 8 — Source data Fig. 3 [file 44321_2024_73_MOESM8_ESM.zip › Figure 3/3J/3J' Neu1-KD PSA-NCAM-injured site.tif]

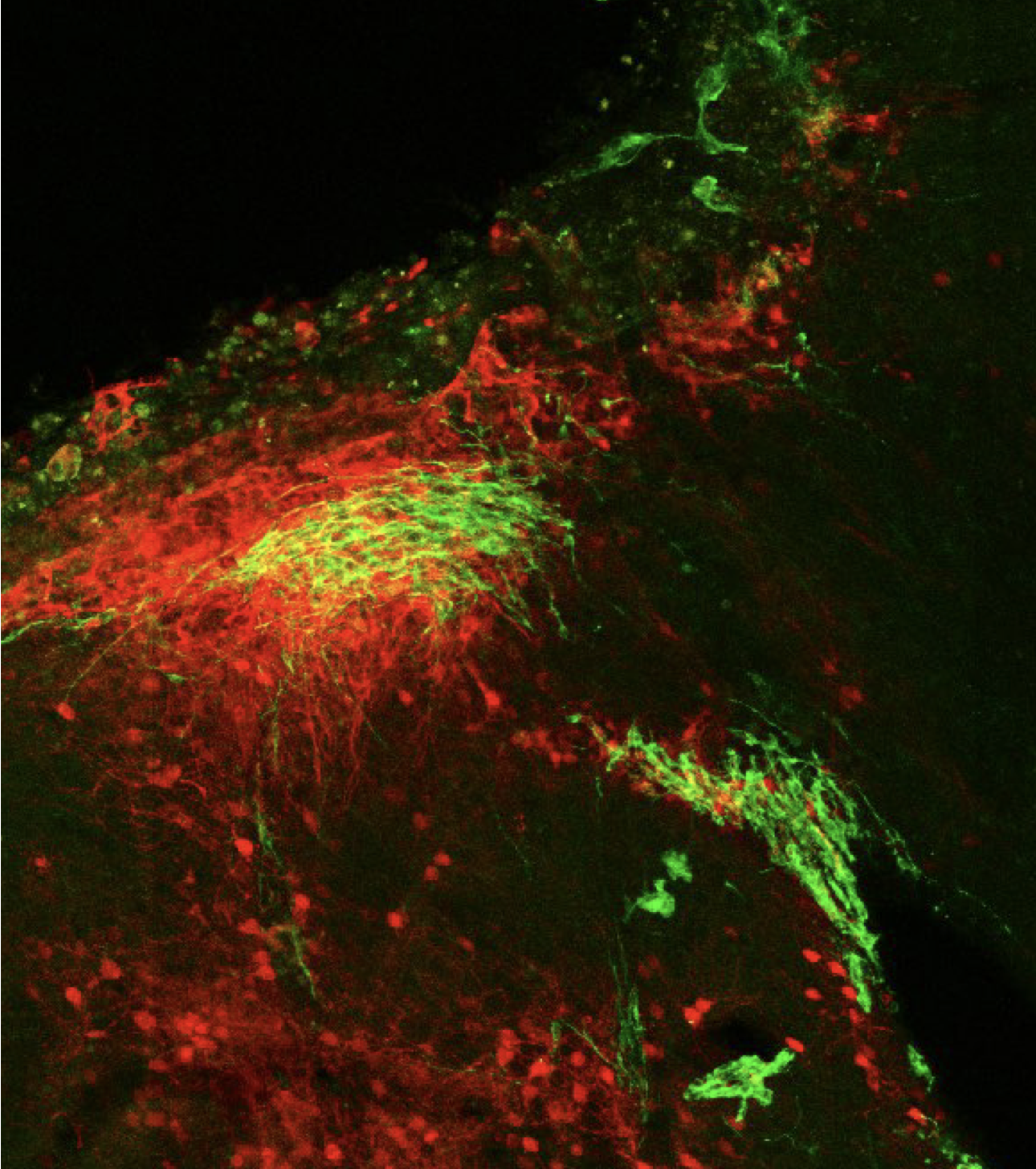

Supplement: Supplementary file 8 — Source data Fig. 3 [file 44321_2024_73_MOESM8_ESM.zip › Figure 3/3J/3J Neu1-KD Dcx, DsRed.tif]

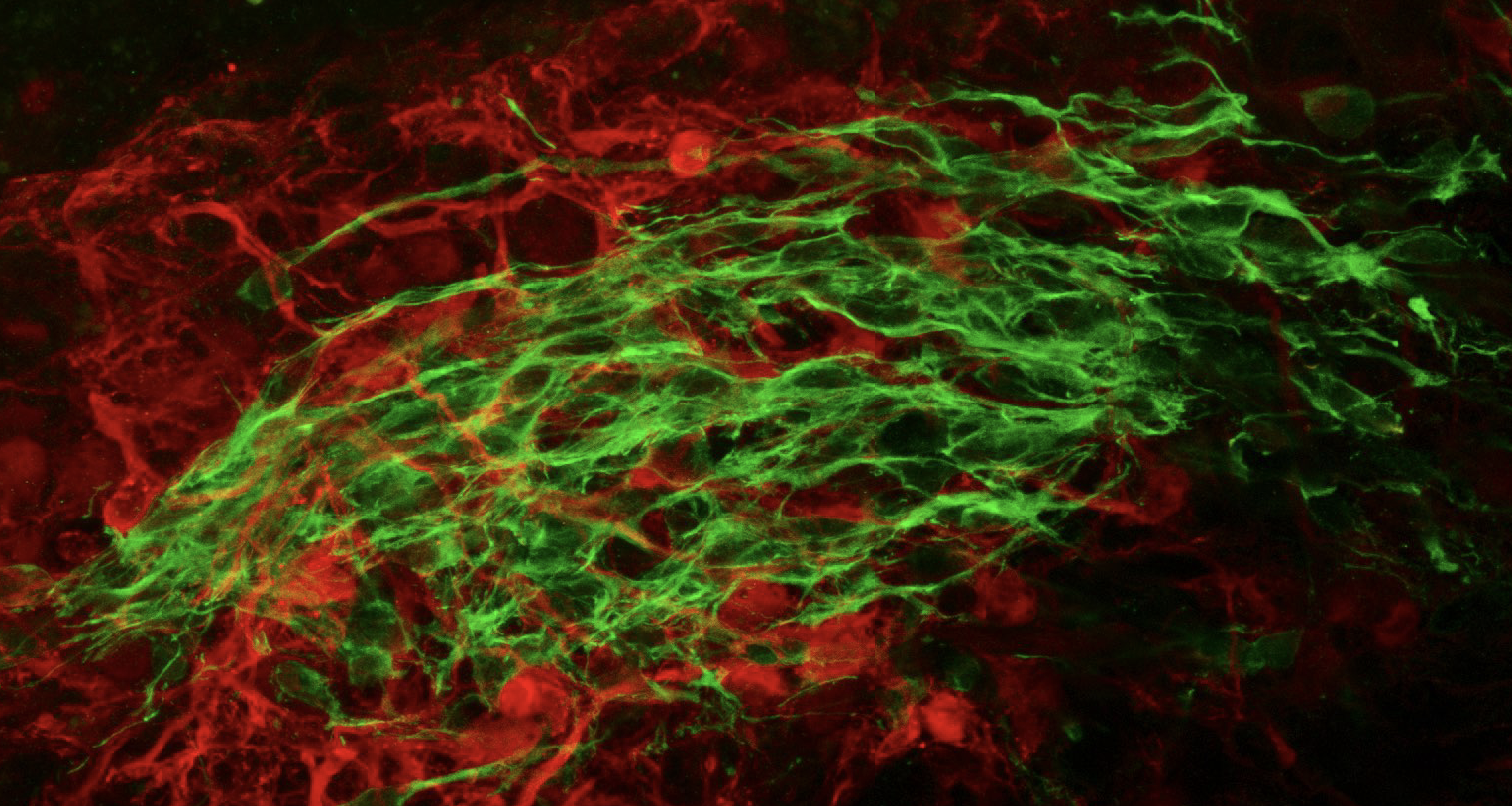

Supplement: Supplementary file 8 — Source data Fig. 3 [file 44321_2024_73_MOESM8_ESM.zip › Figure 3/3J/3J' Neu1-KD Dcx, DsRed-injured site.tif]

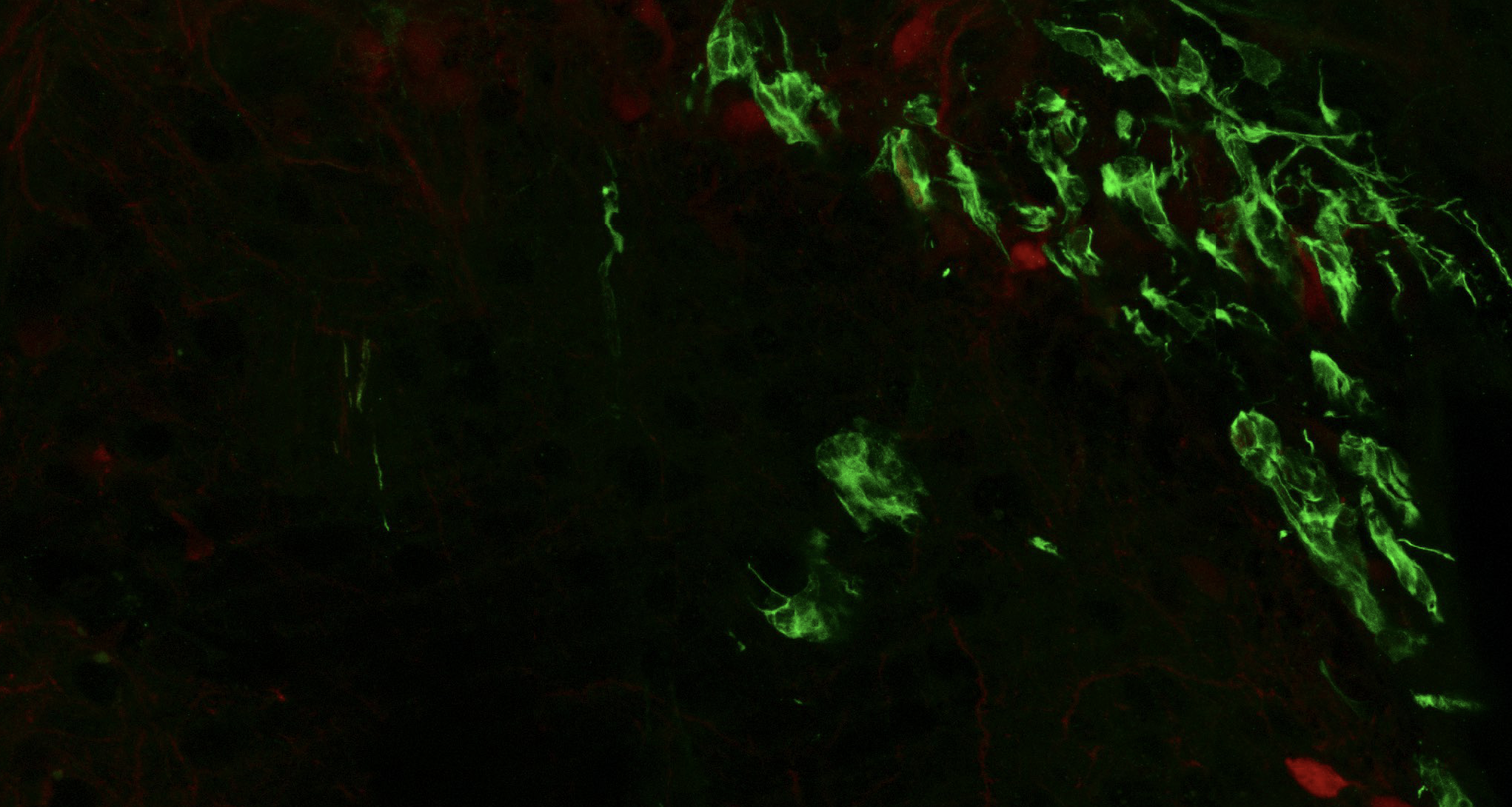

Supplement: Supplementary file 8 — Source data Fig. 3 [file 44321_2024_73_MOESM8_ESM.zip › Figure 3/3J/3J'' Neu1-KD Dcx, DsRed-V-SVZ, St.tif]

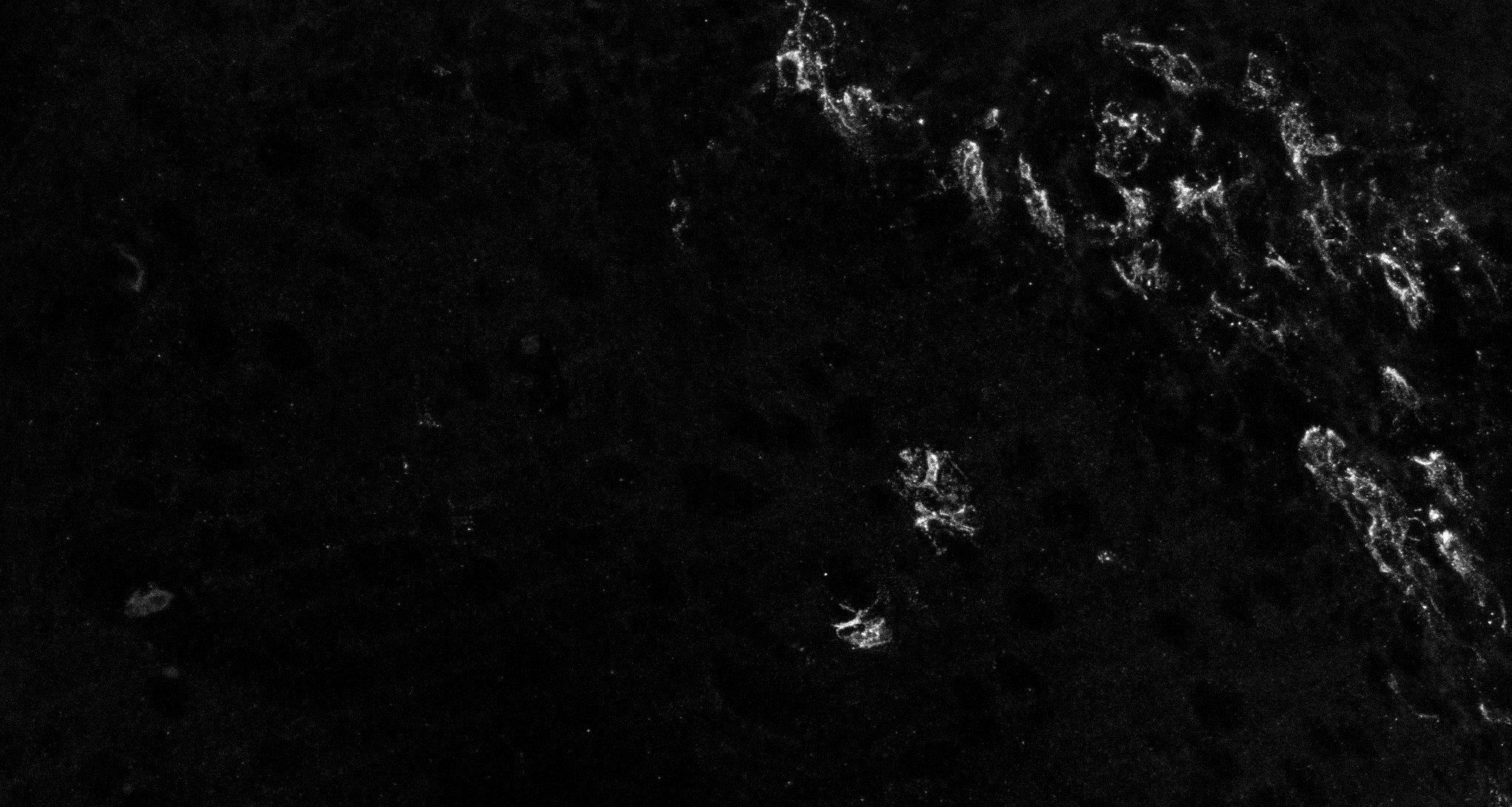

Supplement: Supplementary file 8 — Source data Fig. 3 [file 44321_2024_73_MOESM8_ESM.zip › Figure 3/3J/3J'' Neu1-KD PSA-NCAM-V-SVZ, St.tif]

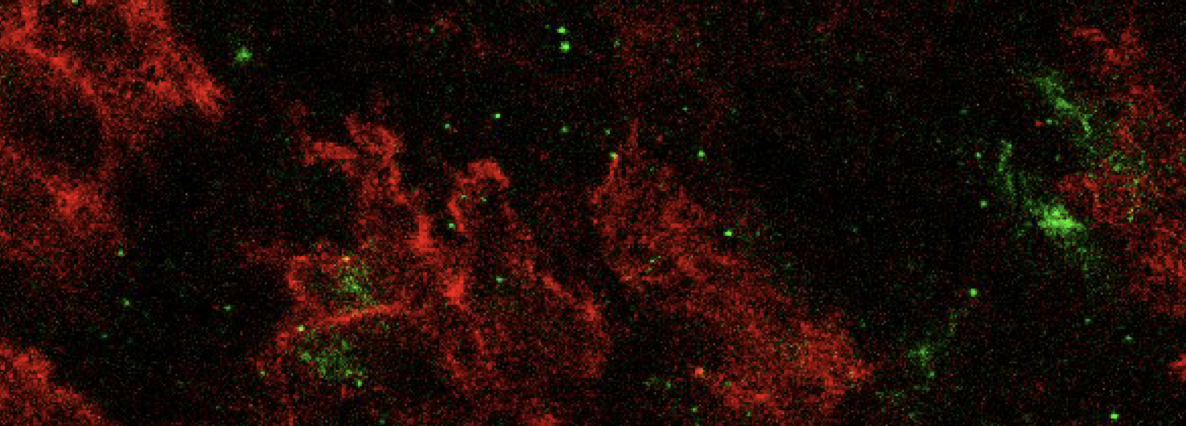

Supplement: Supplementary file 8 — Source data Fig. 3 [file 44321_2024_73_MOESM8_ESM.zip › Figure 3/3F/3F' Injured brain Neu1, GFAP.tif]

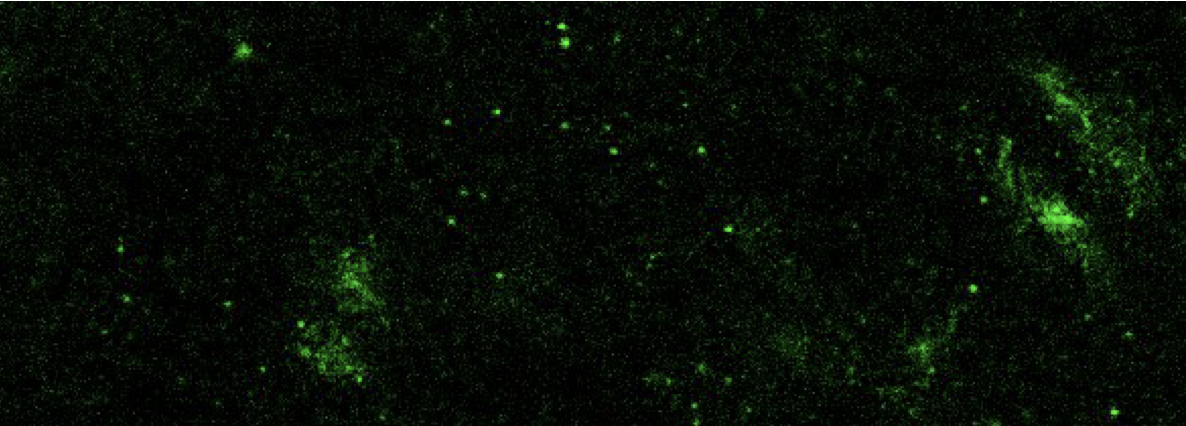

Supplement: Supplementary file 8 — Source data Fig. 3 [file 44321_2024_73_MOESM8_ESM.zip › Figure 3/3F/3F Injured brain Neu1, Iba1, GFAP.tif]

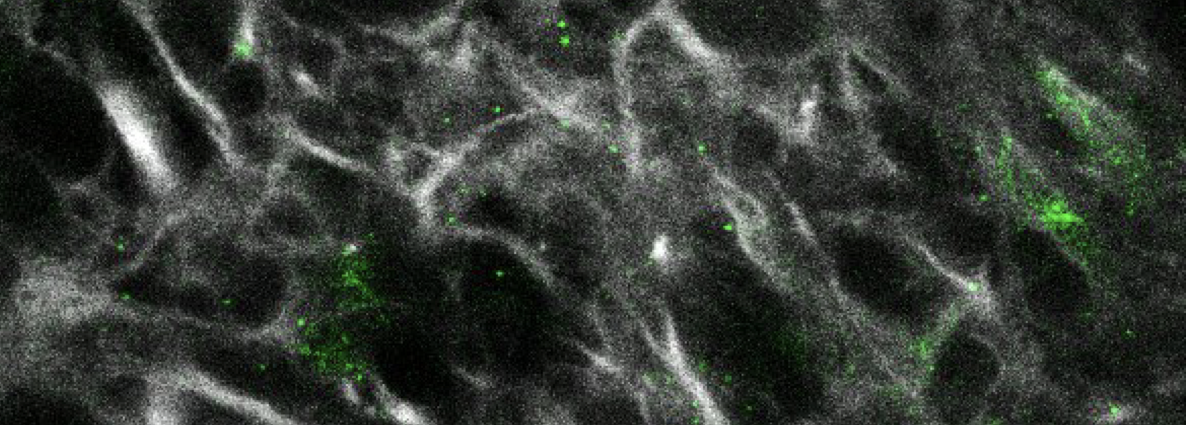

Supplement: Supplementary file 8 — Source data Fig. 3 [file 44321_2024_73_MOESM8_ESM.zip › Figure 3/3F/3F'' Injured brain Neu1, Iba1.tif]

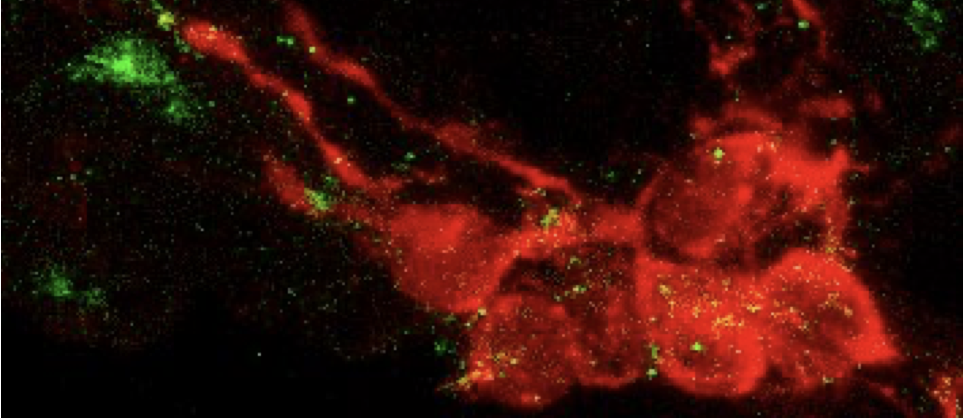

Supplement: Supplementary file 8 — Source data Fig. 3 [file 44321_2024_73_MOESM8_ESM.zip › Figure 3/3G/3G' Injured brain Neu1, Dcx.tif]

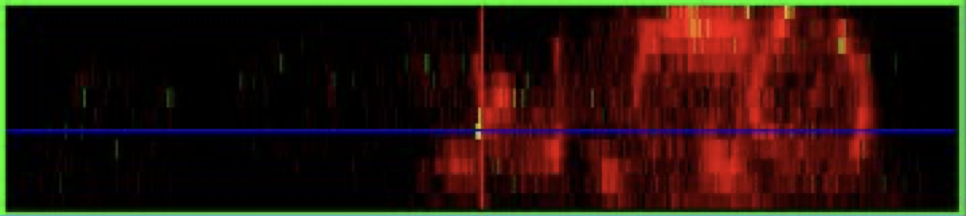

Supplement: Supplementary file 8 — Source data Fig. 3 [file 44321_2024_73_MOESM8_ESM.zip › Figure 3/3G/3G'' Injured brain Neu1, Dcx-2.tif]

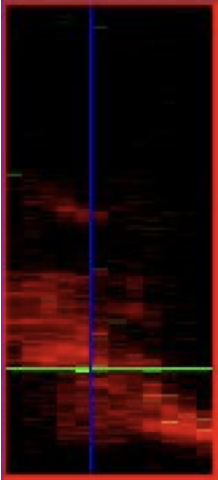

Supplement: Supplementary file 8 — Source data Fig. 3 [file 44321_2024_73_MOESM8_ESM.zip › Figure 3/3G/3G'' Injured brain Neu1, Dcx-3.tif]

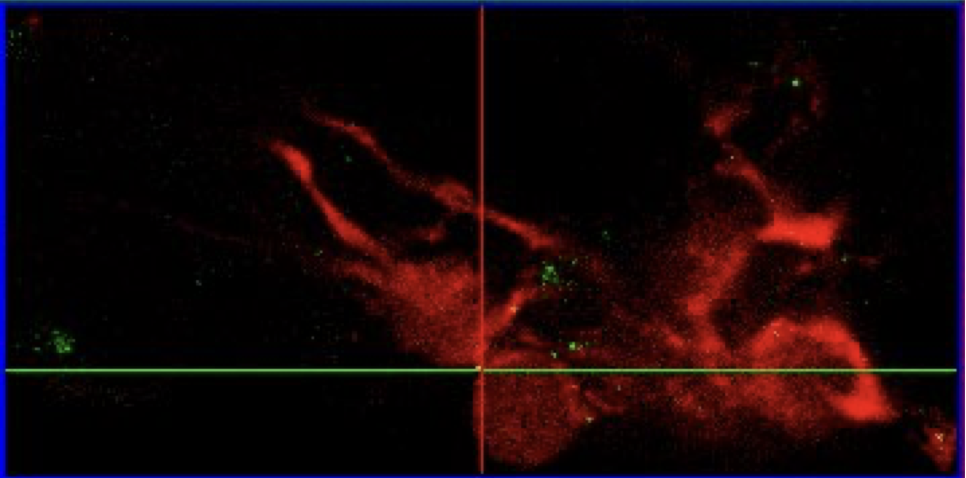

Supplement: Supplementary file 8 — Source data Fig. 3 [file 44321_2024_73_MOESM8_ESM.zip › Figure 3/3G/3G'' Injured brain Neu1, Dcx-1.tif]

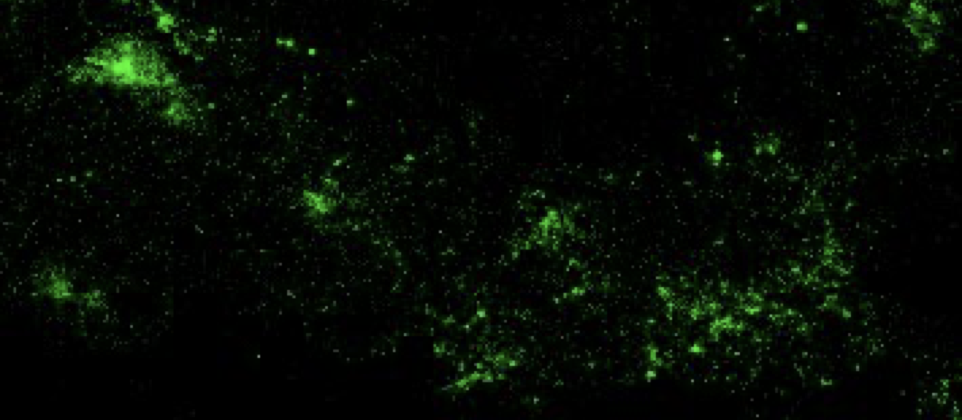

Supplement: Supplementary file 8 — Source data Fig. 3 [file 44321_2024_73_MOESM8_ESM.zip › Figure 3/3G/3G Injured brain Neu1.tif]

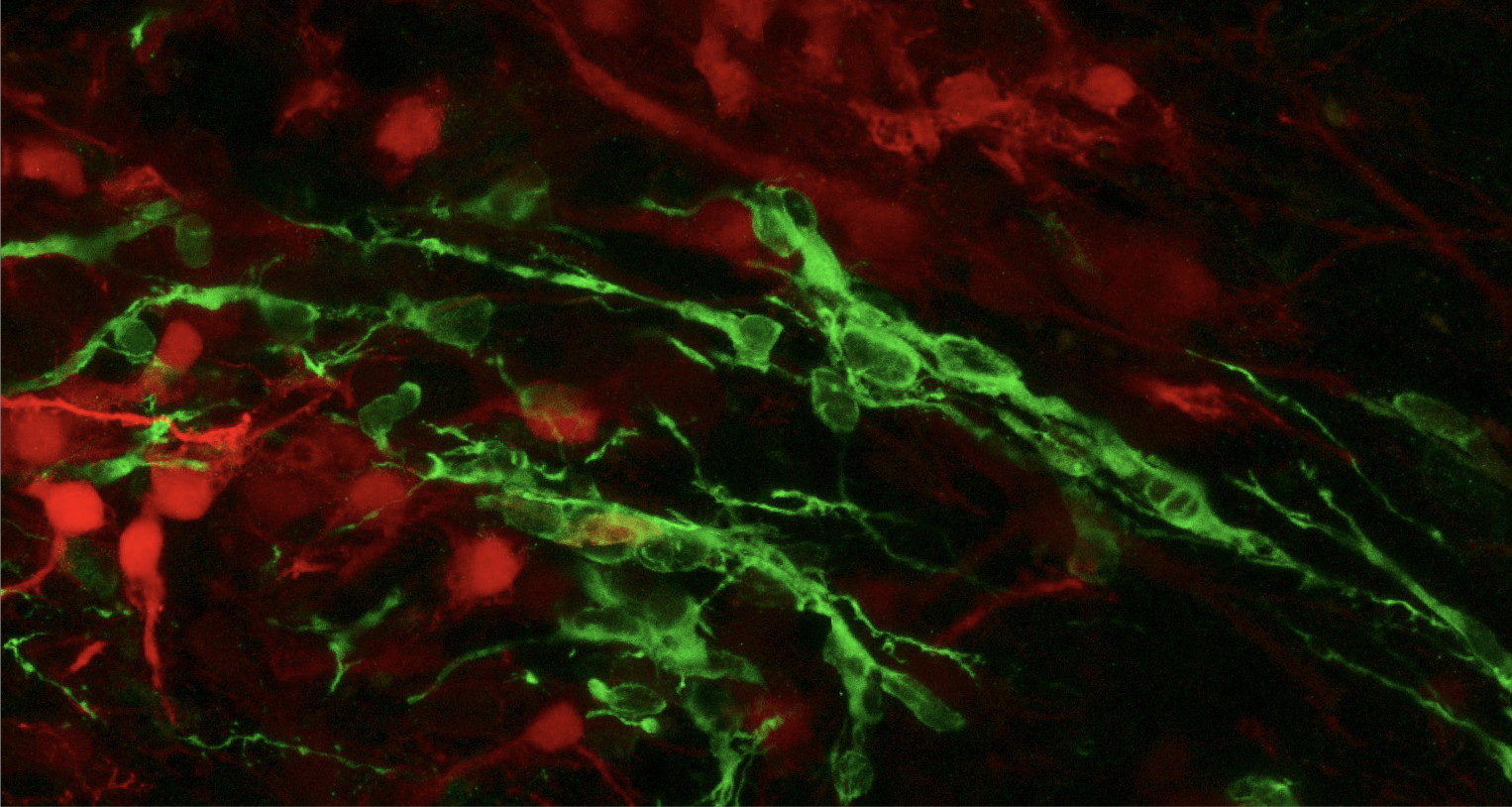

Supplement: Supplementary file 8 — Source data Fig. 3 [file 44321_2024_73_MOESM8_ESM.zip › Figure 3/3I/3I' ctrl-KD Dcx, DsRed-injured site.tif]

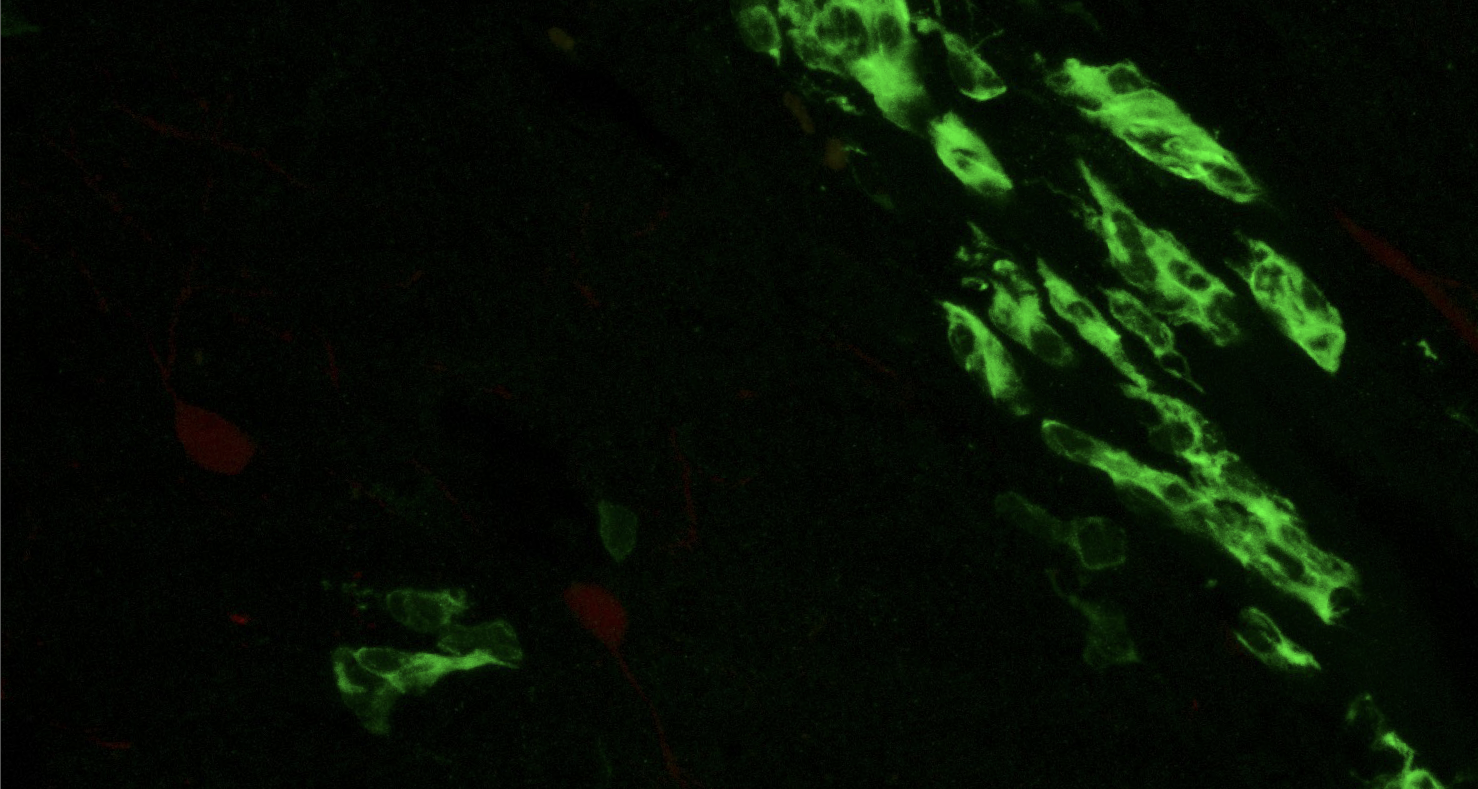

Supplement: Supplementary file 8 — Source data Fig. 3 [file 44321_2024_73_MOESM8_ESM.zip › Figure 3/3I/3I'' ctrl-KD Dcx, DsRed-V-SVZ, St.tif]

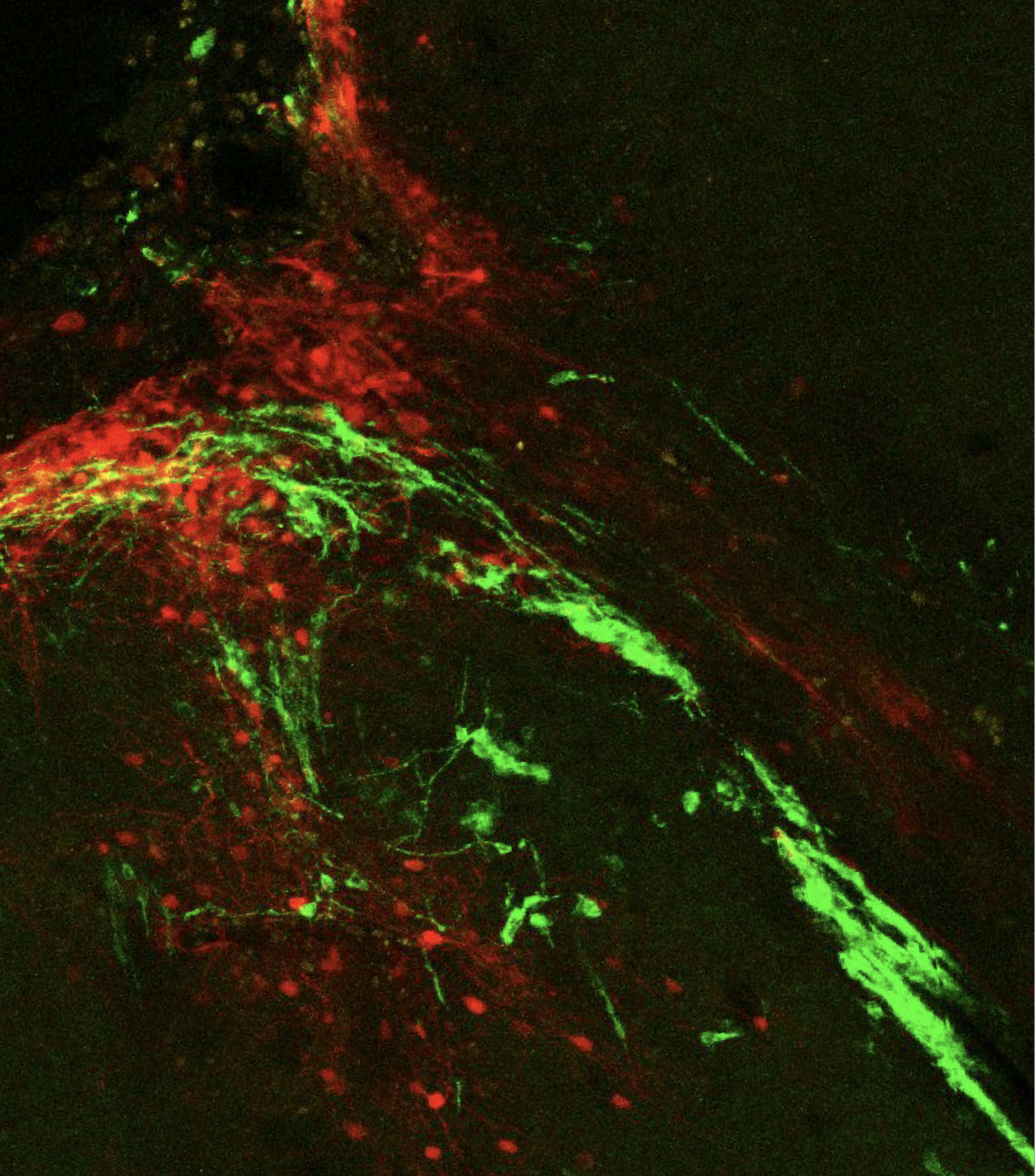

Supplement: Supplementary file 8 — Source data Fig. 3 [file 44321_2024_73_MOESM8_ESM.zip › Figure 3/3I/3I ctrl-KD Dcx, DsRed.tif]

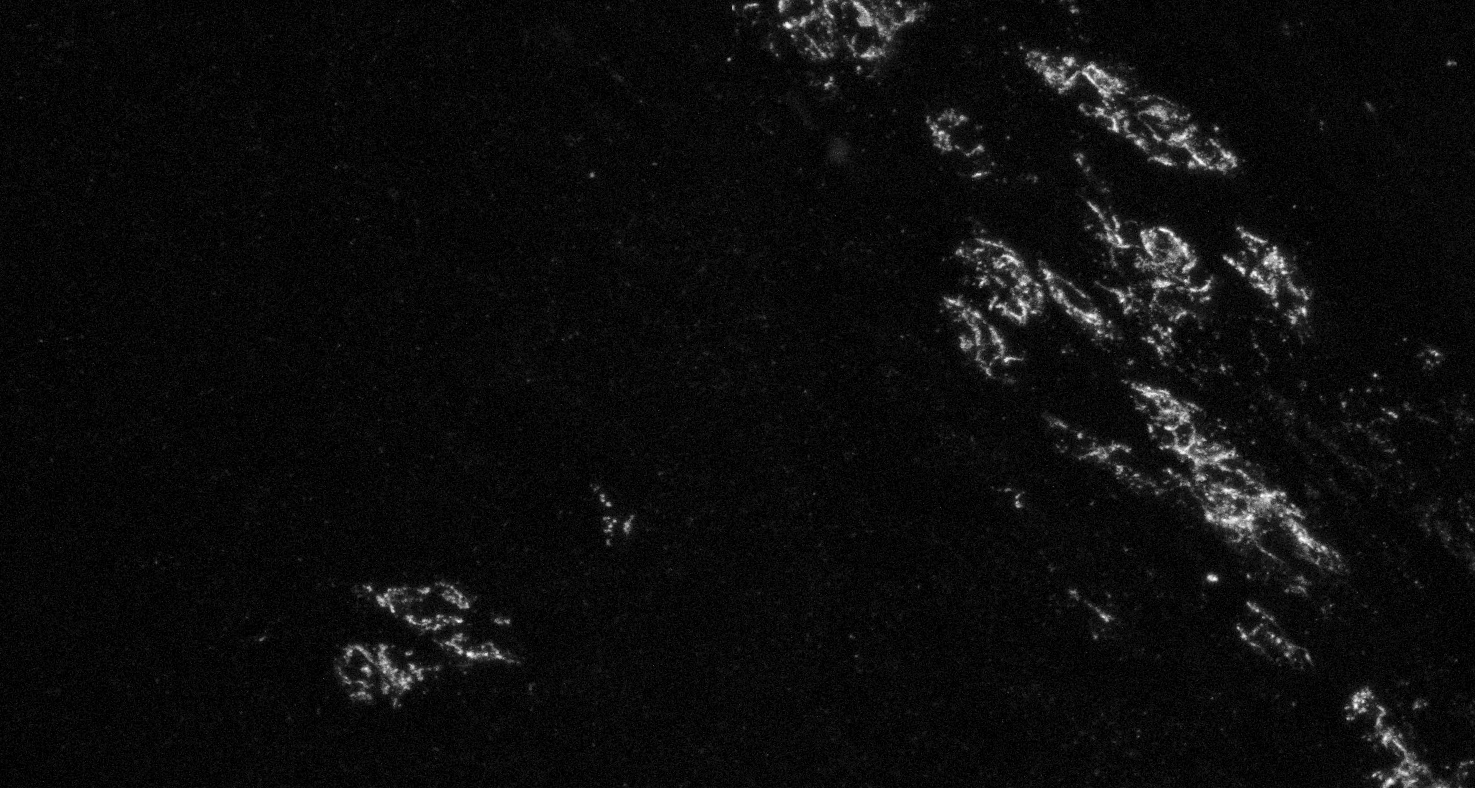

Supplement: Supplementary file 8 — Source data Fig. 3 [file 44321_2024_73_MOESM8_ESM.zip › Figure 3/3I/3I'' ctrl-KD PSA-NCAM-V-SVZ, St.tif]

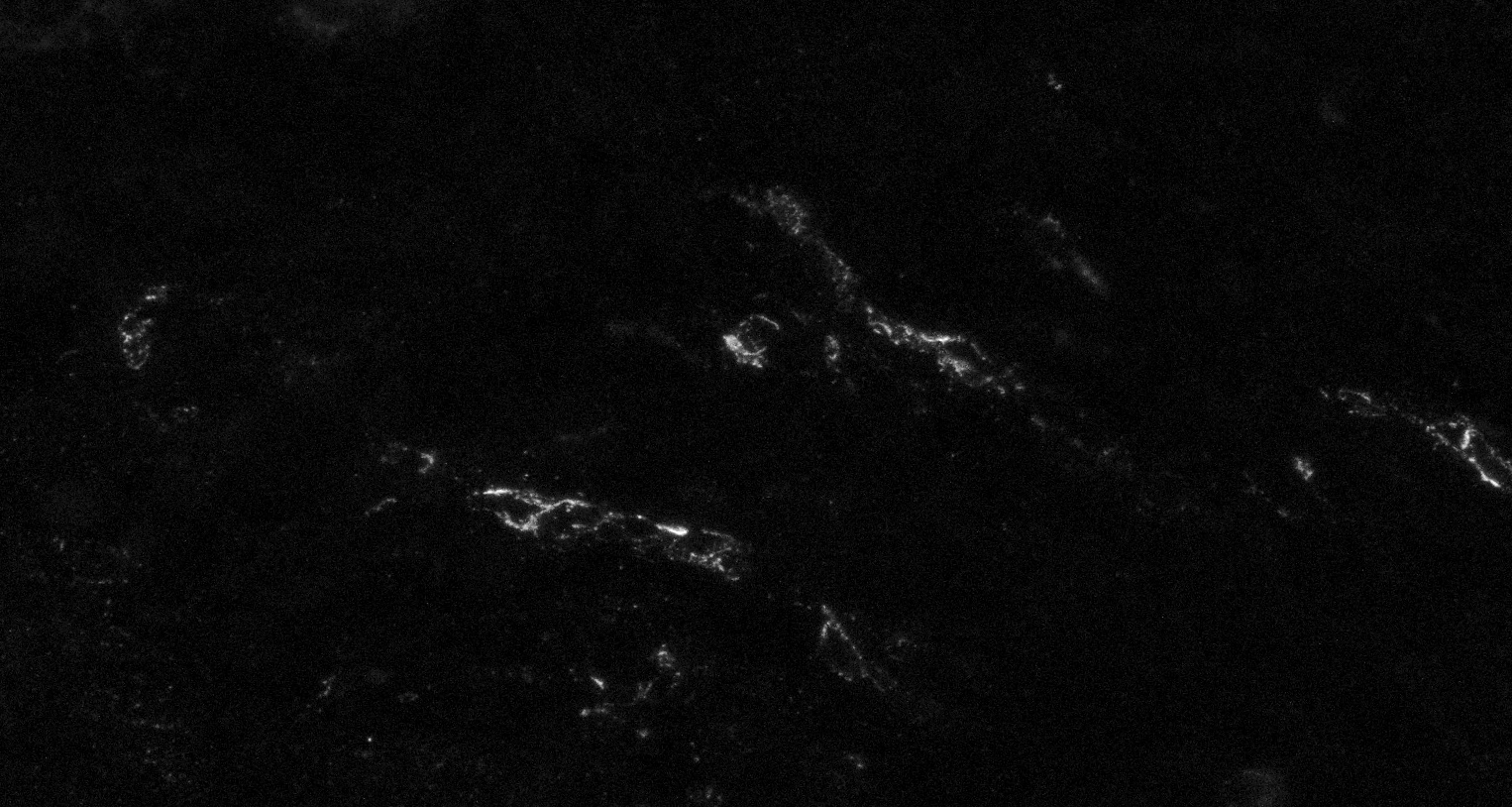

Supplement: Supplementary file 8 — Source data Fig. 3 [file 44321_2024_73_MOESM8_ESM.zip › Figure 3/3I/3I' ctrl-KD PSA-NCAM-injured site.tif]

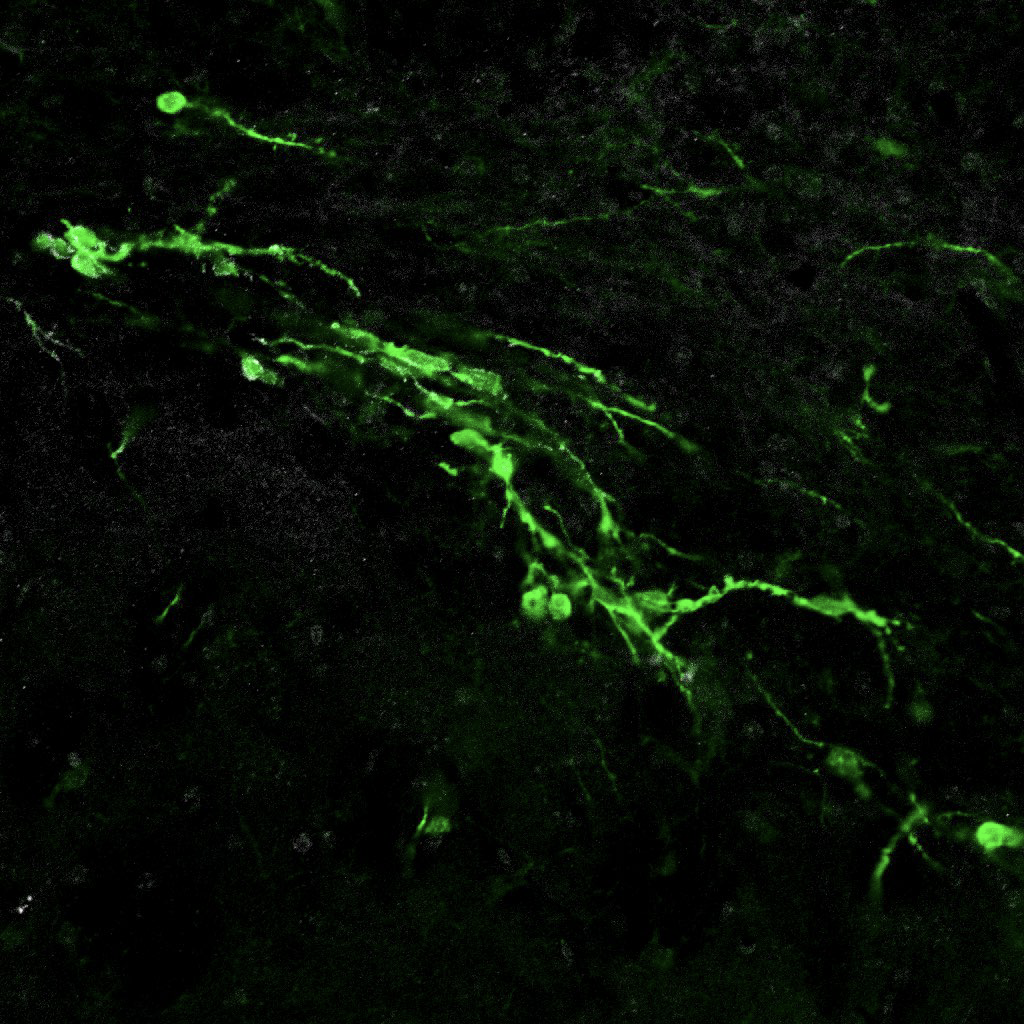

Supplement: Supplementary file 9 — Source data Fig. 4 [file 44321_2024_73_MOESM9_ESM.zip › Figure 4/4B/4B Ctrl Dcx, PSA-NCAM.tif]

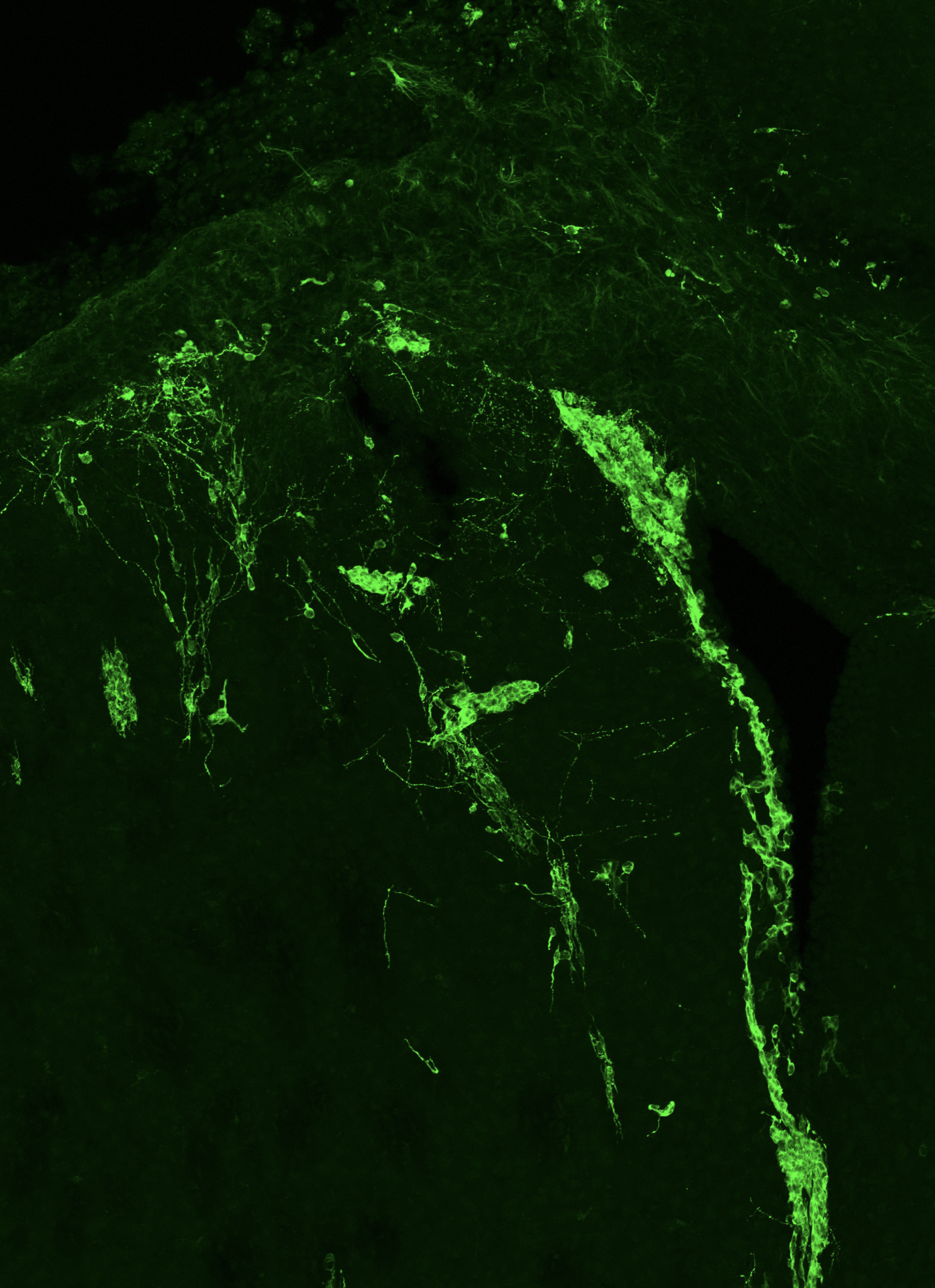

Supplement: Supplementary file 9 — Source data Fig. 4 [file 44321_2024_73_MOESM9_ESM.zip › Figure 4/4B/4B Ctrl Dcx.tif]

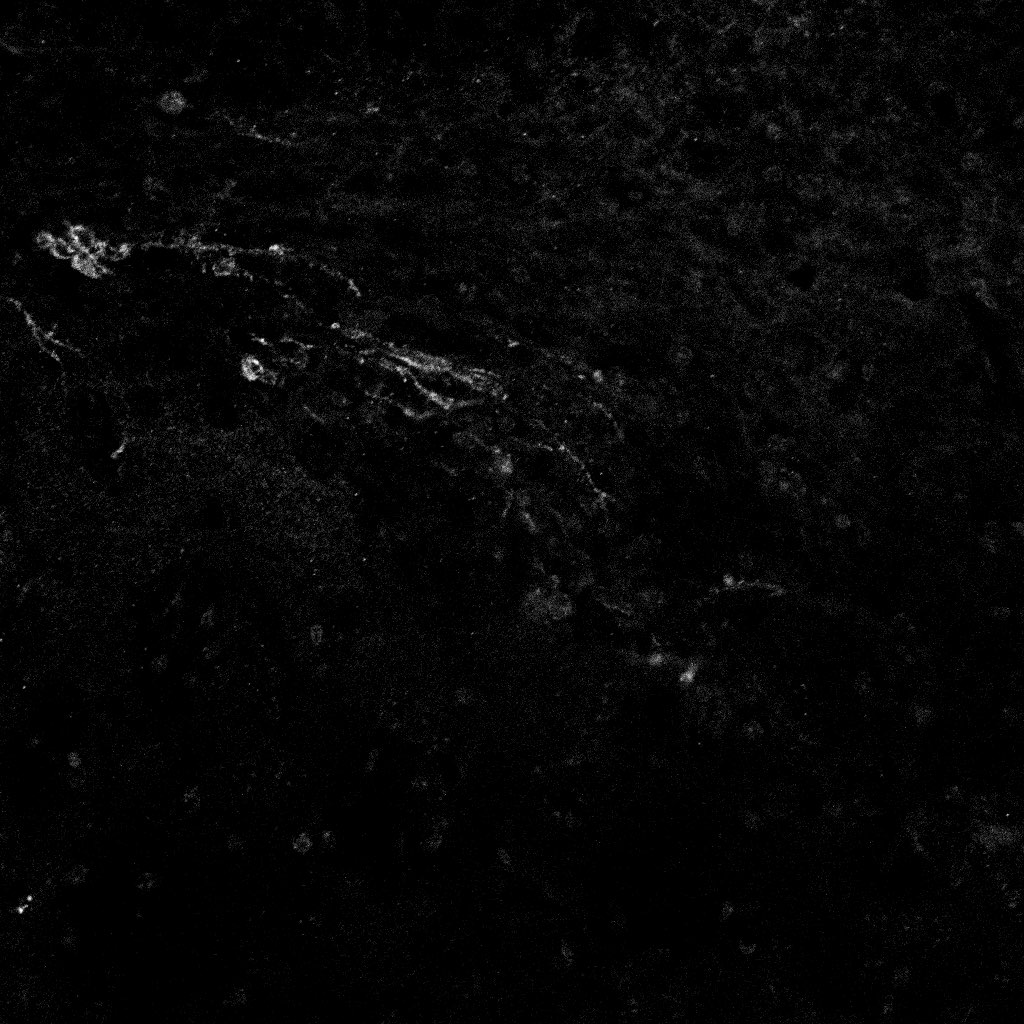

Supplement: Supplementary file 9 — Source data Fig. 4 [file 44321_2024_73_MOESM9_ESM.zip › Figure 4/4B/4B Ctrl PSA-NCAM.tif]

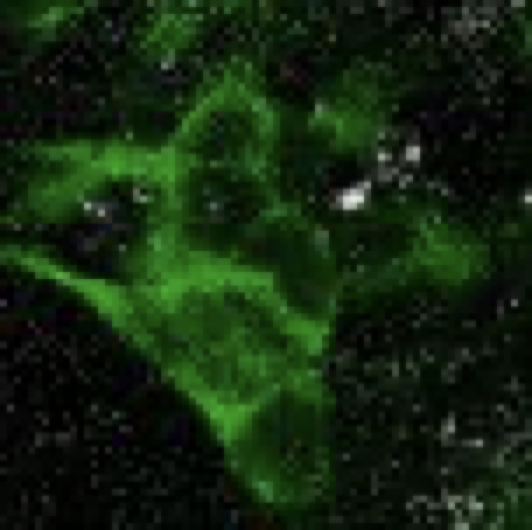

Supplement: Supplementary file 9 — Source data Fig. 4 [file 44321_2024_73_MOESM9_ESM.zip › Figure 4/4K/4K LPS+PBS Dcx, PSA-NCAM.tif]

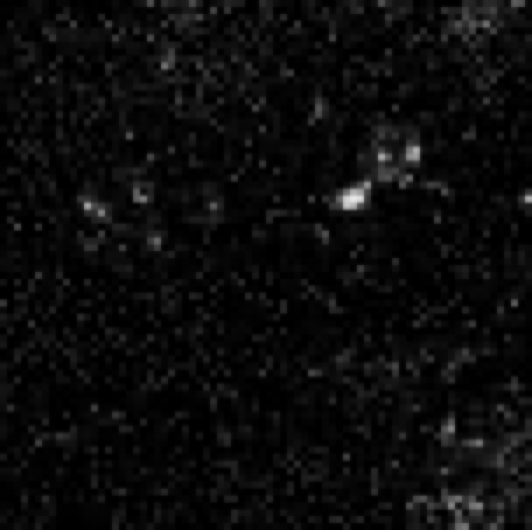

Supplement: Supplementary file 9 — Source data Fig. 4 [file 44321_2024_73_MOESM9_ESM.zip › Figure 4/4K/4K LPS+PBS PSA-NCAM.tif]

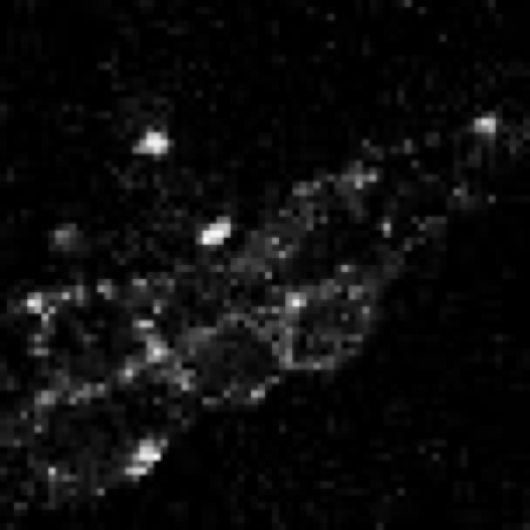

Supplement: Supplementary file 9 — Source data Fig. 4 [file 44321_2024_73_MOESM9_ESM.zip › Figure 4/4L/4L LPS+DANA PSA-NCAM.tif]

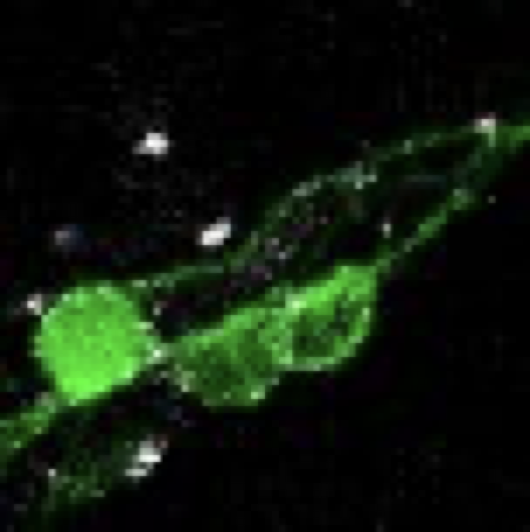

Supplement: Supplementary file 9 — Source data Fig. 4 [file 44321_2024_73_MOESM9_ESM.zip › Figure 4/4L/4L LPS+DANA Dcx, PSA-NCAM.tif]

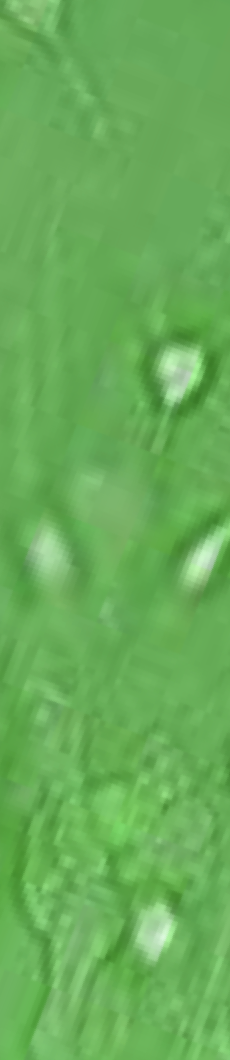

Supplement: Supplementary file 9 — Source data Fig. 4 [file 44321_2024_73_MOESM9_ESM.zip › Figure 4/4P/4P LPS+PBS 30min.tif]

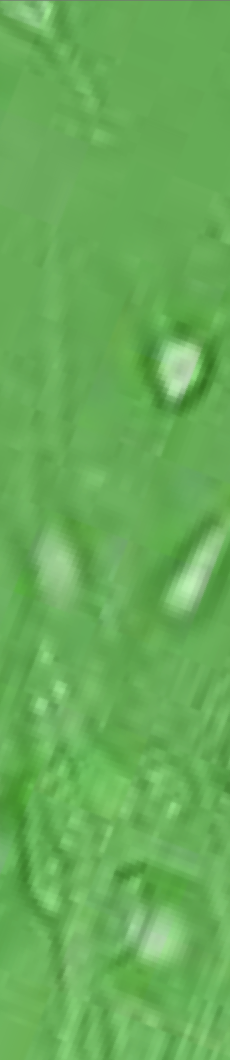

Supplement: Supplementary file 9 — Source data Fig. 4 [file 44321_2024_73_MOESM9_ESM.zip › Figure 4/4P/4P LPS+PBS 0min.tif]

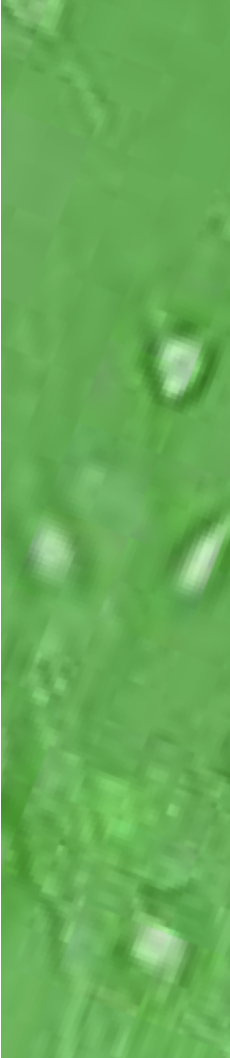

Supplement: Supplementary file 9 — Source data Fig. 4 [file 44321_2024_73_MOESM9_ESM.zip › Figure 4/4P/4P LPS+PBS 15min.tif]

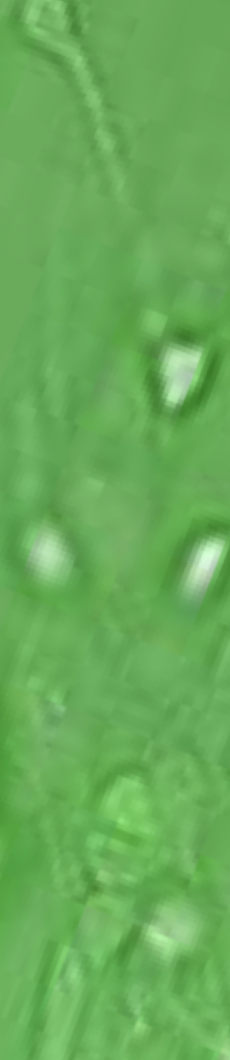

Supplement: Supplementary file 9 — Source data Fig. 4 [file 44321_2024_73_MOESM9_ESM.zip › Figure 4/4P/4P LPS+PBS 60min.tif]

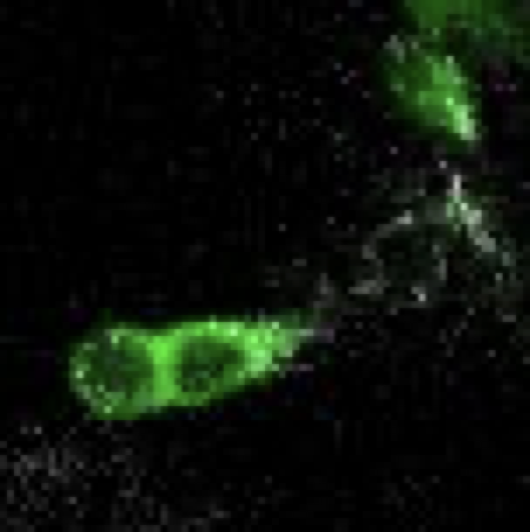

Supplement: Supplementary file 9 — Source data Fig. 4 [file 44321_2024_73_MOESM9_ESM.zip › Figure 4/4M/4M LPS+Zanamivir Dcx, PSA-NCAM.tif]

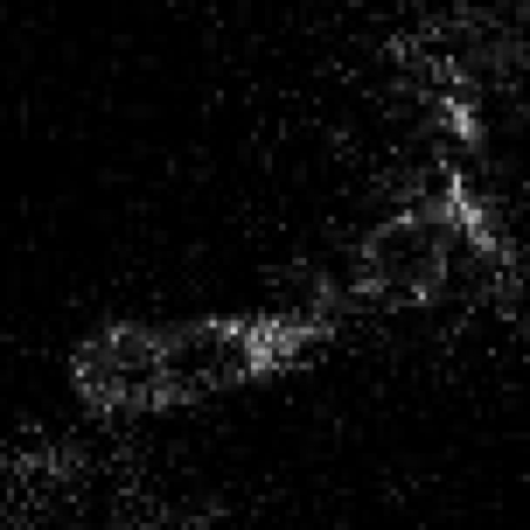

Supplement: Supplementary file 9 — Source data Fig. 4 [file 44321_2024_73_MOESM9_ESM.zip › Figure 4/4M/4M LPS+Zanamivir PSA-NCAM.tif]

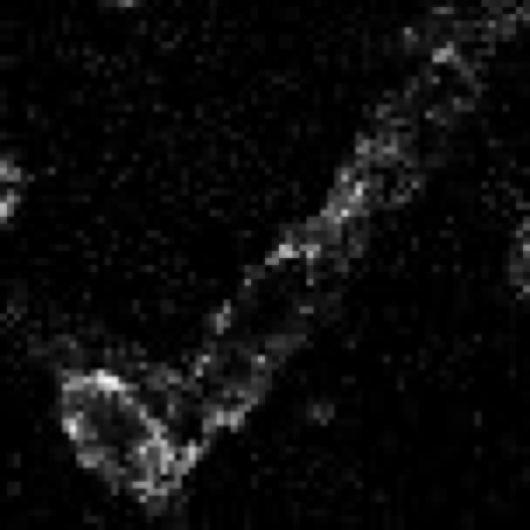

Supplement: Supplementary file 9 — Source data Fig. 4 [file 44321_2024_73_MOESM9_ESM.zip › Figure 4/4J/4J LPS-Ctrl PSA-NCAM.tif]

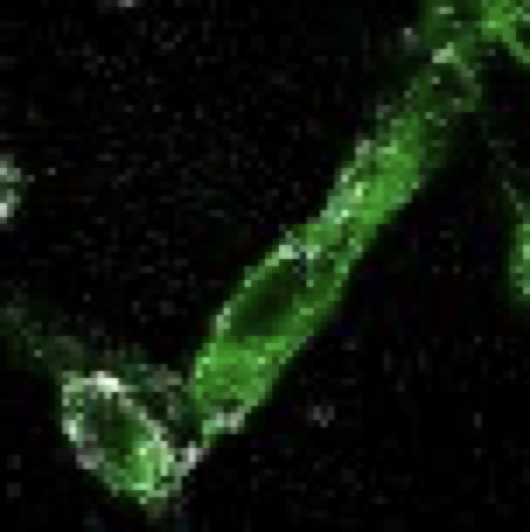

Supplement: Supplementary file 9 — Source data Fig. 4 [file 44321_2024_73_MOESM9_ESM.zip › Figure 4/4J/4J LPS-Ctrl Dcx, PSA-NCAM.tif]

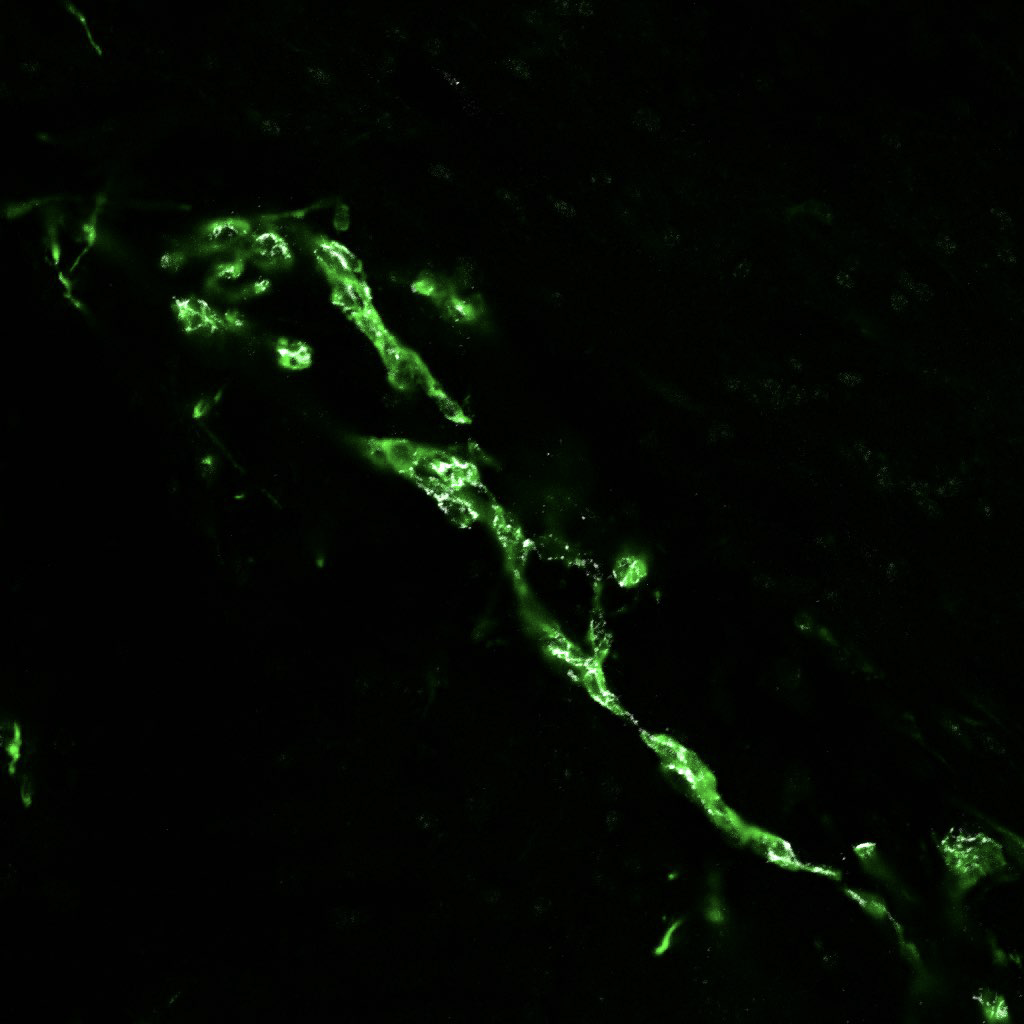

Supplement: Supplementary file 9 — Source data Fig. 4 [file 44321_2024_73_MOESM9_ESM.zip › Figure 4/4C/4C DANA Dcx, PSA-NCAM.tif]

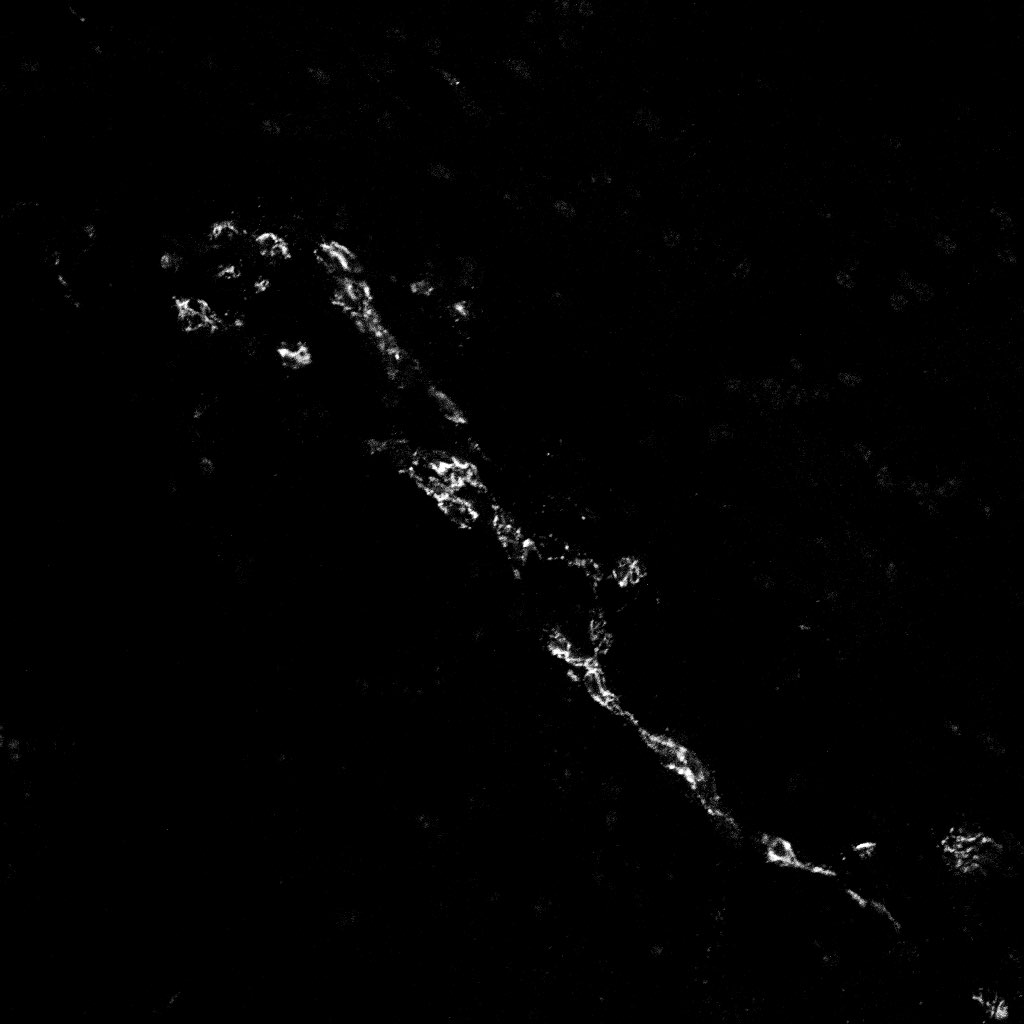

Supplement: Supplementary file 9 — Source data Fig. 4 [file 44321_2024_73_MOESM9_ESM.zip › Figure 4/4C/4C DANA PSA-NCAM.tif]

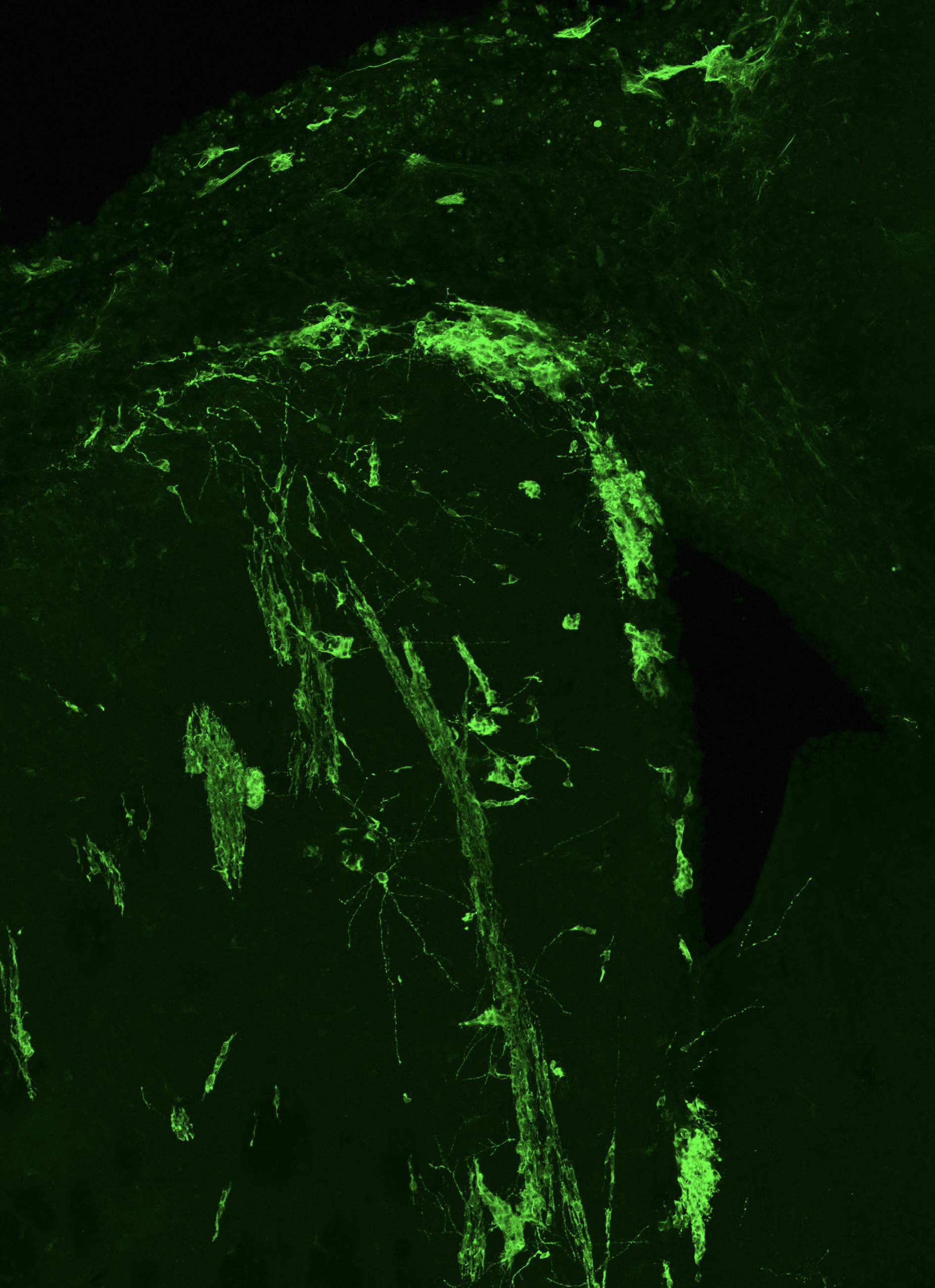

Supplement: Supplementary file 9 — Source data Fig. 4 [file 44321_2024_73_MOESM9_ESM.zip › Figure 4/4C/4C DANA Dcx.tif]

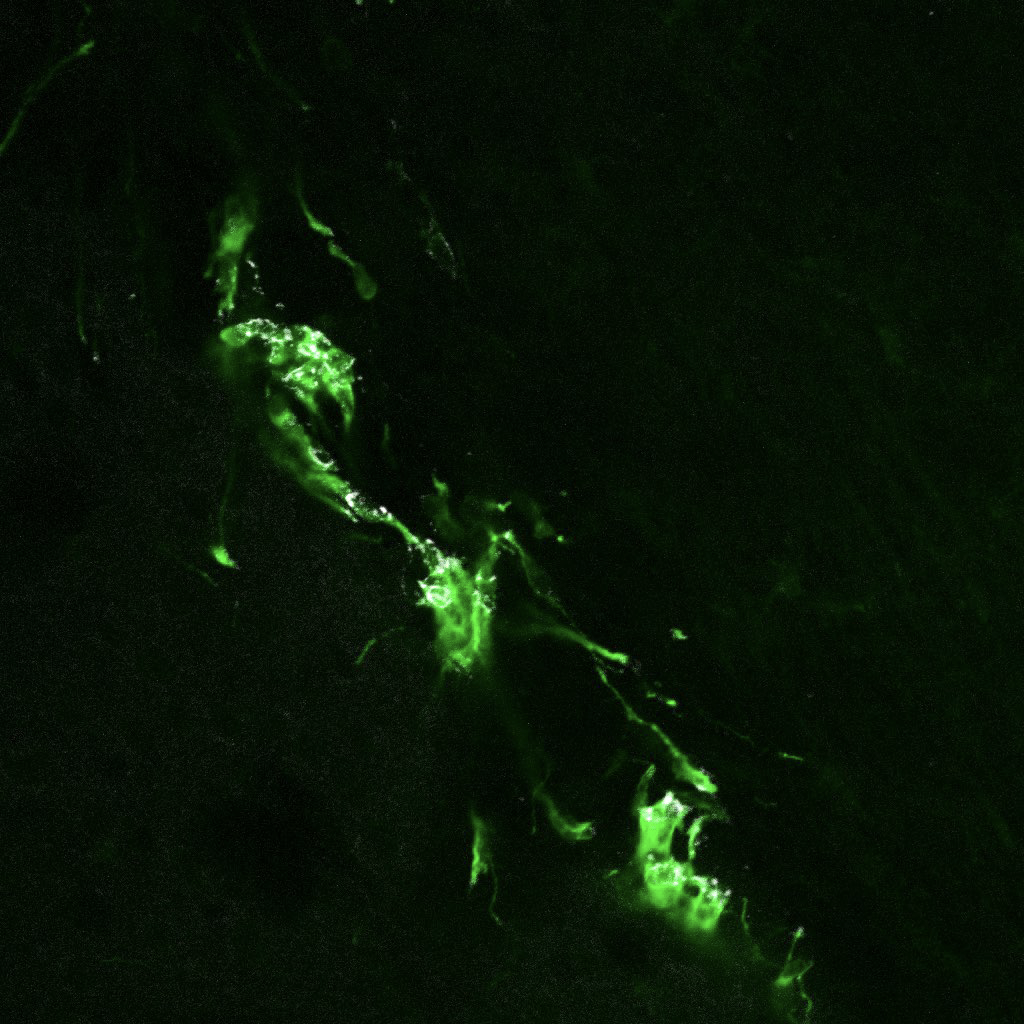

Supplement: Supplementary file 9 — Source data Fig. 4 [file 44321_2024_73_MOESM9_ESM.zip › Figure 4/4D/4D Zanamivir Dcx, PSA-NCAM.tif]

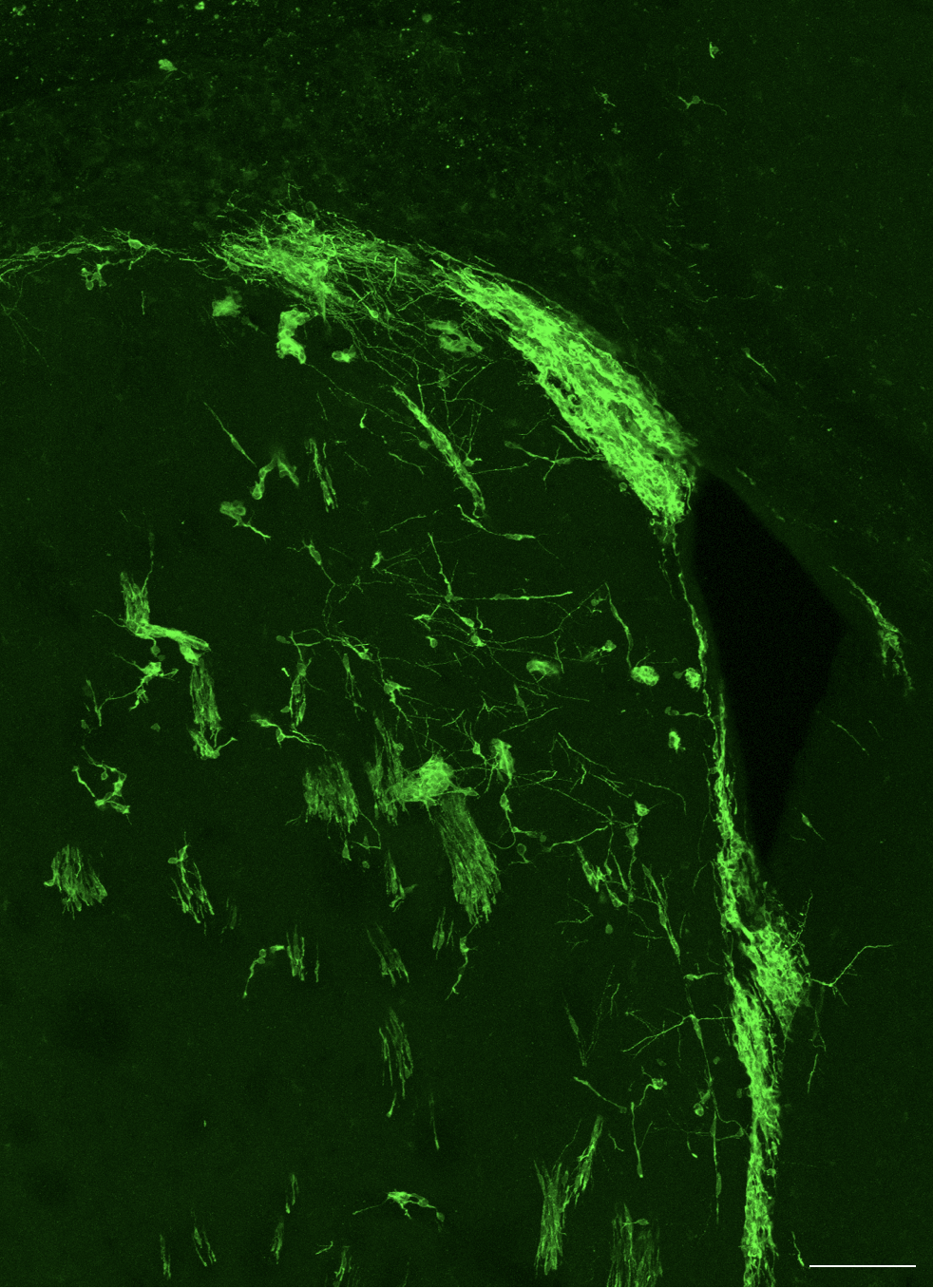

Supplement: Supplementary file 9 — Source data Fig. 4 [file 44321_2024_73_MOESM9_ESM.zip › Figure 4/4D/4D Zanamivir Dcx.tif]

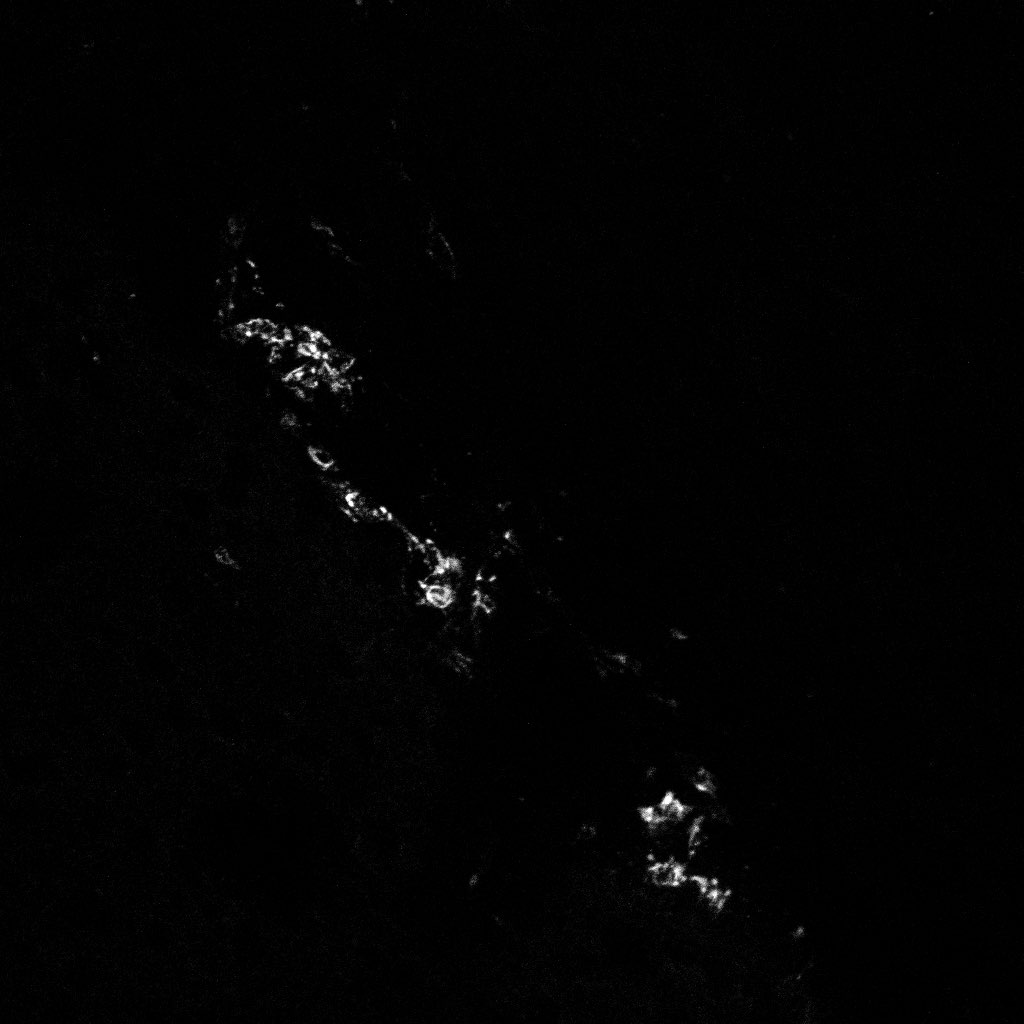

Supplement: Supplementary file 9 — Source data Fig. 4 [file 44321_2024_73_MOESM9_ESM.zip › Figure 4/4D/4D Zanamivir PSA-NCAM.tif]

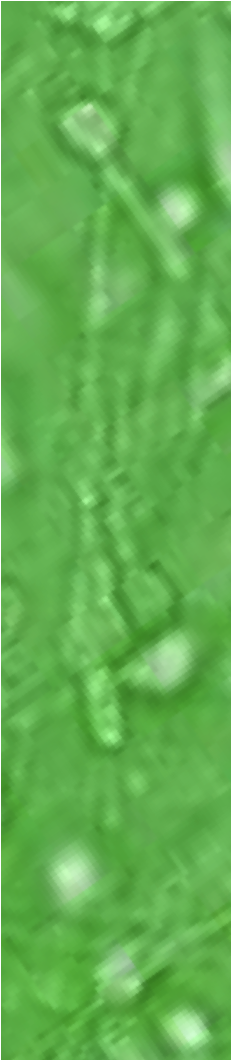

Supplement: Supplementary file 9 — Source data Fig. 4 [file 44321_2024_73_MOESM9_ESM.zip › Figure 4/4Q/4Q LPS+DANA 15min.tif]

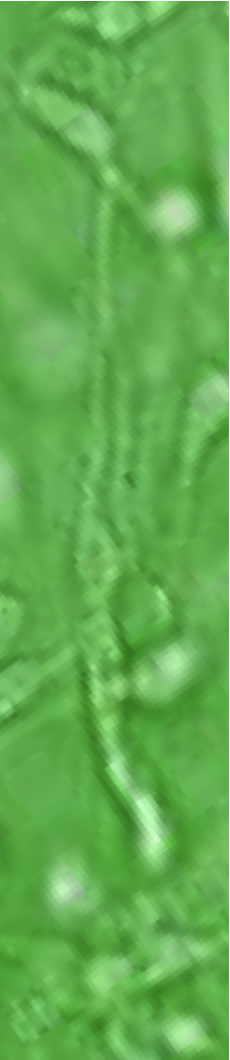

Supplement: Supplementary file 9 — Source data Fig. 4 [file 44321_2024_73_MOESM9_ESM.zip › Figure 4/4Q/4Q LPS+DANA 0min.tif]

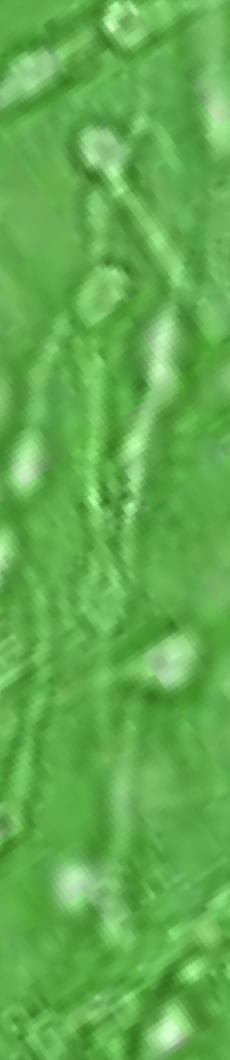

Supplement: Supplementary file 9 — Source data Fig. 4 [file 44321_2024_73_MOESM9_ESM.zip › Figure 4/4Q/4Q LPS+DANA 60min.tif]

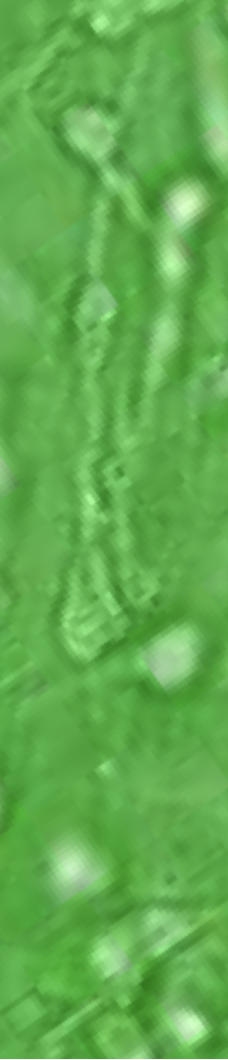

Supplement: Supplementary file 9 — Source data Fig. 4 [file 44321_2024_73_MOESM9_ESM.zip › Figure 4/4Q/4Q LPS+DANA 30min.tif]

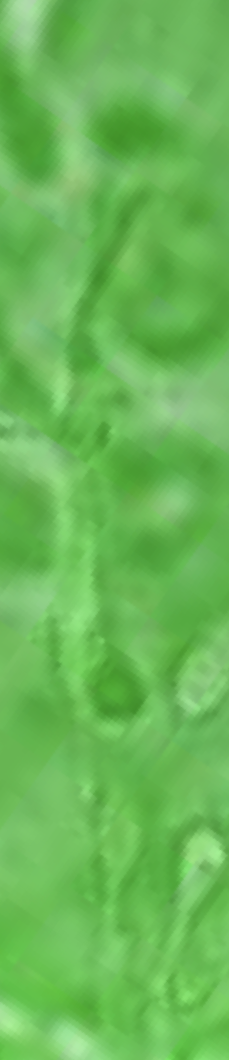

Supplement: Supplementary file 9 — Source data Fig. 4 [file 44321_2024_73_MOESM9_ESM.zip › Figure 4/4O/4O LPS-Ctrl 60min.tif]

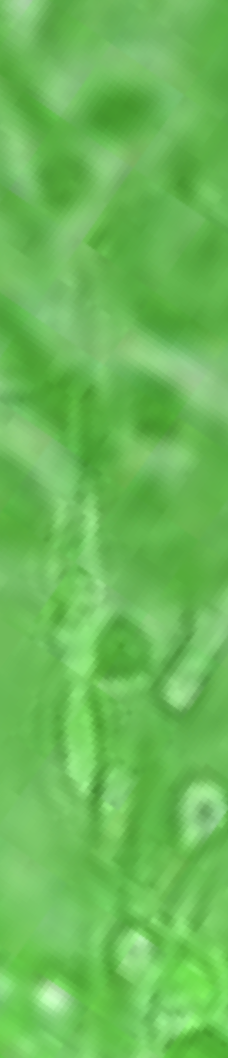

Supplement: Supplementary file 9 — Source data Fig. 4 [file 44321_2024_73_MOESM9_ESM.zip › Figure 4/4O/4O LPS-Ctrl 15min.tif]

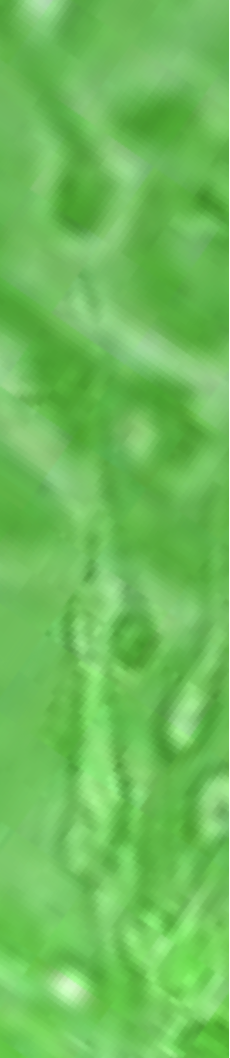

Supplement: Supplementary file 9 — Source data Fig. 4 [file 44321_2024_73_MOESM9_ESM.zip › Figure 4/4O/4O LPS-Ctrl 0min.tif]

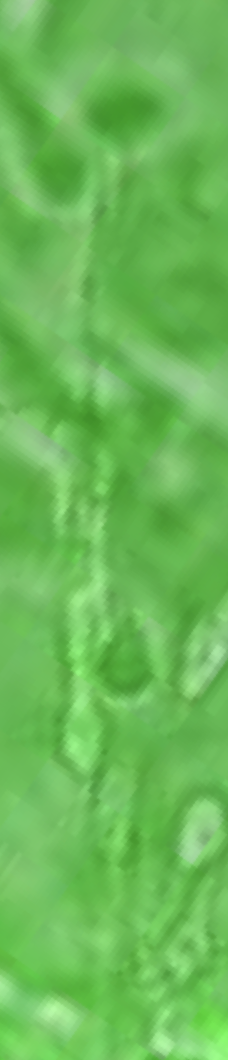

Supplement: Supplementary file 9 — Source data Fig. 4 [file 44321_2024_73_MOESM9_ESM.zip › Figure 4/4O/4O LPS-Ctrl 30min.tif]

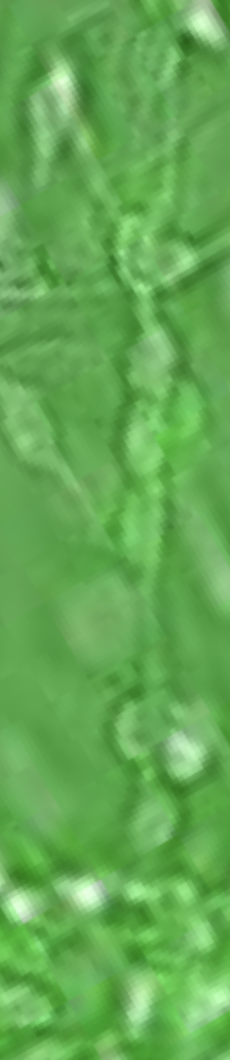

Supplement: Supplementary file 9 — Source data Fig. 4 [file 44321_2024_73_MOESM9_ESM.zip › Figure 4/4R/4R LPS+Zanamivir 60min.tif]

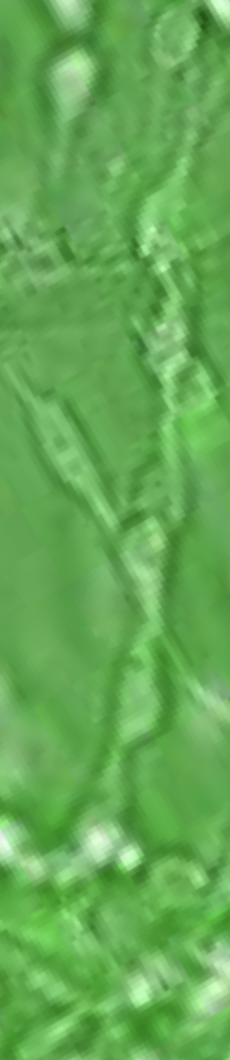

Supplement: Supplementary file 9 — Source data Fig. 4 [file 44321_2024_73_MOESM9_ESM.zip › Figure 4/4R/4R LPS+Zanamivir 15min.tif]

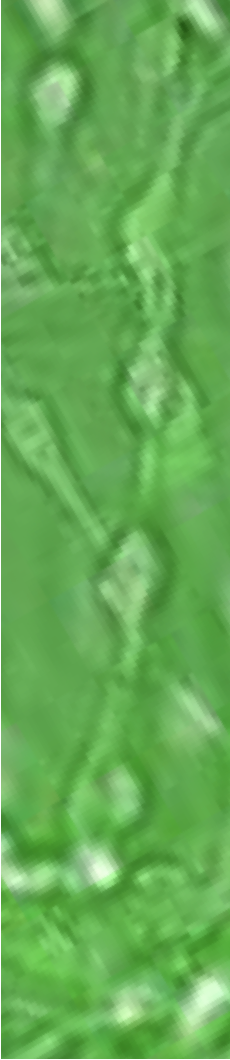

Supplement: Supplementary file 9 — Source data Fig. 4 [file 44321_2024_73_MOESM9_ESM.zip › Figure 4/4R/4R LPS+Zanamivir 0min.tif]

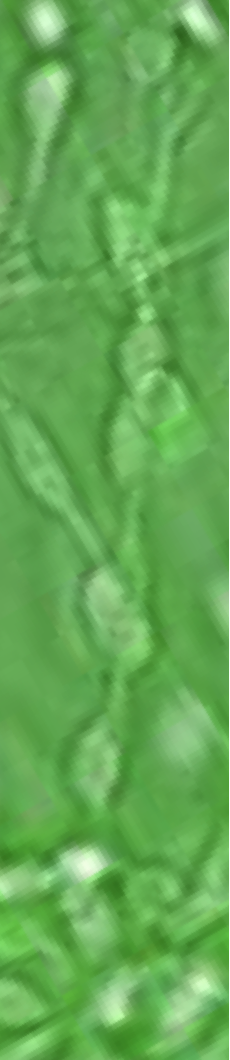

Supplement: Supplementary file 9 — Source data Fig. 4 [file 44321_2024_73_MOESM9_ESM.zip › Figure 4/4R/4R LPS+Zanamivir 30min.tif]

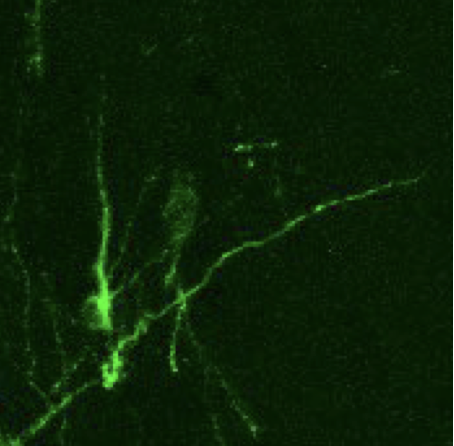

Supplement: Supplementary file 10 — Source data Fig. 5 [file 44321_2024_73_MOESM10_ESM.zip › Figure 5/5C/5C' DANA NeuN.tif]

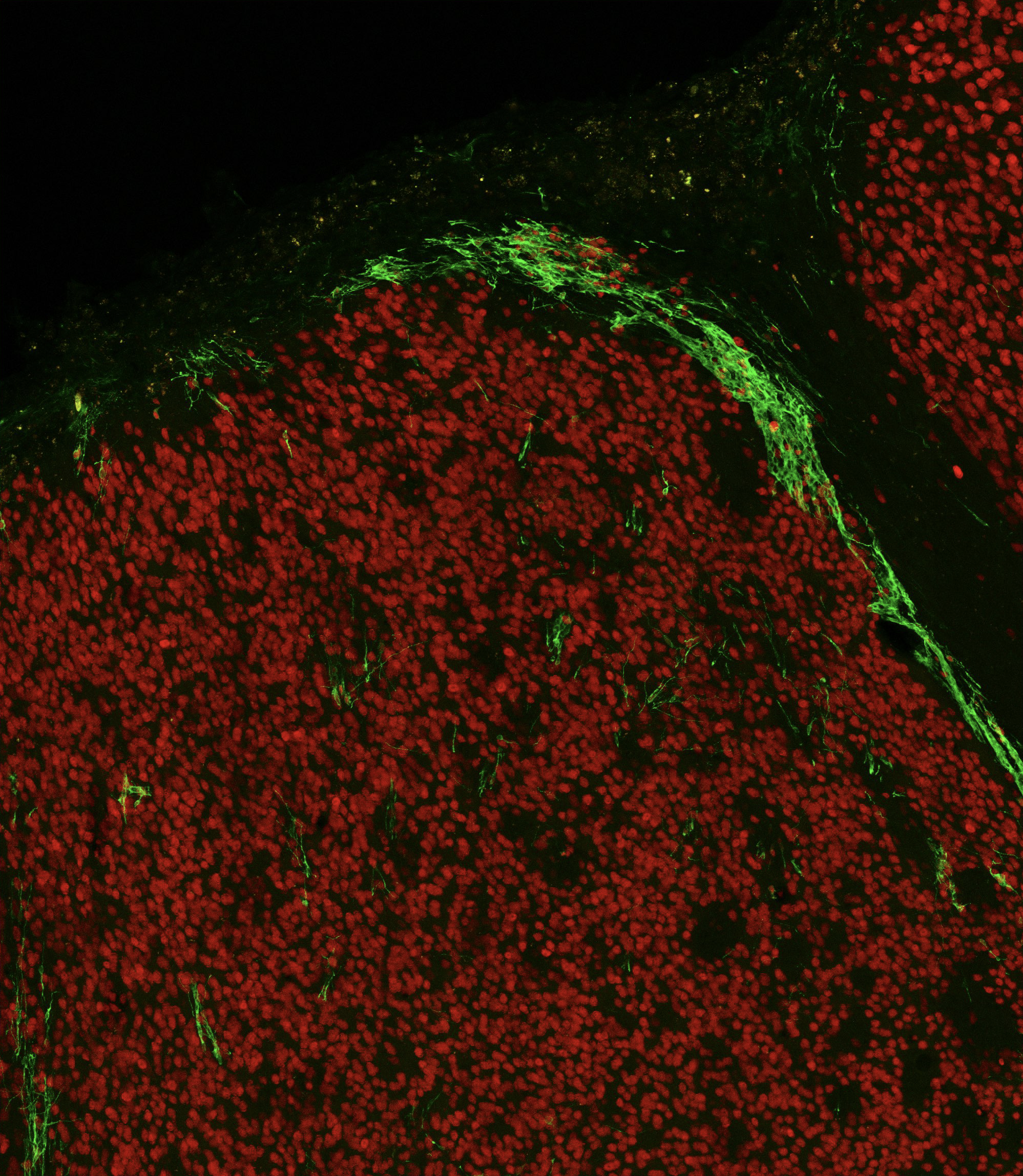

Supplement: Supplementary file 10 — Source data Fig. 5 [file 44321_2024_73_MOESM10_ESM.zip › Figure 5/5C/5C DANA Dcx, NeuN.tif]

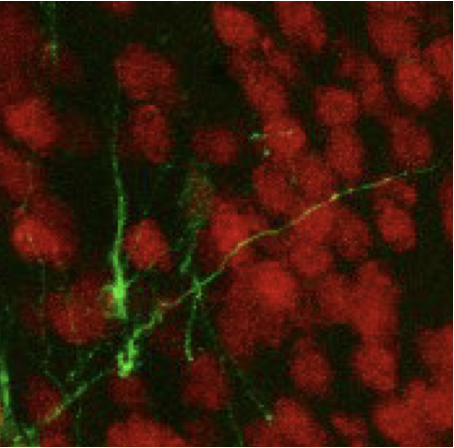

Supplement: Supplementary file 10 — Source data Fig. 5 [file 44321_2024_73_MOESM10_ESM.zip › Figure 5/5C/5C' DANA Dcx, NeuN.tif]

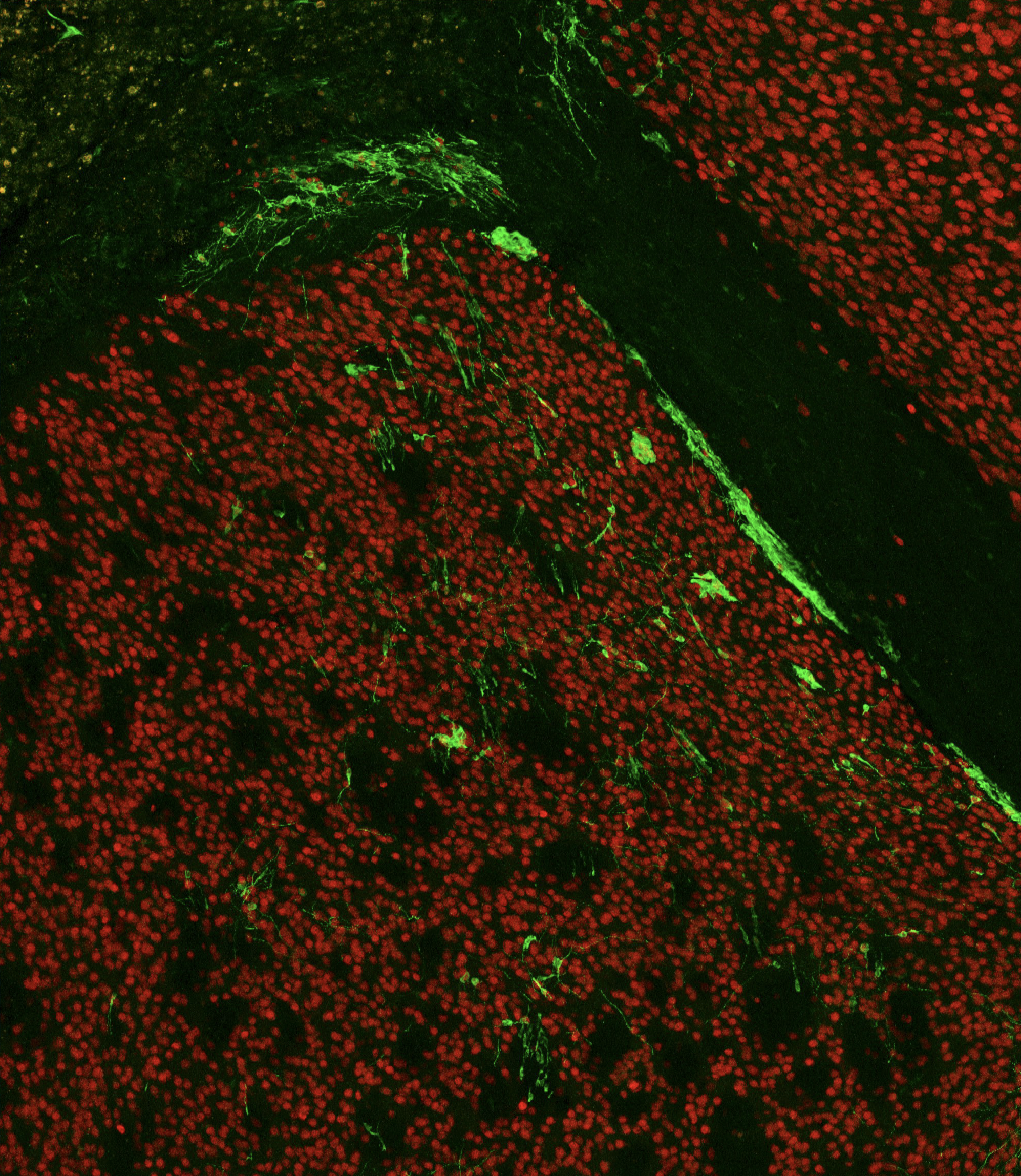

Supplement: Supplementary file 10 — Source data Fig. 5 [file 44321_2024_73_MOESM10_ESM.zip › Figure 5/5D/5D Zanamivir Dcx, NeuN.tif]

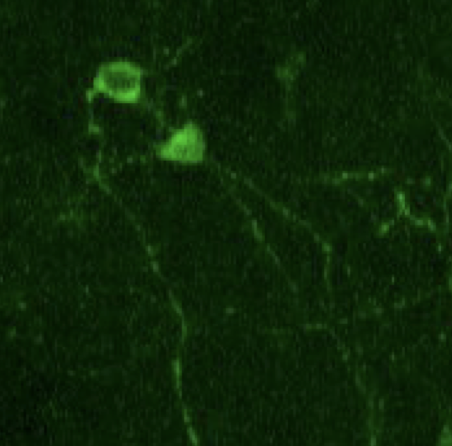

Supplement: Supplementary file 10 — Source data Fig. 5 [file 44321_2024_73_MOESM10_ESM.zip › Figure 5/5D/5D' Zanamivir NeuN.tif]

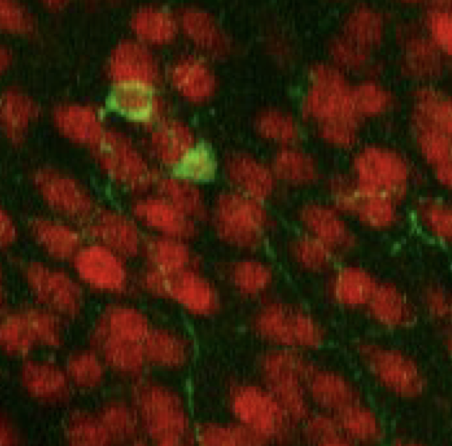

Supplement: Supplementary file 10 — Source data Fig. 5 [file 44321_2024_73_MOESM10_ESM.zip › Figure 5/5D/5D' Zanamivir Dcx, NeuN.tif]

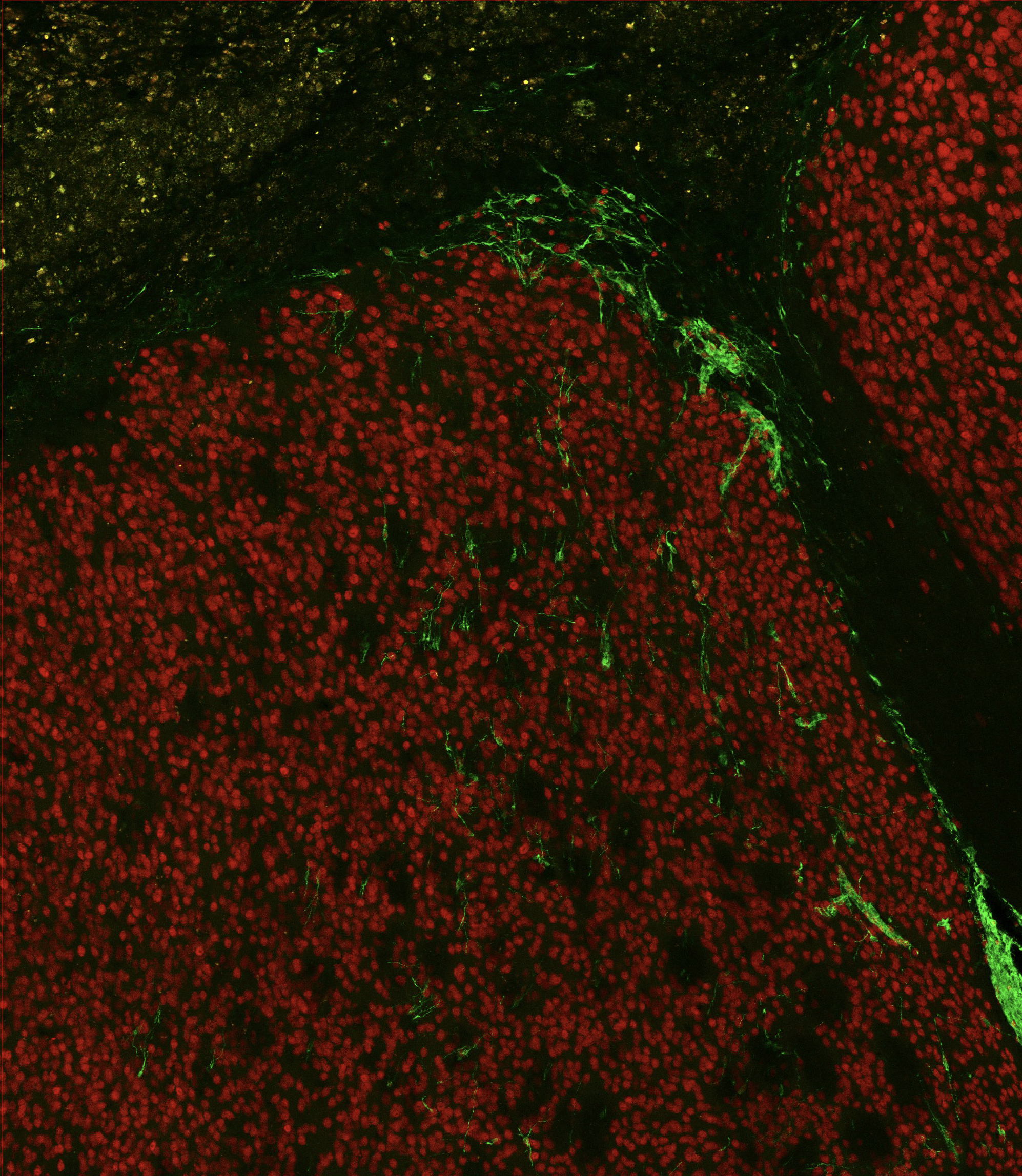

Supplement: Supplementary file 10 — Source data Fig. 5 [file 44321_2024_73_MOESM10_ESM.zip › Figure 5/5B/5B Ctrl Dcx, NeuN.tif]

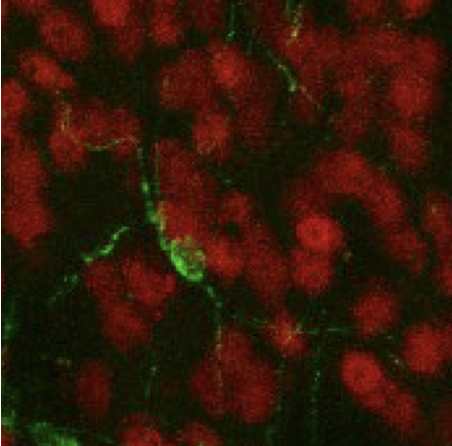

Supplement: Supplementary file 10 — Source data Fig. 5 [file 44321_2024_73_MOESM10_ESM.zip › Figure 5/5B/5B' Ctrl Dcx, NeuN.tif]

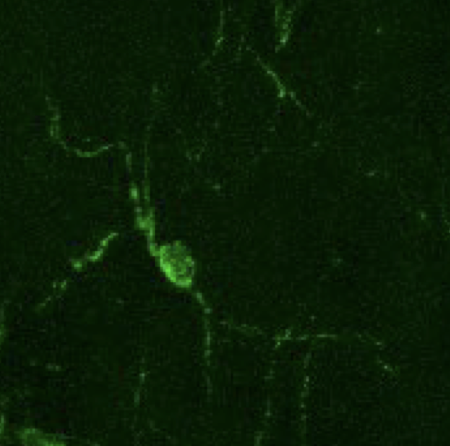

Supplement: Supplementary file 10 — Source data Fig. 5 [file 44321_2024_73_MOESM10_ESM.zip › Figure 5/5B/5B' Ctrl NeuN.tif]

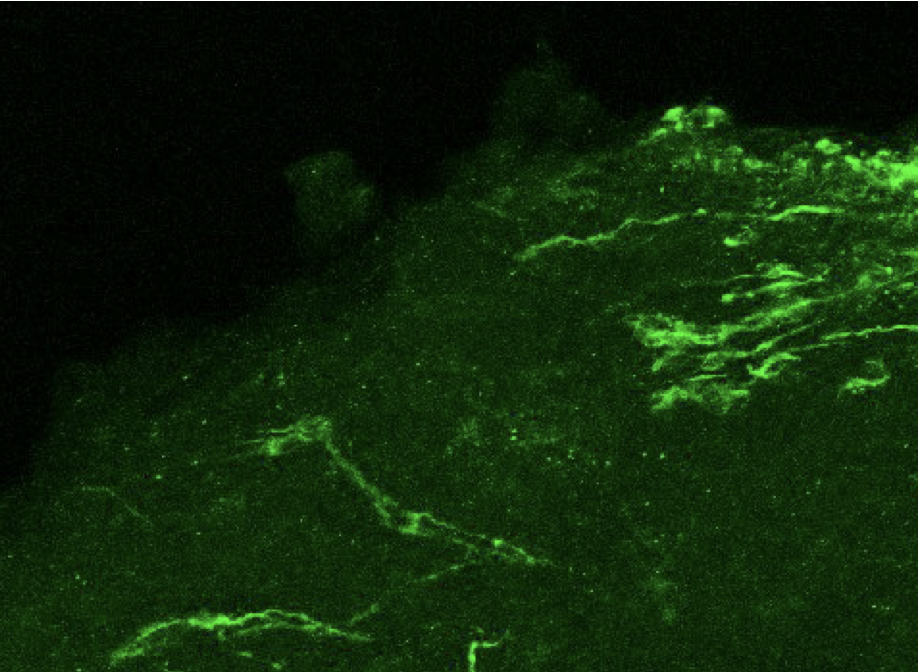

Supplement: Supplementary file 11 — Source data Fig. 6 [file 44321_2024_73_MOESM11_ESM.zip › Figure 6/6F/6F' Ctrl Dcx.tif]

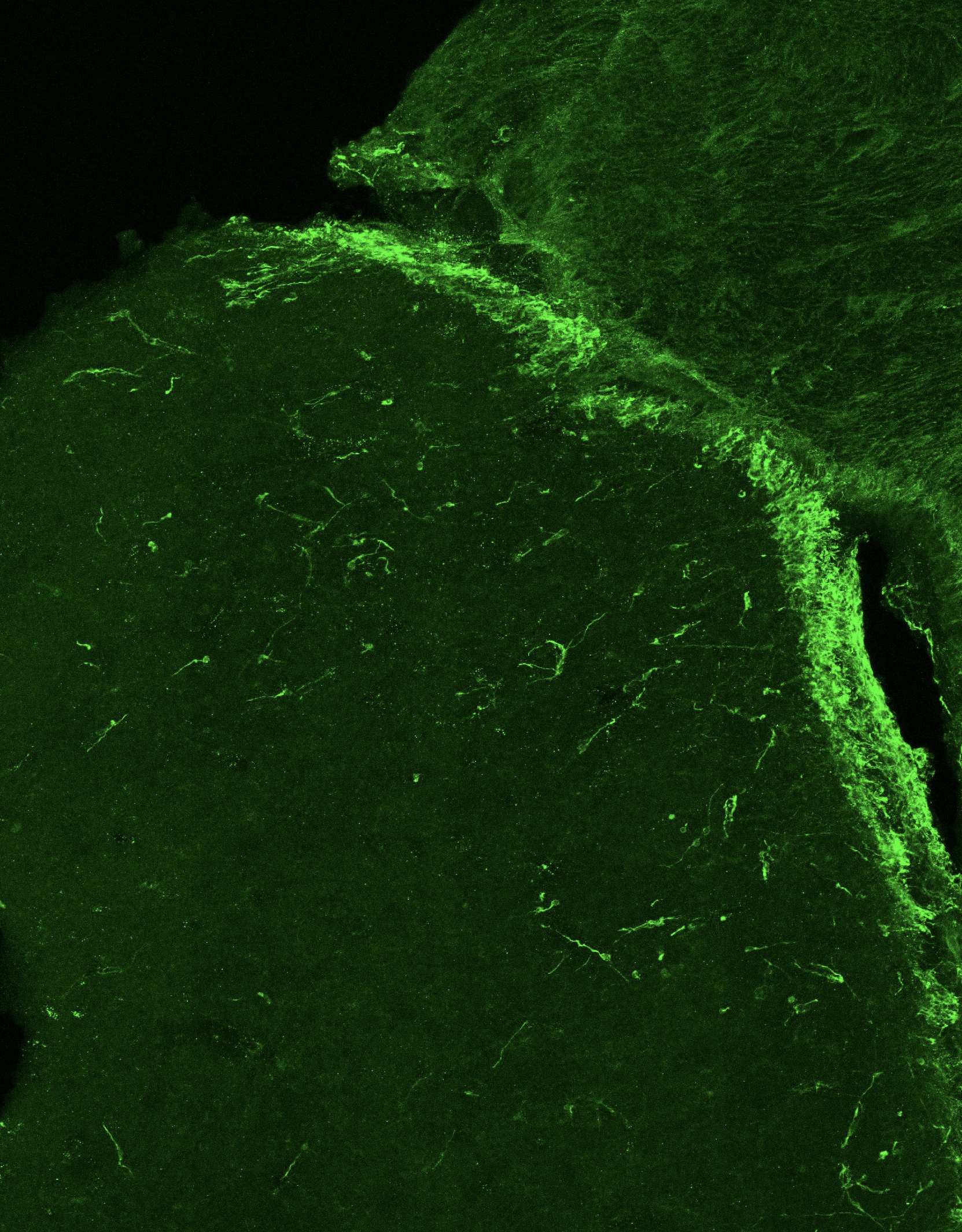

Supplement: Supplementary file 11 — Source data Fig. 6 [file 44321_2024_73_MOESM11_ESM.zip › Figure 6/6F/6F Ctrl Dcx.tif]

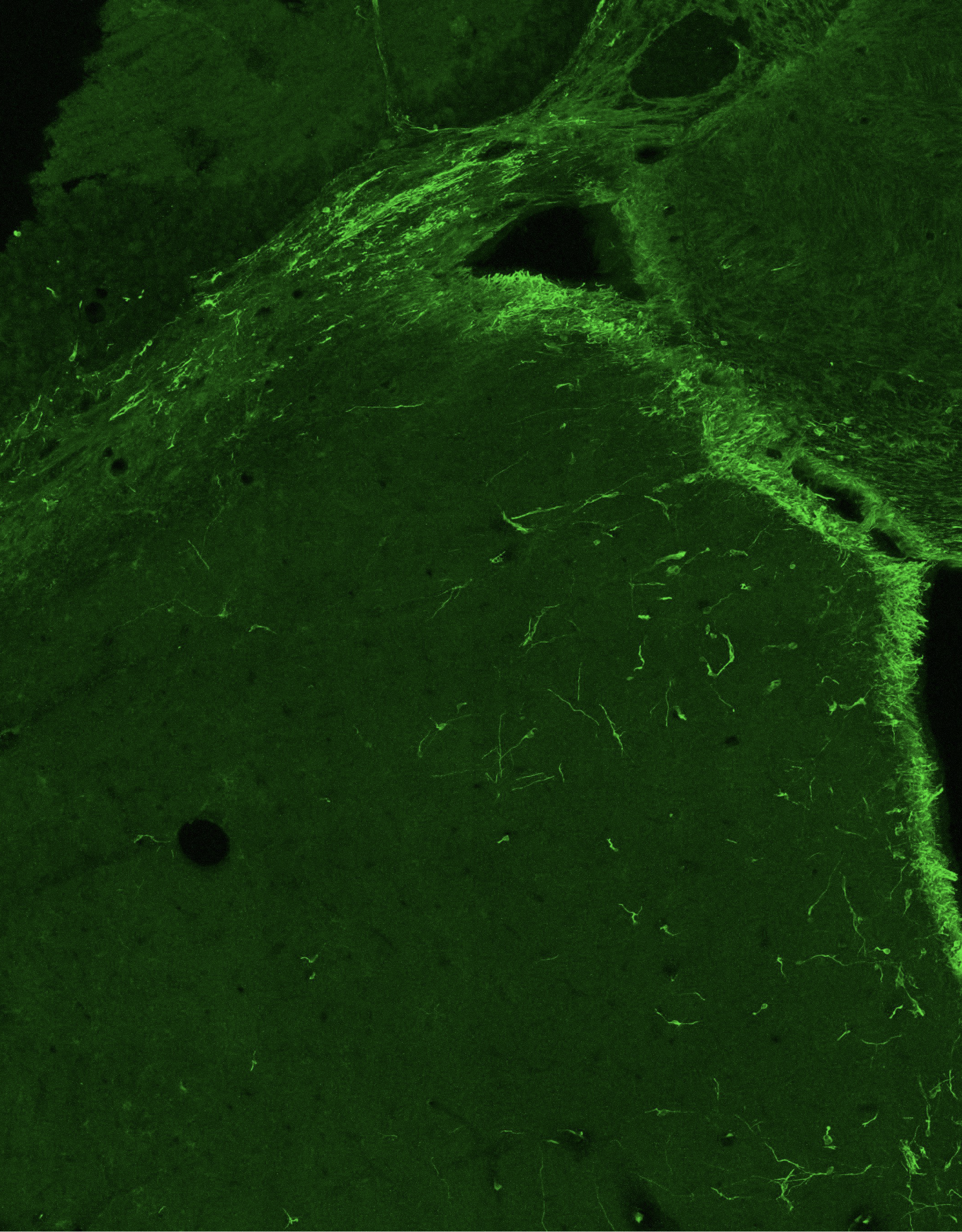

Supplement: Supplementary file 11 — Source data Fig. 6 [file 44321_2024_73_MOESM11_ESM.zip › Figure 6/6G/6G Zanamivir Dcx.tif]

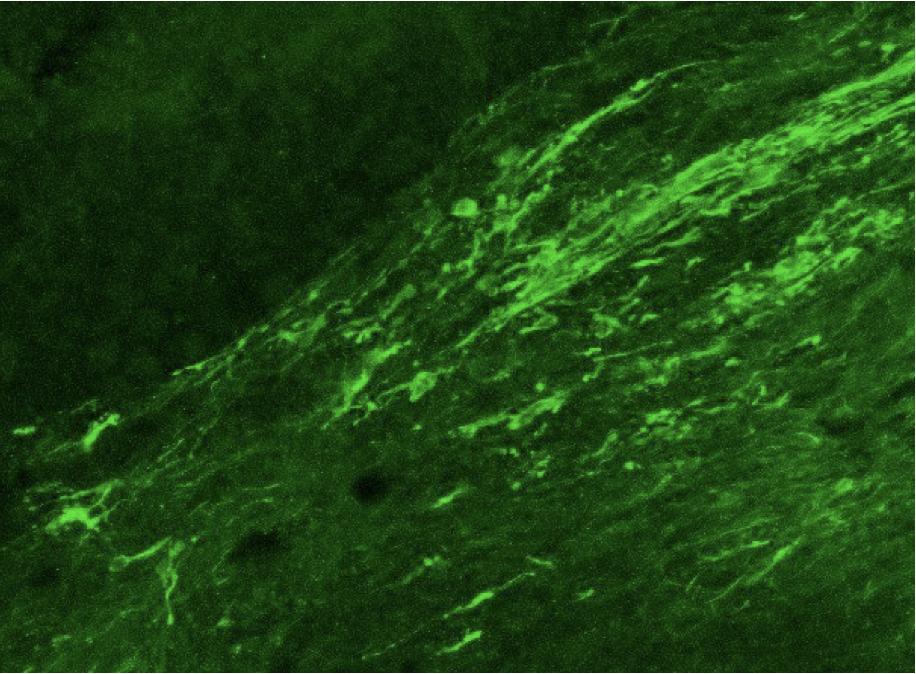

Supplement: Supplementary file 11 — Source data Fig. 6 [file 44321_2024_73_MOESM11_ESM.zip › Figure 6/6G/6G' Zanamivir Dcx.tif]

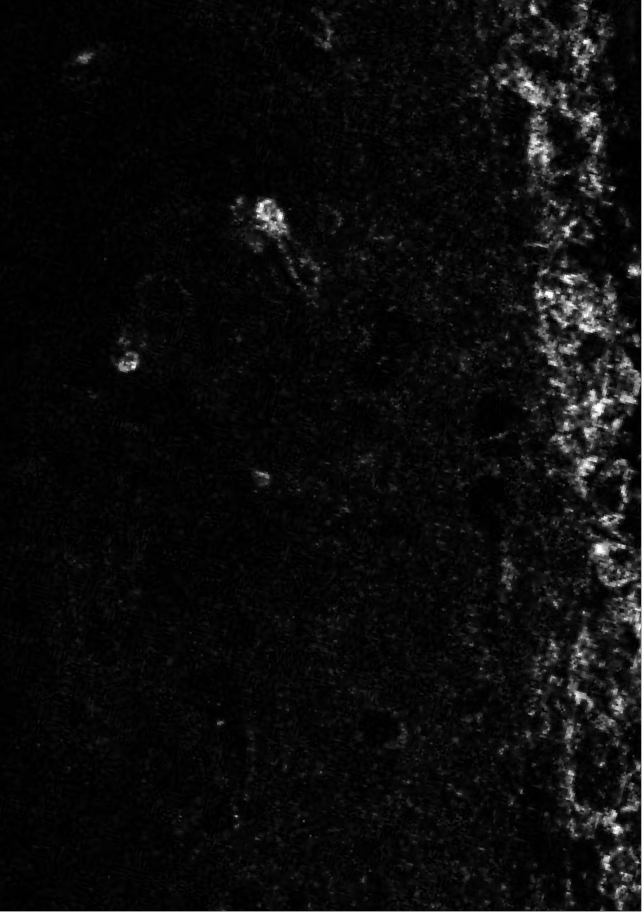

Supplement: Supplementary file 11 — Source data Fig. 6 [file 44321_2024_73_MOESM11_ESM.zip › Figure 6/6B/6B Ctrl PSA-NCAM.tif]
