# Supplementary figures and images for: Neuraminidase inhibition promotes the collective migration of neurons and recovery of brain function (part 2 of 2)
Source: EMBO Mol Med. 2024 May 24;16(6):1228–53. doi: 10.1038/s44321-024-00073-7 (PMC11178813; doi:10.1038/s44321-024-00073-7)

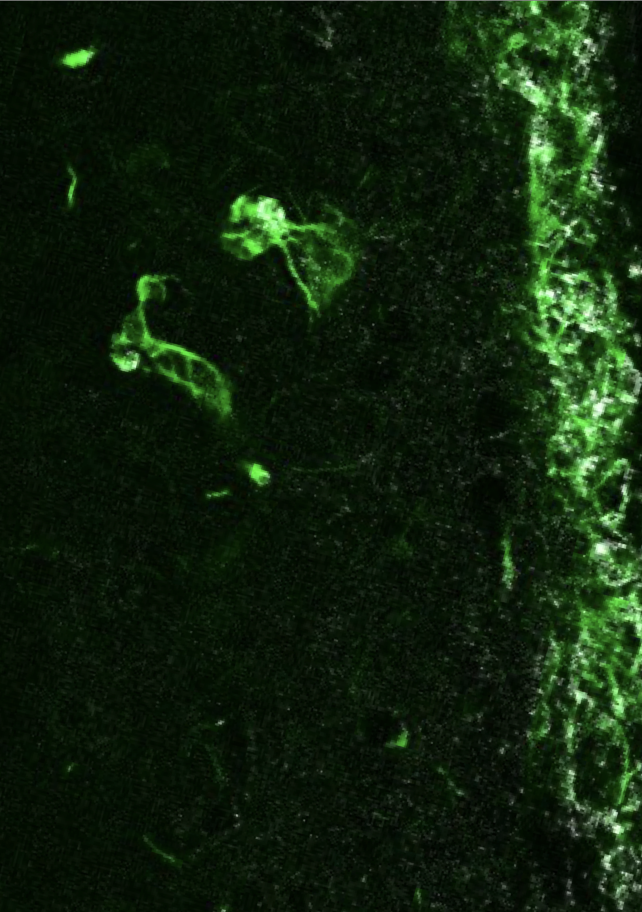

Supplement: Supplementary file 11 — Source data Fig. 6 [file 44321_2024_73_MOESM11_ESM.zip › Figure 6/6B/6B Ctrl Dcx, PSA-NCAM.tif]

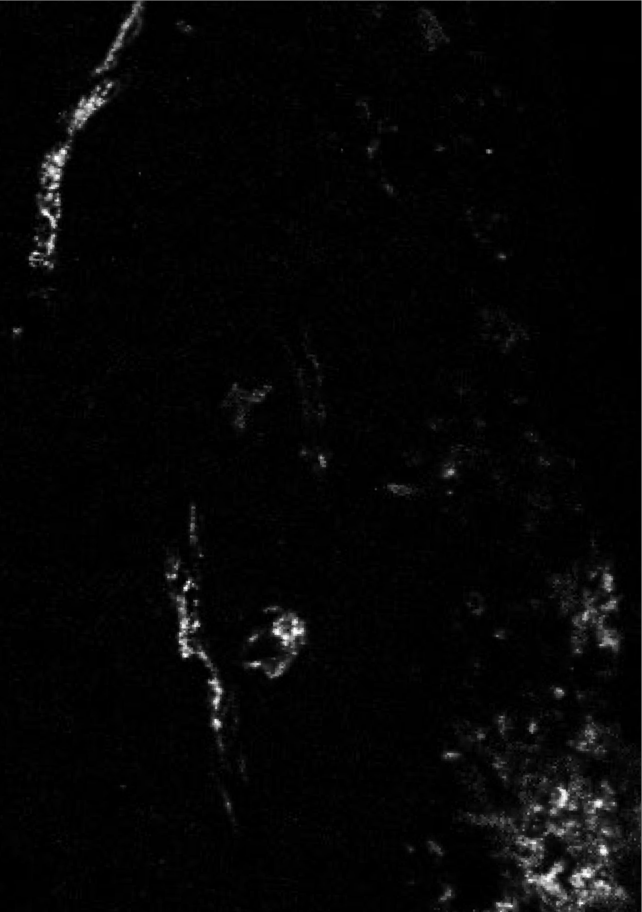

Supplement: Supplementary file 11 — Source data Fig. 6 [file 44321_2024_73_MOESM11_ESM.zip › Figure 6/6C/6C Zanamivir PSA-NCAM.tif]

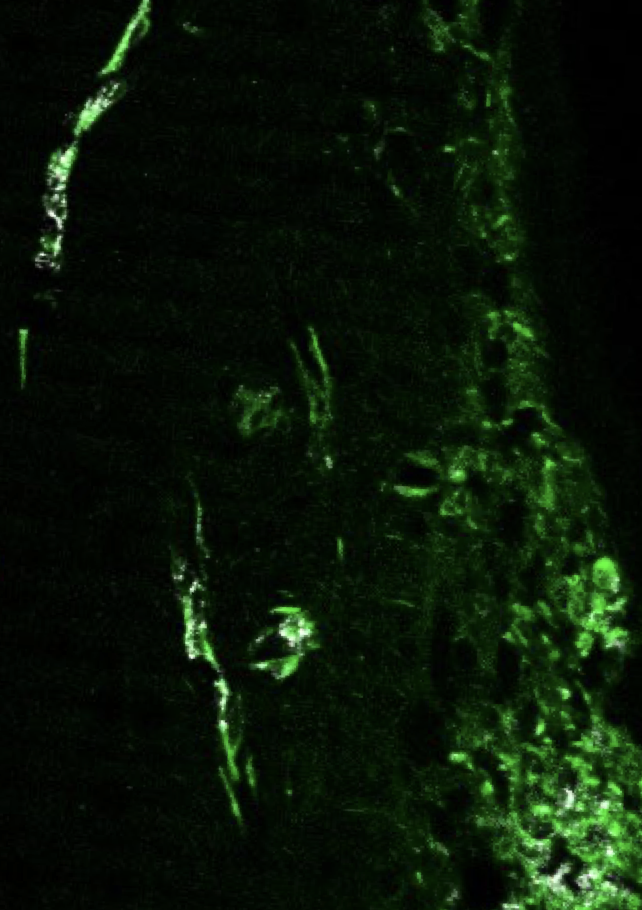

Supplement: Supplementary file 11 — Source data Fig. 6 [file 44321_2024_73_MOESM11_ESM.zip › Figure 6/6C/6C Zanamivir Dcx, PSA-NCAM.tif]
