# Supplementary material for: Neuraminidase inhibition promotes the collective migration of neurons and recovery of brain function
Source: EMBO Mol Med. 2024 May 24;16(6):1228–53. doi: 10.1038/s44321-024-00073-7 (PMC11178813; doi:10.1038/s44321-024-00073-7)
Supplement: Supplementary file 13 — Expanded View Figures [file 44321_2024_73_MOESM13_ESM.pdf]

Expanded View Figures

Figure EV1. Semi-automatic segmentation of SBF-SEM images.

(**A**) Overview of the semi-automatic segmentation flow incorporating deep learning-based cell area predictions and Seg2Link for segmentation. (**B–B″**) The automatic segmentation results of each new neuron (**B**), the updated segmentation results with additional cell boundaries (1 pixel in x-y plane) surrounding each neuron, which is required for accurately evaluating the non-adherent area and cell adhesion (**B′**), and the final segmentation results with manual corrections (**B″**). All the three steps were performed in Seg2Link. (**C–E′**) Representative electron microscopy images of new neurons in normal RMS by transmission electron microscopy (TEM) (**C**, **C′**), TEM of a high-pressure freeze-treated sample (High-pressure frozen) (**D**, **D'**), and serial block face scanning electron microscopy (SBF-SEM) (**E**, **E′**). Blue arrows indicate non-adherent areas. Scale bars: 2 μm (**C**, **D**, **E**), 500 nm (**C′**, **D′**, **E′**). (**F**) The distance in the non-adherent area between neighboring cells in normal RMS quantified using TEM ($n = 46$ cells), high-pressure freezing ($n = 51$ cells), and SBF-SEM ($n = 147$ cells); Mann–Whitney U test. (**G**) Percentage of adhesion or adherens junction (AJ)-like adhesions and non-adherent areas in normal RMS quantified using TEM, high-pressure freezing, and SBF-SEM (closed) and (open); each group $n = 5$ cells; Tukey multiple comparisons of means. (**H–I′**) Representative images of three-dimensional reconstruction of neuronal chains in adult RMS. H′ and I′ show adhesion area (red) between new neuron (yellow) and new neuron (green). The new neuron has a smooth morphology, with numerous small adhesions. In contrast, the irregularly shaped new neurons show large adhesions. The boxes in (**H**, **I**) are magnified in (**H'**, **I'**), respectively. Scale bars: 5 μm (**H**, **I**), 1 μm (**H′**, **I′**). Data information: In (**F**, **G**), data are presented as mean ± SEM. $^{***}P < 0.005$, $^{****}P < 0.001$.

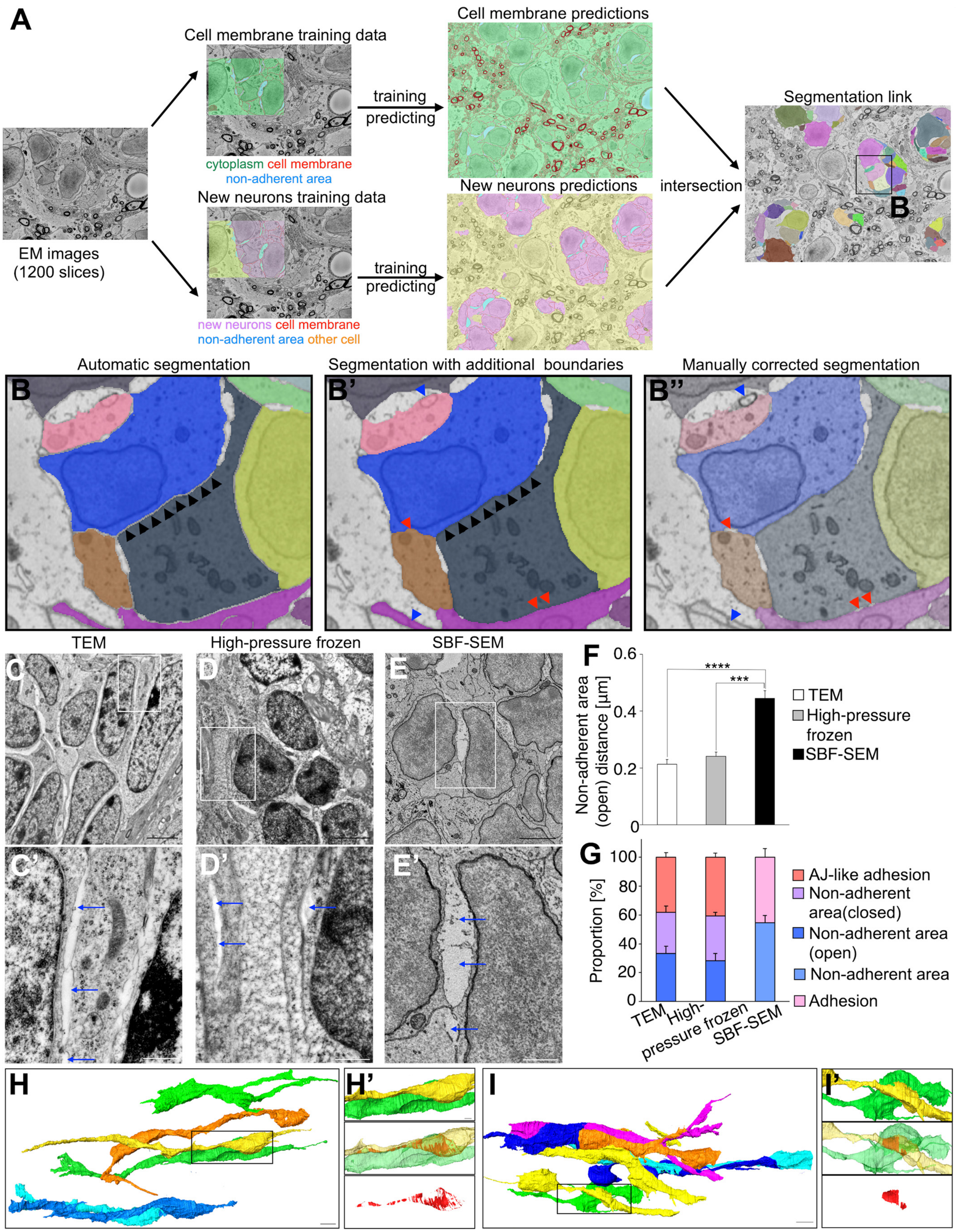
A
Cell membrane training data
Cell membrane predictions
training
predicting
cytoplasm cell membrane
non-adherent area
New neurons training data
New neurons predictions
intersection
Segmentation link
EM images
(1200 slices)
new neurons cell membrane
non-adherent area other cell
Automatic segmentation
Segmentation with additional boundaries
Manually corrected segmentation
B
B'
B''
TEM
High-pressure frozen
SBF-SEM
C
D
E
C'
D'
E'
F
0.6
0.4
0.2
0
Non-adherent area (open) distance [μm]
****
***
TEM
High-pressure frozen
SBF-SEM
G
100
80
60
40
20
0
Proportion [%]
AJ-like adhesion
Non-adherent area(closed)
Non-adherent area (open)
Non-adherent area
Adhesion
TEM
High-pressure frozen
SBF-SEM
H
H'
I
I'

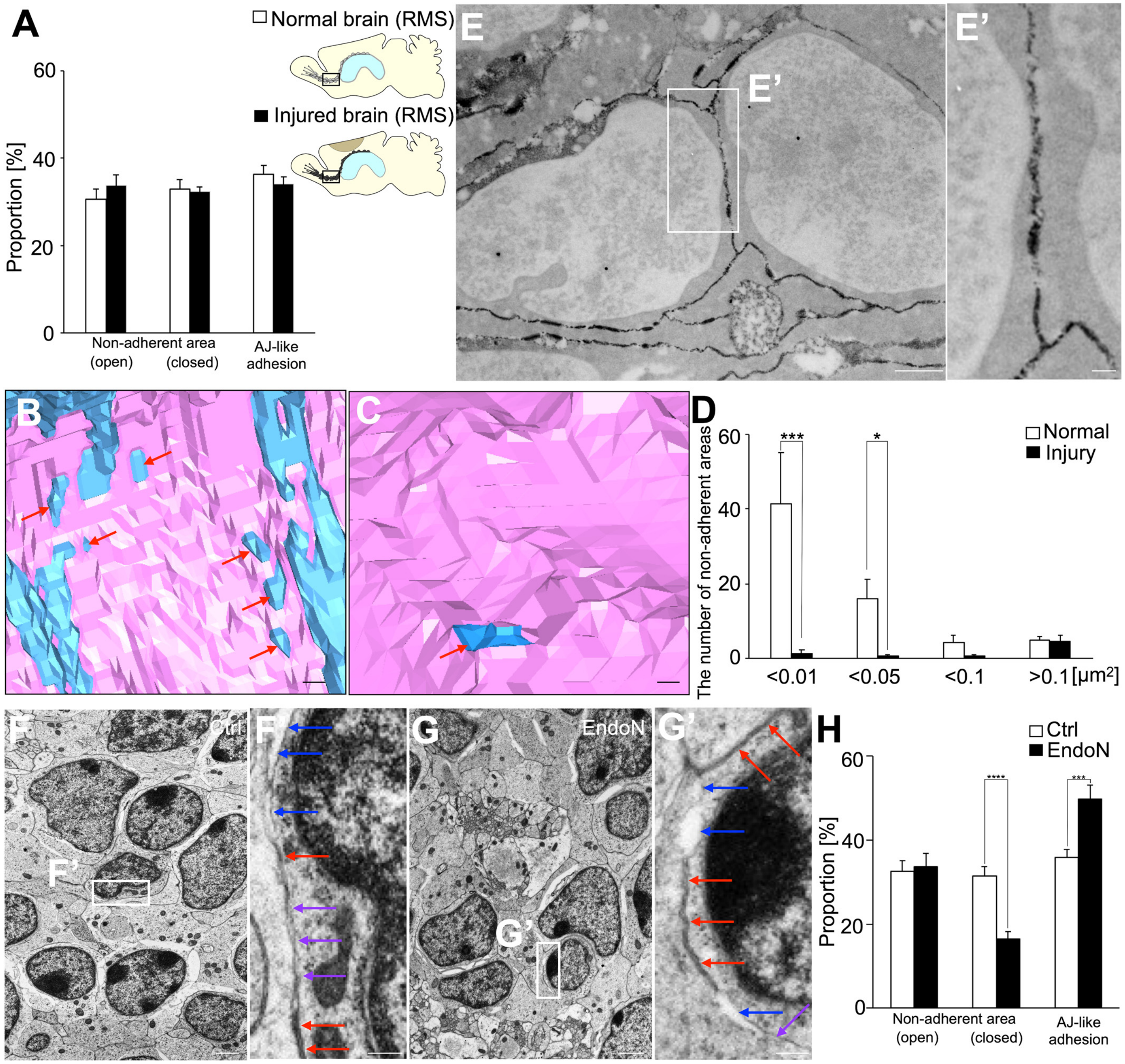

Figure EV2. PSA regulates AJ-like adhesions and non-adherent areas in migrating new neurons.

(**A**) Percentage of AJ-like adhesion and non-adherent areas ([open] or [closed]) in RMS in the normal brain and injured brain; each group $n = 30$ cells; non-adherent areas [open] and AJ-like adhesion; *t*-test, non-adherent areas [close]; Wilcoxon rank sum test. (**B**, **C**) Distribution of non-adhesion areas and adhesions to new neurons in neuronal cell bodies of adult RMS and injured brains. Pink and blue indicate adhesion to new neurons and non-adherent areas, respectively. Red arrows indicate pores. (**B**) and (**C**) show enlarged images of Fig. 2F′ and G′, respectively. Scale bars: 10 μm. (**D**) The number of non-adherent areas of each size in normal RMS ($n = 6$ adhesions) and injured brain ($n = 9$ adhesions); <0.01, <0.05, <0.1; Wilcoxon rank sum test, >0.1; *t*-test. (**E**, **E′**) PSA-NCAM immunoelectron microscopy images of new neurons in RMS. The box in (**E**) is magnified in (**E′**). Scale bars: 1 μm (**E**), 200 nm (**E′**). (**F–G′**) Representative transmission electron microscopy images of control (**F**, **F′**) and endoN-treated (**G**, **G′**) new neurons in RMS. Red, purple, and blue arrows indicate AJ-like adhesions, non-adherent areas (closed), and non-adherent areas (open), respectively; the boxes in (**F**, **G**) are magnified in (**F′**, **G′**), respectively. Scale bars: 1 μm (**E**, **F**), 200 nm (**F′**, **G′**). (**H**) Percentage of AJ-like adhesion and non-adherent areas ([open] or [closed]) in the control and endoN-treated groups. Removal of PSA from new neurons decreases the non-adherent area (closed) of new neurons and increases AJ-like adhesion with new neurons; each group $n = 30$ cells; non-adherent areas [open] and [close]; *t*-test, AJ-like adhesion; Wilcoxon rank sum test. Data information: In (**A**, **D**, **H**), data are presented as mean ± SEM. *$P < 0.05$, ***$P < 0.005$, ****$P < 0.001$; adjusted with Bonferroni correction in (**A**) and (**H**).

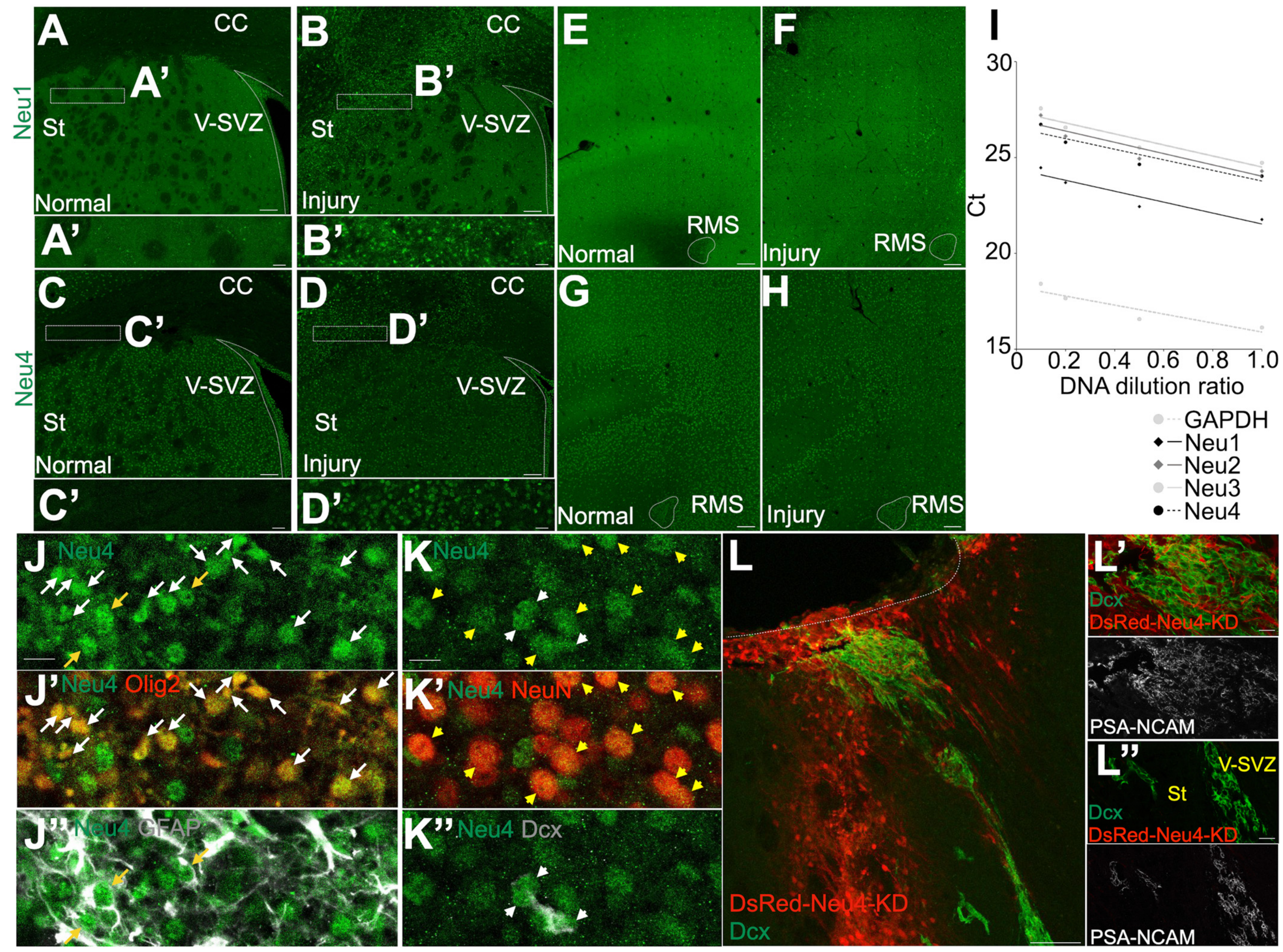

Figure EV3. Neuraminidase expression is increased after brain injury.

(**A–B′**) Representative images of brain sections stained for Neu1 (green) from normal (**A**) and injured (**B**) brain of WT mice. The boxed areas in (**A** and **B**) are enlarged in (**A′** and **B′**), respectively. Scale bars: 100 μm (**A**, **B**), 20 μm (**A′**, **B′**). (**C–D′**) Representative images of brain sections stained for Neu4 (green) from normal (**C**) and injured (**D**) brain of WT mice. The boxed areas in (**C** and **D**) are enlarged in (**C′** and **D′**), respectively. Scale bars: 100 μm (**C**, **D**), 20 μm (**C′**, **D′**). (**E, F**) Representative images of RMS sections stained for Neu1 (green) from normal (**E**) and injured (**F**) brain of WT mice. Scale bars: 100 μm (**E**, **F**). (**G, H**) Representative images of RMS sections stained for Neu4 (green) from normal (**G**) and injured (**H**) brain sections of WT mice. Scale bars: 100 μm (**G**, **H**). (**I**) Evaluation of RT-qPCR primer efficiencies. (**J–J″**) Representative images of injured brain sections of WT mice stained for Neu4 (green), Olig2 (red), and GFAP (white). Merged images of Neu4 and Olig2 in (**J′**) or Neu4 and GFAP in (**J″**). White and yellow arrows indicate Neu4+Olig2+ and Neu4+GFAP+ cells, respectively. Scale bars: 10 μm (**J**). (**K–K″**) Representative images of injured brain sections of WT mice stained for Neu4 (green), NeuN (red), and Dcx (white). Merged images of Neu4 and NeuN in (**K′**) or Neu4 and Dcx in (**K″**). Yellow and white arrows indicate Neu4+NeuN+ and Neu4+Dcx+ cells, respectively. Scale bars: 10 μm (**K**). (**L–L″**) Representative images of brain sections of WT mice injected with Neu4 KD virus 7 dpi and stained for Dcx (green) and DsRed (red) (**L**), or for Dcx (green), DsRed (red) and PSA-NCAM (white) (**L′**, **L″**) 21 dpi. Dotted lines indicate cortical lesion sites. Scale bars: 100 μm (**L**), 20 μm (**L′**, **L″**).

 © The Author(s)

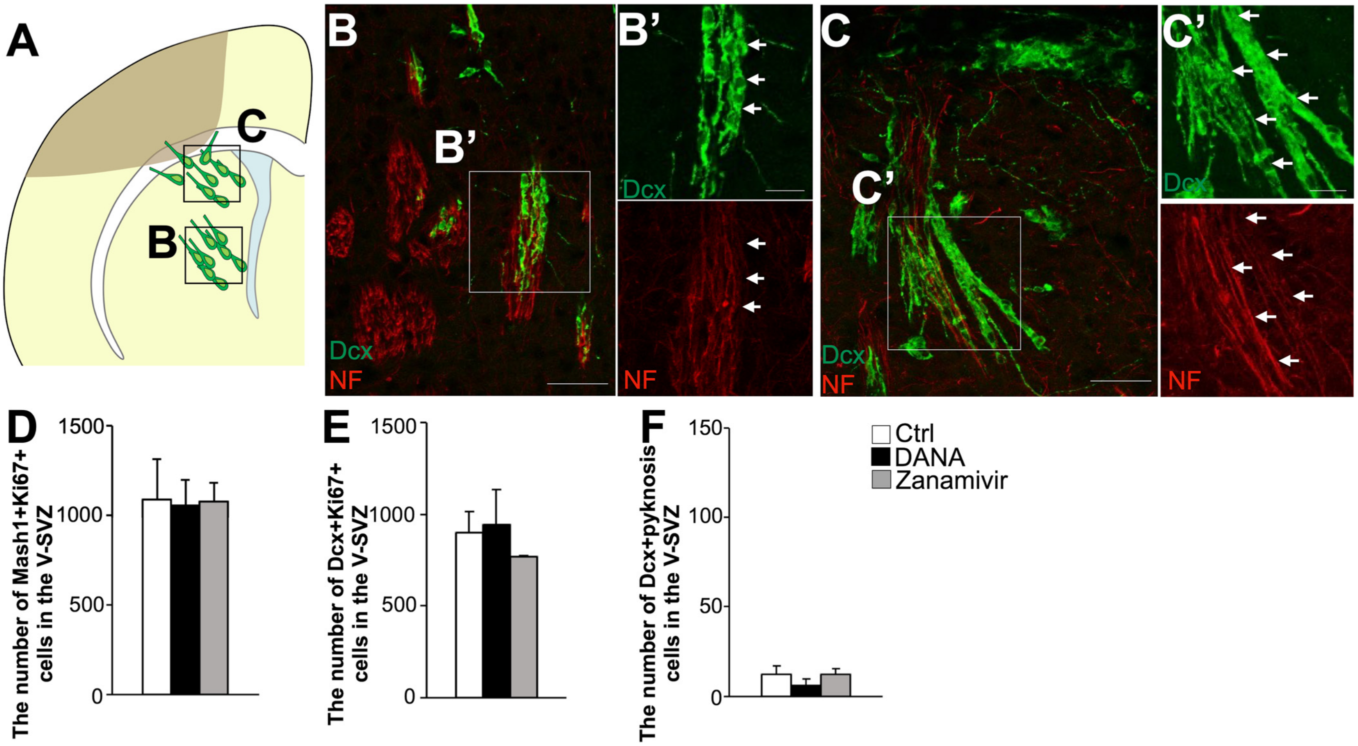

Figure EV4. Neuraminidase inhibitors did not affect proliferation or cell death.

(**A**) Experimental scheme indicating the brain areas (**B**, **C**). (**B–C′**) Representative images of the middle part of the striatum (**B**) and the striatal tissue close to the injured site (**C**) stained for Dcx and NF from DANA-administered WT mice. The boxed areas in (**B**) and (**C**) are enlarged in (**B′**) and (**C′**), respectively. Some new neurons were along the striatal fibers (arrows). Scale bars: 50 μm (**B**, **C**), 20 μm (**B′**, **C′**). (**D**) The number of Mash1 + Ki67+ cells in the V-SVZ in control, DANA, and zanamivir groups; each group $n = 3$ mice; Tukey multiple comparisons of means. (**E**) The number of Dcx+Ki67+ cells in the V-SVZ in control, DANA, and zanamivir groups; each group $n = 3$ mice; Tukey multiple comparisons of means. (**F**) The number of Dcx+ pyknosis cells in the V-SVZ in control, DANA, and zanamivir groups; each group $n = 3$ mice; Tukey multiple comparisons of means. DANA or zanamivir administration in the injured brain did not affect proliferation or cell death. Data information: In (**D**, **E**, **F**), data are presented as mean ± SEM.

© The Author(s)

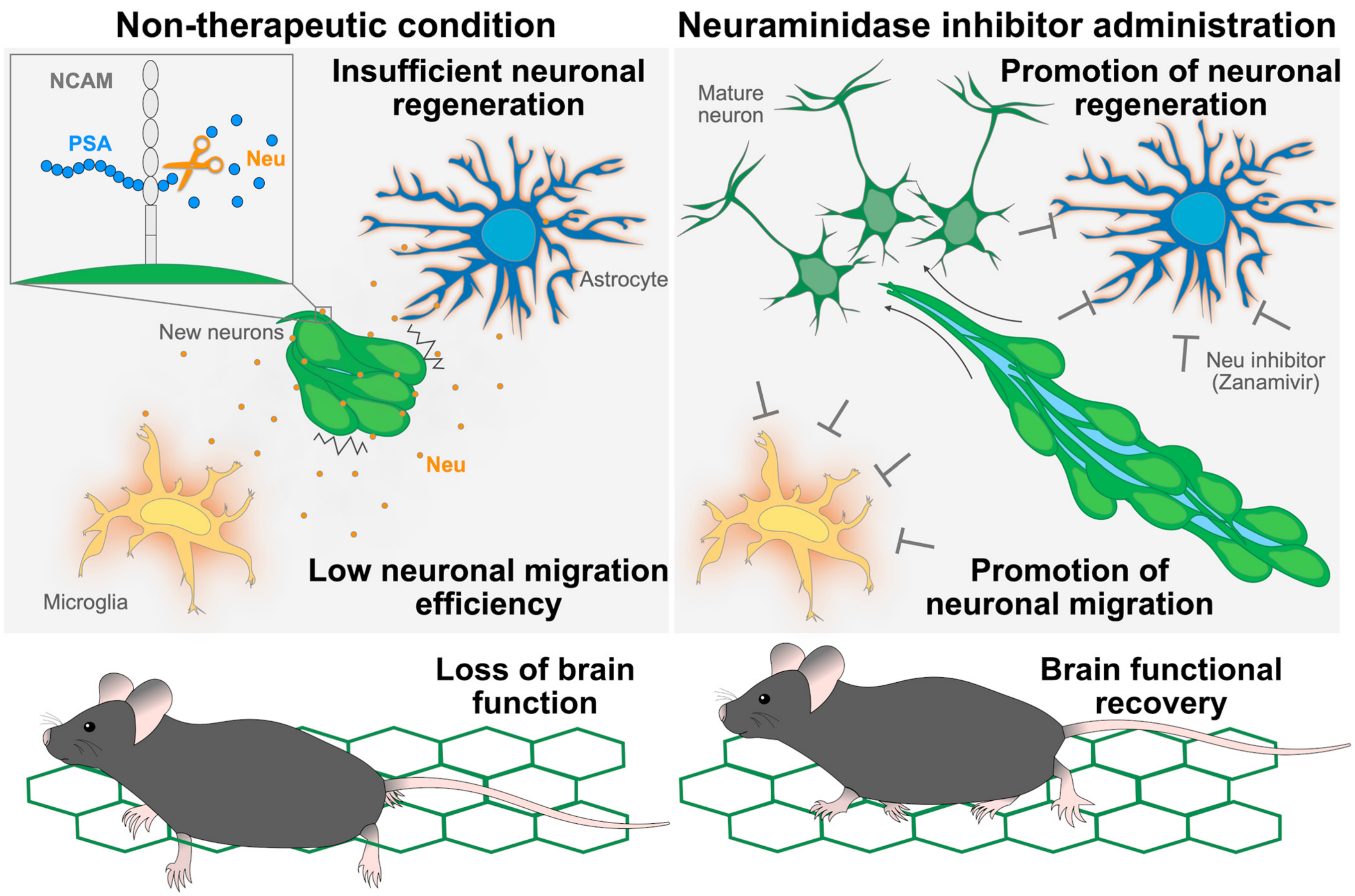

Figure EV5. Neuraminidase inhibitors promote neuronal migration, neuronal regeneration, and brain functional recovery.

In non-therapeutic condition, activated glial cells release neuraminidase after brain injury, and released neuraminidase cleaves PSA of new neurons migrating toward the injured site, reduced PSA causes increased cell adhesion and decreased neuronal migration, and brain function is not recovered. Under condition of neuraminidase inhibitor administration, neuraminidase inhibitor promotes neuronal migration and regeneration, and brain function is recovered.

 © The Author(s)
